# ﻿A taxonomic revision of ten whitefish species from the lakes Lucerne, Sarnen, Sempach and Zug, Switzerland, with descriptions of seven new species (Teleostei, Coregonidae)

**DOI:** 10.3897/zookeys.1144.67747

**Published:** 2023-02-02

**Authors:** Oliver M. Selz, Ole Seehausen

**Affiliations:** 1 Department of Fish Ecology and Evolution, Centre for Ecology, Evolution & Biogeochemistry (CEEB), Eawag: Swiss Federal Institute of Aquatic Science and Technology, 6047 Kastanienbaum, Switzerland Department of Fish Ecology and Evolution, Centre for Ecology, Evolution & Biogeochemistry (CEEB), Eawag: Swiss Federal Institute of Aquatic Science and Technology Kastanienbaum Switzerland; 2 Aquatic Ecology and Evolution, Institute of Ecology and Evolution, University of Bern, 3012 Bern, Switzerland University of Bern Bern Switzerland; 3 Federal Office for the Environment (FOEN), Aquatic Restoration and Fisheries Section, 3011 Bern, Switzerland Federal Office for the Environment (FOEN), Aquatic Restoration and Fisheries Section Bern Switzerland

**Keywords:** Adaptive radiation, *Coregonus*, ecological speciation, evolution, taxonomy

## Abstract

The taxonomy of the endemic whitefish of the lakes of the Reuss River system (Lucerne, Sarnen, Zug) and Lake Sempach, Switzerland, is reviewed and revised. Lake Lucerne harbours five species. *Coregonusintermundia***sp. nov.** and *C.suspensus***sp. nov.**, are described. *Coregonusnobilis* Haack, 1882, *C.suidteri* Fatio, 1885, and *C.zugensis* Nüsslin, 1882, are redescribed. Genetic studies have shown that *C.suidteri* and *C.zugensis* are composed of several distinct species endemic to different lakes. The names *C.suidteri* and *C.zugensis* are restricted to the species of lakes Sempach and Zug, respectively. The whitefish populations previously referred to as *C.suidteri* and *C.zugensis* from Lake Lucerne are described as *C.litoralis***sp. nov.** and *C.muelleri***sp. nov.**, respectively. Furthermore, the whitefish from Lake Zug that were previously referred to as *C.suidteri* are described as *C.supersum***sp. nov**. A holotype is designated for *C.supersum* that was previously one of two syntypes of *C.zugensis*. The other syntype is retained for *C.zugensis*. *Coregonusobliterus***sp. nov.** is described from Lake Zug, and *C.obliterus* and *C.zugensis* from Lake Zug are extinct. Finally, we describe *C.sarnensis***sp. nov.** from lakes Sarnen and Alpnach. *Coregonussuidteri* from Lake Sempach shows strong signals of introgression from deliberately translocated non-native whitefish species, which questions if the extant population still carries a genetic legacy from the original species and thus may need to be considered extinct. *Coregonussuspensus* is genetically partially of allochthonous origin, closely related to the radiation of Lake Constance. It is therefore compared to all known and described species of Lake Constance: *C.wartmanni* Bloch, 1784, *C.macrophthalmus* Nüsslin, 1882, *C.arenicolus* Kottelat,1997, and *C.gutturosus* Gmelin, 1818.

## ﻿Introduction

The European whitefish (*Coregonus* spp.) are a classic example of recent postglacial adaptive radiations spanning the Palearctic temperate zone with several lakes in the boreal, subarctic, or pre-alpine regions harbouring multiple coexisting and closely related species (Steinmann 1950; Hudson et al. 2007). Many of these radiations diversified after the most recent glacial maximum approximately 15,000 years ago and comprise of evolutionary young taxa, with up to six genetically and phenotypically differentiated species occurring in single lakes of the pre-alpine region (Bernatchez 2004; Østbye et al. 2005; Hudson et al. 2007, 2011, 2016; Kottelat and Freyhof 2007; Doenz et al. 2018; Jacobs et al. 2019; Selz et al. 2020; De-Kayne et al. 2022; Frei et al. 2022a, b). The repeated diversification of sympatric whitefish in many lakes have either arisen through a combination of sympatric and allopatric speciation in boreal and subarctic lakes (Scandinavia: Østbye et al. 2005; Præbel et al. 2013) or mainly through sympatric speciation from a hybridogenic ancestral population in pre-alpine lakes (Central Europe: Hudson et al. 2011). The repeated diversification of sympatric whitefish along a benthic to limnetic axis and a depth axis from shallow to deep in lacustrine environments exhibits often parallel patterns of divergence in phenotypic traits related to foraging and trophic ecology (i.e., gill raker number, benthic vs. limnetic feeding ecology, habitat depth partitioning during feeding), physiology (i.e., growth rate), and reproductive ecology (i.e., spawning season and spawning habitat varying along lake depth and along the benthic-pelagic axis) (Fatio 1890; Steinmann 1950; Østbye et al. 2005; Vonlanthen et al. 2009, 2012; Harrod et al. 2010; Lundsgaard-Hansen et al. 2013; Hudson et al. 2016; Doenz et al. 2018; Jacobs et al. 2019; Öhlund et al. 2020; Selz et al. 2020; De-Kayne et al. 2022; Frei et al. 2022a, b). Differentiation in phenotypic traits is often mirrored by strong reproductive isolation among sympatric whitefish species, confirming that ecologically differentiated whitefish occurring in sympatry are generally genetically clearly differentiated species (Douglas and Brunner 2002; Douglas et al. 2003; Hudson et al. 2011, 2016; Vonlanthen et al. 2012; Doenz et al. 2018; Feulner and Seehausen 2018; Jacobs et al. 2019; Selz et al. 2020; De-Kayne et al. 2022; Frei et al. 2022a, b).

Swiss lakes once harboured approximately 35 endemic species of whitefish native to 17 lakes, but due to anthropogenic eutrophication of lakes in the middle of the 20^th^ century one third of this diversity has been lost (Vonlanthen et al. 2012; Hudson et al. 2013; Alexander et al. 2017b). The pre-alpine whitefish radiations originate from a hybridogenic ancestral population comprising of two divergent mitochondrial lineages (Hudson et al. 2011; Winkler et al. 2011). Furthermore, based on large genomic data sets the entire pre-alpine whitefish radiation is monophyletic when compared to the closest relatives from northern Germany and Scandinavia and most of the pre-alpine whitefish radiations form monophyletic groups by lake or lake system (Douglas et al. 2003; Hudson et al. 2011, 2016; Doenz et al. 2018; [Bibr B37][Bibr B16]; Frei et al. 2022a, b; this study).

Part of the pre-alpine whitefish species diversity is still unresolved to date, despite the seminal revisions on European freshwater fish by Kottelat (1997) and Kottelat and Freyhof (2007). We recently evaluated the status of whitefish species of lakes Brienz and Thun, Switzerland, where we recognised seven species, of which four were new to science (Selz et al. 2020). Here we revise the endemic, pre-alpine whitefish species diversity of the Reuss River system (Lucerne, Sarnen, Zug) and Lake Sempach, Switzerland, redescribing three and describing seven species, respectively.

We studied the type material listed by Kottelat (1997) for the described species from lakes Lucerne, Sempach, and Zug (*C.nobilis*, *C.suidteri*, *C.zugensis*) and from Lake Constance (*C.arenicolus*, *C.gutturosus*, *C.macrophthalmus*, *C.wartmanni*). Furthermore, we studied contemporary specimens of all whitefish species from Lake Lucerne and from Lake Sarnen (*C.nobilis*, *C.muelleri*, *C.litoralis*, *C.suspensus*, *C.intermundia*, *C.sarnensis*). We also studied historical specimens of *C.litoralis* and *C.muelleri* from Lake Lucerne, of *C.obliterus*, *C.zugensis*, and *C.supersum* from Lake Zug and of all described species from Lake Constance.

## ﻿Materials and methods

### ﻿Study lakes and whitefish species collection

Type material of all currently valid species (based on Kottelat’s (1997) systematic revision of the nomenclature of Swiss whitefish) was inspected in the collections of the Museum d’Histoire Naturelle, Genève (**MHNG**), Switzerland and in the Steinmann and Seehausen-Eawag collection of Eawag, Switzerland, now curated in the Naturhistorisches Museum Bern (**NMBE**). All contemporary specimens are part of the Seehausen-Eawag collection, which is planned to also be curated at a later stage at the NMBE. For the Steinmann collection some jars contain more than one fish and thus we provide the individual labels of each fish with ‘Eawag’ followed by the individual identification number, next to the NMBE number in brackets.

The different whitefish species in this study derive from different lakes, namely Lake Lucerne (47°01'N, 8°24'E, surface area 113.6 km^2^, max depth 214 m), Lake Sarnen (46°52'N, 8°13'E, surface area 7.5 km^2^, max depth 51 m), Lake Sempach (47°08'N, 8°09'E, surface area 14.5 km^2^, max depth 87 m), Lake Zug (47°07'N, 8°29'E, surface area 38.3 km^2^, max depth 197 m), Lake Constance (47°37'N, 9°23'E, surface area and max depth of Upper Lake Constance 473 km^2^ and 251 m and of lower Lake Constance 63 km^2^ and 46 m depth), Lake Lugano (45°59'N, 8°58'E, surface area 48.7 km^2^, max depth 288) and Lake Maggiore (46°05'N, 08°42'E, surface area 212.35 km^2^, max depth 372).

Contemporary samples of whitefish from the different lakes were obtained in the course of many projects of the Seehausen research group. Contemporary material used here was collected in the years 2004, 2005, 2007, 2011, and 2015 to 2017. Some of the fish were obtained from commercial fisheries catches. Fishing was done with monofilament bottom and pelagic gill nets of various mesh size ranging from 5 mm to 60 mm, and across many depth ranges in the limnetic and benthic habitats of the lakes Lucerne and Sarnen (see protocols in Alexander et al. (2015) and Hudson et al. (2016)). The fish come from three different sampling methods: targeted fishing in all lakes mentioned in this study on known spawning grounds (Hudson et al. 2011, 2016; Vonlanthen et al. 2012; this study), targeted fishing at one spawning site in Lake Lucerne along a depth cline (Hudson et al. 2016) and habitat-stratified fishing of the whole lake during the summer months in Lake Sarnen (Vonlanthen and Périat 2017).

### ﻿Procedures of historical and contemporary specimen collection

The collection of contemporary specimens differed slightly between projects, but in general was as follows: upon capture, fish were anaesthetised and subsequently euthanised with appropriate concentrations of phenoxyethanol, clove oil or tricaine methanesulfonate (**MS222**) solutions. Muscle tissue and scales below the dorsal fin, as well as a part of the pectoral fin were taken for genetic and isotopic analysis and to determine the age of each fish. Each fish was photographed and then fixed in 4% formalin solution for at least 1 month and afterwards transferred through a series of ethanol of increasing concentration (30%, 50%) to the final concentration of 70% for storage. Permits for collecting fish in the lakes were issued by the cantons adjacent to the lakes. In the field the fish were identified to species level as good as possible. Sex and fresh mass were noted. Immature fish, where the sex could not be determined externally, were examined internally. For the historical specimens the sex could only be determined when the abdominal cavity had already been opened. The age was determined by counting the annual growth rings of four scales under a confocal microscope following Lehtonen and Nylund (1995).

### ﻿Genetics

We used a data set of 1071 specimens genotyped at ten microsatellite loci. We combined existing data from Hudson et al. (2016) for the whitefish species of Lakes Lucerne, Neuchâtel, Zürich, Walen, and Constance, and from Selz et al. (2020) for the whitefish species from lakes Thun and Brienz with our new genetic data for the whitefish from Lakes Sarnen, Sempach, and Zug. To be able to combine the data set of Hudson et al. (2016) with our data (Selz et al. 2020; this study), we extracted and genotyped 32 samples from Hudson et al. (2016) to be able to verify that genotyping was consistent across allele scoring panels, scorers, and sequencing machines. We found high genotyping agreement. Detailed information about DNA extractions, microsatellite amplification and scoring can be found in Selz et al. (2020).

To identify genetic clusters (K) of populations and obtain genetic assignment proportion for individuals in a dataset with all whitefish species from Lakes Lucerne, Sarnen, Sempach, and Zug we used an individual-based Bayesian clustering approach implemented in STRUCTURE (v. 2.3.4; Pritchard et al. 2000) with 200’000 burn-in steps and 300’000 MCM steps and 10 runs using the admixture and correlated allele frequency model for each assumed K from 1 to 10. The most likely K was determined with the Evanno method by comparing the LnP(D) values from 10 runs of different K values in Structure harvester (Earl and vonHoldt 2012). Deviations from Hardy-Weinberg equilibrium (HWE, 1000 permutations) for all loci per population, multilocus neutral genetic diversity within populations and pairwise genetic differentiation between populations (Fst, 1000 permutations) were calculated with Genodive (v. 3.03; Meirmans and van Tienderen 2004) (Suppl. material 1: table S1). A population-based neighbour-joining tree using Cavalli-Sforza chord distances (D_CH_) was reconstructed using the software package PHYLIP 3.69 (Felsenstein 2005) and the best-tree with bootstrap support for each node (1000 replicates) was visualised with the package ape (5.3) in R (R 3.5.1; R core Team 2018).

### ﻿Morphological and meristic characters

**Figure 1.**
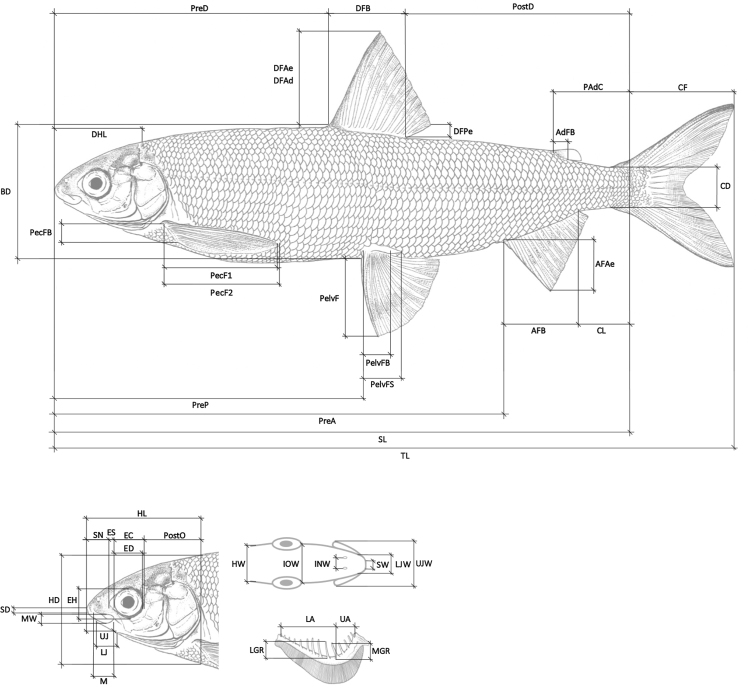
Illustration of the morphological body and head characters (see Table 1 for an explanation of the acronyms and a detailed description of each character).

Twenty-five body, 19 head, four gill, and 12 meristic characters were obtained on 46 historical and 116 contemporary specimens with a digital calliper to the nearest 0.1 mm. When possible, measurements and counts were taken on the left side of the fish, unless a specific character was missing or deformed, in which case that character was obtained from the right side of the fish. The mean of two measurements was taken for each character, whereby the difference between two measurements had to be less than 5%. If deviance exceeded 5%, the distance was measured another two times. Over all morphological characters combined, the average deviance between two measurements was 1.5%. Not all measurements could be taken on all the historical specimens since some characters where damaged or absent, resulting in incomplete character lists for certain specimens. Characters for which we had missing values were excluded from the multivariate ratio analyses (see below). The number of characters used for each analysis is explicitly mentioned in the results section. A description and illustrations of each character can be found in Table 1 and Fig. 1. For all meristic characters the mode and for all morphological characters the mean together with the range for each species are reported. If the sample size was too small, a mode or mean could not be calculated and we report ‘na’ or write out the values. For the described species, the holotype is included in the range. Both sexes are included for the full range of each character of each species. Regarding pigmentation, we refer to the overall melanisation degree of the fins and the body. There is no detailed description of the exact structure of the pigmentation since we have only looked at traits that would be also possible for practitioners to measure in the field.

**Table 1.** Morphological and meristic characters, their abbreviations, and a brief description of each character.

Morphological characters	Abbreviation	Description
**Body**		
Pelvic fin base	PelvFB	Length between insertions of fin
Pelvic fin „spine“ length	PelvFS	Length from upper insertion point of fin to tip of spine; the spine is actually an elongated scale structure
Pelvic fin length	PelvF	Length from upper insertion point of fin to tip of longest unbranched ray
Pectoral fin base	PecFB	Length between insertions of fin
Pectoral fin 1 length	PecF1	Length from upper insertion point of fin to tip of unbranched ray
Pectoral fin 2 length	PecF2	Length from upper insertion point of fin to tip of longest branched ray
Dorsal fin base	DFB	Length between insertions of fin
Length of anterior part of dorsal fin erected	DFAe	Length from anterior insertion point of fin to tip of longest unbranched ray, when fin is fully erected
Length of anterior part of dorsal fin depressed	DFAd	Length from anterior insertion point of fin to tip of longest unbranched ray, when fin is depressed
Length of posterior part of dorsal fin erected	DFPe	Length from posterior insertion point of fin to tip of most posterior ray, when fin is erected
Anal fin base	AFB	Length between insertions of fin
Length of anterior part of the anal fin	AFAe	Length from anterior insertion point of fin to tip of longest ray, when fin is fully erected
Adipose fin base	AdFB	Length between insertions of fin
Caudal fin length	CF	Length from the middle of hypural plate of the caudal fin (internally the expanded bones at the end of the backbone that support the caudal fin, externally where the lateral line scales end) to the tip of the longest unbranched ray either being on the dorsal or ventral part of the caudal fin
Caudal peduncle depth	CD	Vertical distance between dorsal and ventral margins of the caudal peduncle at its narrowest part
Caudal peduncle length	CL	Length from posterior insertion point of anal fin to the middle of the hypural plate of the caudal fin
Length from posterior part of adipose fin to caudal fin base	PAdC	Length from anterior insertion point of adipose fin to the middle of the hypural plate of the caudal fin
Dorsal head length	DHL	Length from tip of snout to most posterior part of the frontal head bone
Prepelvic length	PreP	Length from tip of snout to anterior insertion point of pelvic fin
Preanal length	PreA	Length from tip of snout to anterior insertion point of anal fin
Standard length	SL	Length from tip of snout to the middle of the hypural plate of the caudal fin
Total length	TL	Length from tip of snout to the tip of longest unbranched ray either being on the dorsal or ventral part of the caudal fin
Predorsal length	PreD	Length from tip of snout to anterior insertion point of dorsal fin
Body depth	BD	Vertical distance between dorsal and ventral margins of body from anterior insertion point of dorsal fin to anterior insertion point of anal fin: not necessarily the greatest body depth
Postdorsal length	PostD	Length from posterior insertion point of dorsal fin to middle of hypural plate of the caudal fin
**Head**		
Eye diameter	ED	Horizontal distance across the midline of the eye from the anterior to the posterior margin of the soft eye tissue
Eye cavity	EC	Horizontal distance across the midline of the eye from the anterior margin of the eye socket to the posterior margin of the eye cavity
Eye height	EH	Vertical distance across the midline of the eye from the dorsal margin of the eye cavity to the ventral margin of the eye cavity
Eye socket	ES	Horizontal distance from the anterior margin of the eye socket to the most anterior point of the posterior margin of the eye socket
Postorbital length	PostO	Length from posterior margin of the eye to the most posterior point of the operculum
Head length	HL	Length from the tip of snout to most posterior point of the operculum margin
Head depth	HD	Transverse distance between margins at the widest point of the head.
Head width	HW	Distance between the anterior margins of the left and right operculum
Mouth width	MW	Transverse distance between margins of the upper and lower jaw
Upper jaw length	UJ	Length from the tip of the snout to most posterior point of the upper jaw
Lower jaw length	LJ	Length from the most anterior point of the lower jaw to the lower jaw insertion
Lower jaw width	LJW	Length between the posterior left and right side of the lower jaw
Uper jaw width	UJW	Length between the anterior left and right point of the upper jaw
Length of maxilla	M	Length from the most anterior point of the maxilla to the most posterior point of the maxilla
Snout length	SN	Length from tip of snout to anterior margin of the eye
Snout depth	SD	Vertical distance from the upper to the lower margin of the rostral plate
Snout width	SW	Horizontal distance from the left to the right margin of the rostral plate
Interorbital width	IOW	Distance between the anterior margin of the left and right eye cavity
Internarial width	INW	Distance between the right and left nostrils
**Gill**		
Upper arch length	UA	Length of the first hypobranchial (upper arch) from the most anterior point to the joint of the hypo- and ceratobranchial where the middle raker emerges
Lower arch length	LA	Length of the first ceratobranchial (lower arch) from the most posterior point to the joint of the hypo- and ceratobranchial where the middle raker emerges
Middle gill raker length	MGR	Length of the gill raker directly at the joint of the hypo- and ceratobranchia, from the insertion of the gill raker to the tip of the gill raker
Longest gill raker length	LGR	Length of the longest gill raker on either the hypo- and ceratobranchia, from the insertion of the gill raker to the tip of the gill raker
**Mersitic characters**	**Acronym**	**Description**
Pelvic fin rays	PelvFR	Number of unbranched and branched rays
Pectoral fin rays	PecFR	Number of unbranched and branched rays
Dorsal fin rays	DFR	Number of unbranched and branched rays; the posterior most dorsal rays are often borne from a single pterygiophore (the bones on which the rays articulate), in such a case the two rays are counted as 2 rays, rudimentary unbranched rays at the anterior part of the fin are counted
Anal fin rays	AFR	Number of unbranched and branched rays; the posterior most anal rays are often borne from a single pterygiophore (the bones on which the rays articulate), in such a case the two rays are acounted as 2 rays, rudimentary unbranched rays at the anterior part of the fin are counted
Lateral line scales	LS	Scales bearing the lateral-line column canal from the head to the end of the hybpural plate of the caudal peduncle
Predorsal scales	PDS	Dorsal scales starting from the posterior end of the head to the anterior insertion of the dorsal fin
Transverse dorsal scales	TDS	Number of scale rows between anterior insertion of the dorsal fin and the lateral line, not accounting for the lateral line scale and the scale on the dorsal midline (in front of the dorsal fin)
Transverse anal scales	TAS	Number of scale rows between anterior insertion of the anal fin and the lateral line, not accounting for the lateral line scale and the scale on the ventral midline (in front of the anal fin)
Transverse pelvic scales	TPS	Number of scale rows between anterior insertion of the pelvic fin and the lateral line, not accounting for the lateral line scale and the scale on the ventral midline (in front of the pelvic fin)
Upper arch gill raker number	UGR	Number of gill rakers on first upper arch; all rakers including rudimentary developed rakers
Lower arch gill raker number	LGR	Number of gill rakers on first lower arch; all rakers including rudimentary developed rakers and the middle raker
Total gill raker number	total GR	Gill raker number of upper and lower arch combined

### ﻿Morphological analyses

Since several species are extinct (*C.obliterus*, *C.zugensis*, *C.suidteri*, *C.gutturosus*), which has been attributed to anthropogenic-induced eutrophication of many lakes in Switzerland in the middle of the 20^th^ century with subsequent environmental changes that had effects on the morphological characters of whitefish species (e.g., gill rakers) (Vonlanthen et al. 2012; Frei et al. 2022a, b), we didn’t use the historical and contemporary specimens together to diagnose the species. Hence, specimens from contemporary samples are used to diagnose and distinguish among and between the whitefish species of lakes Lucerne and Sarnen. Specimens from historical samples are used to diagnose and distinguish among all historical specimens from Lake Zug, as well as between historical specimens of the species from the lakes Lucerne, Sempach, and Zug that were previously all grouped under *C.zugensis* or *C.suidteri*. Furthermore, specimens from historical samples of Lake Constance are compared to specimens from contemporary samples of *C.suspensus* from Lake Lucerne.

We used multivariate ratio analysis methods in R to perform linear discriminant analysis (LDA) on morphological ratios (Baur and Leuenberger 2011; Baur et al. 2014). Analysis of morphological ratios are especially well suited in a taxonomic context (László et al. 2013). For a pairwise species comparison we ran linear discriminant analysis (**LDA**) on all or a part of the measured characters to calculate the first two LDA ratios of characters that best separate the two species in each of the pairwise species’ comparisons. This was done among all contemporary specimens from lakes Lucerne and Sarnen and among all historical specimens from Lake Zug, as well as between historical specimens of the species from the lakes Lucerne and Zug that were previously all grouped under *C.zugensis* or *C.suidteri*. This method also allows to estimate the extent of shape change with size (i.e., the contribution of allometry to these ratios) which is given as δ and describes how good shape discriminates in comparison to size (see Baur and Leuenberger 2011: 818, formula 14). In several pairwise species comparisons, we had more variables than individuals, which will not allow to calculate the best LDA ratios. In such cases we used a subset of informative characters to match the number of individuals. All the comparisons with a subset of characters are marked in the table and the respective characters that were used are listed (Tables 10, 11). We report only ratios that have shown to have little overlap and thus may be used to distinguish the species. Ratios marked in the table with an asterisk (*) have very little or no overlap with other species and could thus be used in the identification key and the species diagnoses.

### ﻿Macrofossils from sediment cores

We collected and analysed seven sediment cores from Lake Sarnen that covered the periods (and beyond) of documented historical introductions of allochthonous whitefish from lakes Lucerne, Sempach, and Zug into Lake Sarnen (1888–1900: Heuscher 1900; 1913–1920: Steinmann 1950). All seven cores were collected at the deepest location (51 m) of Lake Sarnen. The cores were cut in half and photographed. For the analysis the cores were divided into 2.5 cm intervals (~ 0.26 litres of sediment) for an average time interval of 3.6 years at a net sedimentation rate of 0.69 cm/year, which was obtained from γ-ray measurements of 210Pb and 137Cs (Müller et al. 2021). We analysed the cores from 50 cm downcore to the bottom of each core. The sediment was sieved through a 250 μm mesh and subsequently examined under a stereomicroscope. Sieve fractions were primarily screened for fish scales and bone fragments. Scales were photographed and compared to reference images from the ‘Atlas of Fish Scales’ Vol. 2 (Lehtonen and Nylund 1995).

### ﻿Data accessibility

All the data from this manuscript is available at Dryad Data Repository (https://doi.org/10.5061/dryad.8cz8w9gvx). The specimens used for taxonomic work in this study and in Selz et al. (2020) have been processed and given an accession number by the Naturhistorisches Museum Bern (**NMBE**). However, many of the specimens used for genetic work in this study have yet to be processed by the NMBE and are thus labelled in the accompanied dataset to this study with unique identifiers from the Seehausen-Eawag laboratory.

## ﻿Results

### ﻿Genetics

We found K = 4 to be the most likely genetic cluster in the STRUCTURE analysis for the whitefish species of lakes Lucerne, Sarnen, Sempach, and Zug (Suppl. material 1: fig. S2). In agreement with previous work by Hudson et al. (2011) on a large AFLP data set we find that Lake Lucerne consists of two genetic clusters. One cluster (i.e., ‘cluster 1’ in Hudson et al. 2011) contains the species *C.nobilis* and *C.muelleri* and one cluster (i.e., ‘cluster 2’) contains the species *C.litoralis*, *C.intermundia*, *C.suspensus* and the whitefish population from Lake Alpnach. The two additional clusters in our analysis are a cluster (i.e., ‘cluster 3’) containing all whitefish from Lake Zug and Sempach and a cluster (i.e., ‘cluster 4’) containing the species *C.sarnensis* from Lake Sarnen. Few individuals from Lake Sempach that grouped in cluster 3 show high assignment likelihoods to cluster 2 of Lake Lucerne and vice versa. We have also identified a few individuals in Lake Sarnen that had moderately high individual assignment likelihoods to the Lake Lucerne cluster 2 and a few individuals in Lake Alpnach, a side-arm of Lake Lucerne that is connected to Lake Sarnen, that had high individual assignment likelihoods to the Lake Sarnen cluster 4 (Suppl. material 1: fig. S2). This can be attributed to the genetic legacy of deliberate cross-lake introductions of different whitefish species in the last two centuries (Heuscher 1900; Steinmann 1950). To compare our data to previous studies (Hudson et al. 2016) we built a population-based neighbour-joining tree that contained all samples of Hudson et al. (2016) but additionally our new samples from lakes Sarnen, Zug and Sempach, Thun, and Brienz. We replaced the samples from Hudson et al. (2016) for the lakes Thun and Brienz with new samples and additional species described by Selz et al. (2020). Congruent with Hudson et al. (2016) the species group mostly by lake or lake-system, i.e., into lake-system specific species-flocks (Suppl. material 1: fig. S2). The two species, *C.acrinasus* (Selz et al. 2020) from Lake Thun and *C.suspensus* from Lake Lucerne, for which previous work (Douglas and Brunner 2002; Douglas et al. 2003; Bittner 2009; Hudson et al. 2011, 2016; Doenz et al. 2018; De-Kayne et al. 2022) has suggested that the two species have genetic ancestry contributions from whitefish of Lake Constance, group with Lake Constance species. The few individuals identified in the STRUCTURE analysis from Lake Sarnen that have moderately high individual assignment likelihoods to the Lake Lucerne cluster 2, group in the neighbour-joining tree with one species from that cluster, namely *C.litoralis*. The few individuals from Lake Alpnach, which had high individual assignment likelihoods to the Lake Sarnen cluster 4 group in the neighbour-joining tree with *C.sarnensis*. The samples from lakes Sempach and Zug that grouped in cluster 3 also group in the neighbour-joining tree next to each other. Genetic differentiation (Fst) between the species ranged from 0.02 to 0.4 (Suppl. material 1: table S2).

### ﻿Macrofossils from sediment cores in Lake Sarnen

The longest core of seven cores ranged back to the year 1849 and the oldest whitefish scales that were found date back to the years 1861–1857, at least 27 years prior to the deliberate introductions from 1888 to 1920 of alevins, fry, and adult whitefish from Lake Lucerne, Sempach, and Zug (Suppl. material 1: fig. S1). These findings together with the genetics analyses suggest that *C.sarnensis* is an endemic species of Lake Sarnen.

### ﻿Species descriptions

#### Lake Lucerne whitefish

##### ﻿ Coregonus litoralis sp. nov.

Taxon classificationAnimaliaSalmoniformesCoregonidae

2BC656D0-989C-5E30-9AA2-265C716E74DA

https://zoobank.org/24E4AC0C-792C-4911-8E18-167FFA164303

Figs 3 , 14 , Tables 2 , 10 , 11 , 12 , 13 , 14 , Suppl. material 1: figs S2, S3, tables S1–S3

Coregonus ‘Ballen’: Douglas and Brunner 2002; Douglas et al. 1999.
Coregonus ‘Balchen’: Steinmann 1950; Douglas and Brunner 2002.
Coregonus ‘Grossfelchen’: Svarvar and Müller 1982.
Coregonus lavaretus nat. *riusensis*, oekot. *primigenius*: Steinmann 1950 (see also synonymy of C.sarnensis and C.supersum).
Coregonus schinzii supersum var. lucernensis : Fatio 1890; Birrer and Schweizer 1938 (see also synonymy of C.supersum).
Coregonus sp. ‘Bodenbalchen’: Hudson et al. 2011, 2013; Ingram et al. 2012; Vonlanthen et al. 2012; Lundsgaard-Hansen et al. 2013; Roesch et al. 2013; Alexander et al. 2017a.
Coregonus sp. ‘large’: Hudson et al. 2016.
Coregonus suidteri : Kottelat 1997; Kottelat and Freyhof 2007 (see also synonymy of C.supersum and C.suidteri).

###### Material examined.

***Holotype*.** Contemporary specimen (year: 2007): NMBE-1078103, 326 mm SL, male; Switzerland: Lake Lucerne. ***Paratypes*.** All from Switzerland, Lake Lucerne: Contemporary samples (years: 2007, 2008): NMBE-1078064, NMBE-1078075, NMBE-1078079, NMBE-1078080, NMBE-1078083, NMBE-1078085, NMBE-1078091, NMBE-1078092, NMBE-1078098, NMBE-1078102-105, *N* = 12, 304–363 mm SL; Historical specimens (years: 1890, 1899, 1939): MHNG-717.046, NMBE-1076293-295 (Eawag-3081-1, Eawag-3081-2, Eawag-310-1, Eawag-310-2, Eawag-310-3, Eawag-309), *N* = 7, 222–392 mm SL.

###### Diagnosis.

*Coregonuslitoralis* is a large whitefish (standard length at 3 years of age: range = 325–392 mm, mean = 353 mm) with strong pigmentation of all fins and body; greenish blue colour that sometimes has a pale mustard yellow undertone on the flanks above the lateral line; moderate to many pigmented small dots on the scales along the flank and the dorsum; deep bodied (23.4–30% SL, mean = 26.9); blunt snout; short head (19.2–20.9% SL, mean = 19.9); sub-terminal mouth; small eye (eye diameter: 18.2–21.4% HL, mean = 20.2) with a thick and triangular-shaped eye socket; short and stout caudal peduncle (caudal peduncle depth: 7.5–8.4% SL, mean = 8.1; caudal peduncle length: 11.5–14.3% SL, mean = 12.6); few and short gill rakers (longest gill raker: 9.2–13.1% HL, mean = 11; total gill raker number: 24–32).

###### Description.

***Shape***: Generally deep bodied with greatest body depth anterior of the dorsal fin. Dorsal profile from tip of snout to anterior origin of dorsal fin is strongly convex and ventral profile moderately convex from interorbital area to pelvic fin origin. Head short. Mouth short and sub-terminal. Rostral plate pronounced and a bit wider than deep resulting in a slightly rectangular shape. Tip of snout often blunt. Small eye with a thick and sickle cell-shaped eye-socket. Pectoral fin moderately tapered and moderately short. Dorsal fin long with anterior unbranched ray of the erected dorsal fin mostly 60–70 ° angle to body axis and slightly bent posteriorly at the end of the ray. Dorsal fin longest anteriorly and progressively shortening posteriorly with the outer margin of dorsal fin straight. Caudal peduncle stout and short with caudal fin moderately forked. Unbranched rays of anal fin slightly bent posteriorly. Anal fin longest anteriorly and progressively shortening posteriorly with the outer margin of anal fin mostly straight and only rarely slightly concave. ***Meristics***: Few and short gill rakers. ***Colour***: Pigmentation of fins and body overall strong in live specimens. Pectoral fin moderately pigmented at median to distal parts of the fin. All other fins are strongly pigmented. Silvery appearance along flanks with moderate to many pigmented small dots (aggregation of melanophores) on the scales along flank and dorsum. Distribution of dots bound to scale patterning such that dots are found at edge of the scales or at boundary point of two scales. Dorsally above lateral line the silvery appearance changes to a pale greenish or dark greenish blue colour. Seldomly, especially in older specimens, general greenish blue colour can have a pale mustard yellow ground colour (see also colour description in Fatio (1890) and Birrer and Schweizer (1938)). Dorsal part of head and snout around nostrils is strongly pigmented. Pre-operculum and operculum are silvery with one black spot on lower margin of the pre-operculum. Preserved specimens are pale in colouration with similar pigmentation as described for live specimens. Silvery, translucent, not coloured or unpigmented parts of body brown-yellowish, whereas pigmented parts conserved and coloured parts (dorsally above the lateral line) brownish.

###### Differential diagnosis.

Based on contemporary specimens the total number of gill rakers of 24–32 (modes = 26 and 30) distinguishes *C.litoralis* from three of five of the other whitefish species of Lake Lucerne by having fewer gill rakers than the other species (34–40, mode = 38 in *C.nobilis*, 33–37, mode = 35 in *C.suspensus*, 33–43, mode = 36 in *C.muelleri*) (Table 13). Furthermore, the average standard length of three old contemporary specimens distinguishes *C.litoralis* from all other whitefish species of Lake Lucerne except for *C.nobilis*, where only specimens of 5 years of age or older were caught. The average standard length at 3 years of age of *C.litoralis* (range = 325–392 mm, mean = 353 mm) is larger than that of *C.muelleri* (160–232 mm, 194 mm), *C.suspensus* (266–315 mm, 289 mm) and *C.intermundia* (243–300 mm, 273 mm) (Suppl. material 1: table S3). The differential diagnoses against contemporary specimens of *C.sarnensis* from Lake Sarnen and against historical specimens of the three whitefish species from Lake Zug and *C.suidteri* from Lake Sempach are given under those species’ account.

*Coregonuslitoralis* – *Coregonusintermundia*

The specimens of *C.litoralis* differ from those of *C.intermundia* in being deeper bodied (23.4–30% SL, mean = 26.9 vs. 21.6–27.1% SL, mean = 23.9) and having a smaller eye (eye diameter: 18.2–21.4% HL, mean = 20.2 vs. 20.4–23.3% HL, mean = 22.1) (Tables 2, 3). Based on ratios *C.litoralis* can be distinguished from *C.intermundia* by a larger ‘predorsal length / eye diameter’ ratio (PreD/ED: 10.89–12.75 vs. 9.92–10.59) and a larger ‘snout length / maxilla length’ ratio (SN/M: 1.17–1.39 vs. 1.02–1.17) (Table 10).

**Table 2.** Morphological and meristic data of *Coregonuslitoralis* from Lake Lucerne, NMBE-1078097 holotype, contemporary specimen; paratypes of contemporary specimens *N* = 12 and paratypes of historical specimens *N* = 7. For males and both sexes the holotype is included in the range and mean of the contemporary specimens.

Species	*Coregonuslitoralis*								
Morphological characters	Contemporary specimens							Historical specimens	
	Holotype	Holotype + Paratypes		Paratypes		Holotype + Paratypes			
		Ntotal = 13 *		Nfemales = 5 **		Nmales = 8		Ntotal = 7 ***	
		mean	range	mean	range	mean	range	mean	range
**Standard length (mm)**	326	328	304–363	322	304–337	332.5	312–363	321.5	222–392
**Percentage of standard length**									
Pelvic fin base	4.8	4	3.6–4.8	4	3.6–4.4	4.6	4.1–4.8	4.6	3.9–5.3
Pelvic fin ‚spine‘ length	7.7	6.3	5.3–7.7	6.2	5.7–6.6	6.5	5.3–7.7	6.3	5.2–6.7
Pelvic fin length	16.5	15.9	14.2–17.2	15.4	14.2–16.3	16.3	15.4–17.2	17.3	14.9–21.5
Pectoral fin base	3.7	3.4	3–3.7	3.3	3–3.6	3.4	3.1–3.7	3.5	3.1–3.9
Pectoral fin 1 length	16.2	15.8	14.3–17	15.5	14.3–16.5	16	15.3–17	17.4	14.5–21.6
Pectoral fin 2 length	17.4	16.7	15–17.9	16.2	15–17.4	16.9	15.9–17.9	17.5	15.2–21.4
Dorsal fin base	13.4	13.4	11.9–15.1	13	11.9–13.6	13.7	12.6–15.1	11.7	9–13.2
Length of anterior part of dorsal fin erected	19.3	18.9	16.9–20.9	18.7	16.9–19.8	19.1	17.3–20.9	19.1	17.1–23.1
Length of anterior part of dorsal fin depressed	20.5	20.4	17.6–22.6	20	17.6–21.1	20.7	18.7–22.6	20.2	18.3–23.4
Length of posterior part of dorsal fin erected	5.8	5.5	4.5–6	5.1	4.5–5.6	5.8	4.8–6	5.9	5.5–6.5
Anal fin base	12.4	12.8	11.3–14.4	12.2	11.3–13.4	13.2	12.4–14.4	11.5	9.5–13.3
Length of anterior part of the anal fin	13.2	12.9	11.1–14.1	12.6	11.1–13.6	13.1	12.4–14.1	12.7	10.3–15.9
Adipose fin base	5.6	5	3.8–5.8	5.1	4.5–5.5	4.9	3.8–5.8	5.7	4.4–7.8
Caudal fin length	24.1	23.2	20.8–24.4	23.1	23–23.3	23.2	20.8–24.4	20.7	na
Caudal peduncle depth	8.4	8.1	7.5–8.4	8	7.5–8.3	8.1	7.6–8.4	7.7	6.9–8.5
Caudal peduncle length	12.1	12.6	11.5–14.3	13.1	12.3–14.3	12.2	11.5–13.1	13.9	12–15.4
Length from posterior part of adipose fin to caudal fin base	18.7	18.2	16.7–19.8	18.7	17.3–19.8	17.9	16.7–18.7	19.3	15.8–22.9
Dorsal head length	15.2	14.3	13.2–15.2	14	13.2–14.3	14.6	14–15.2	15.2	14.4–17.9
Prepelvic length	52.2	50.9	47.8–54.2	51.1	50–52.1	50.7	47.8–54.2	52.0	45.5–62.3
Preanal length	77	76.5	75.3–78.9	77.2	76–78.9	76.1	75.3–77	78.7	73–93.5
Predorsal length	49.9	46.5	43.5–49.9	46	43.5–49.1	46.8	45.5–49.9	50.2	46.3–58.5
Body depth	26.6	26.9	23.4–30	28.1	26–30	26.1	23.4–27.5	26.2	21.8–29.9
Postdorsal length	40.2	42.1	40–45.2	42.9	40.4–45.2	41.7	40–43.6	44.7	39.9–48.3
Head length	20.7	19.9	19.2–20.9	19.8	19.2–20.4	20	19.2–20.9	21.9	20.9–24.7
Total length	121.2	119.9	117.7–121.8	119.9	118.8–121	119.9	117.7–121.8	118.5	na
**Head length (mm)**	67.5	65.5	59.4–69.6	63.8	59.4–66.5	66.5	62.1–69.6	65.6	46.6–82.1
**Percentage of head length**									
Snout length	24.5	24.1	22.1–25.9	24.2	22.1–25.9	24.1	23.3–24.5	23.4	19–29
Eye diameter	20	20.2	18.2–21.4	20.2	19.3–20.8	20.2	18.2–21.4	20.4	17.9–22.7
Eye cavity	24.3	24.6	22.7–26	24.6	23.9–25.7	24.5	22.7–26	26.0	24.1–27.7
Eye height	20.8	21.5	20.2–23.7	21.5	20.6–22.8	21.5	20.2–23.7	22.2	20.3–24.8
Eye socket	4.9	4.1	3–4.9	4.1	3.8–4.5	4.1	3–4.9	4.6	3.7–5.7
Postorbital length	52.1	52.9	51.1–55	53.6	52.6–55	52.5	51.1–54.5	53.5	51.6–56.2
Head depth	71.3	73.8	70.1–77.9	73.4	70.1–76.7	74.1	71.2–77.9	72.5	68.1–77.1
Mouth width	9.4	9.4	8.6–10.1	9.5	8.8–10.1	9.3	8.6–9.8	10.2	8.6–11.3
Upper jaw length	26.7	26.6	25.3–29.6	26.8	25.6–29.6	26.4	25.3–27.6	27.8	25.6–30.7
Lower jaw length	40.9	39.6	36.8–42.5	39.7	36.9–42.5	39.6	36.8–41.6	39.4	36.9–44.5
Length of maxilla	19.7	19.1	17.4–20.5	19.4	17.9–20.5	18.8	17.4–20.4	20.5	18.7–23
Snout depth	10.1	8.6	6.9–10.5	8.4	6.9–10.5	8.7	7.6–10.3	9.1	7.3–11.4
Snout width	15.2	14.9	13.1–17.2	14.8	13.5–15.5	15	13.1–17.2	15.3	13.2–18.8
Head width	48.7	49.1	46.4–51.2	49.1	47.6–50.5	49.1	46.4–51.2	50.1	45–55
Interorbital width	28.8	28.2	26.3–29.8	27.8	26.3–28.8	28.5	26.8–29.8	28.1	23.5–31.3
Internarial width	13.5	12.4	11–13.5	12.4	11.3–13.5	12.3	11–13.5	12.9	10.7–14.5
Lower jaw length	8.1	8	6.7–8.8	7.8	6.7–8.8	8.1	7.3–8.7	8.1	6.9–9.2
Lower jaw width	22.4	21.7	20.7–24.3	21.3	20.7–21.7	22	20.8–24.3	23.1	21.7–25.3
Middle gill raker length	9.1	10	7.8–11.8	10.5	9.2–11.8	9.7	7.8–11.4	11.1	8.9–13.4
Longest gill raker length	10.1	11	9.2–13.1	11.6	9.4–13.1	10.6	9.2–12.2	11.7	9.1–14.2
Upper arch length	15.5	17.3	14.9–19.9	17.6	14.9–19.9	17.1	15.5–19.5	17.7	na
Lower arch length	32.7	32.3	30.8–34.3	32.1	31.6–33.4	32.5	30.8–34.3	34.3	na

* N=10 for CF, TL ** N=2 for CF, TL *** N=6 for SL, PELVB, PELFS, PELVF, PECFB, PECF2, DFB, DFAe, AFB, AFPe, AdFB, CD, PAdC, DHL, PreP, PreA, PreD, BD, PostD, H; N=5 for PECF1, DFPe, DFPd, CL; N=4 for HL; N=1 for CF, TL, UA, LA.

**Table 3.** Morphological and meristic data of *Coregonusintermundia* from Lake Lucerne, NMBE-1078097 holotype, contemporary specimen; paratypes of contemporary specimen *N* = 13. For males and both sexes the holotype is included in the range and mean.

Species	*Coregonusintermundia*						
Morphological characters	Holotype	Holotype + Paratypes		Paratypes		Holotype + Paratypes	
		Ntotal = 14 *		Nfemales = 3		Nmales = 11 **	
		mean	range	mean	range	mean	range
**Standard length (mm)**	274	262.4	232–278	249.3	237–260	266	232–278
**Percentage of standard length**							
Pelvic fin base	4.1	4	3.4–4.7	3.7	3.4–3.8	4	3.4–4.7
Pelvic fin ‚spine‘ length	6.5	6.5	5.6–7.6	6.7	6.2–7.6	6.4	5.6–7.2
Pelvic fin length	15.4	16	15–17.5	16.1	15.7–16.9	16	15–17.5
Pectoral fin base	3	3.2	2.9–3.6	3.1	2.9–3.1	3.2	3–3.6
Pectoral fin 1 length	15.7	16	15.2–16.9	15.8	15.3–16.7	16	15.2–16.9
Pectoral fin 2 length	16.2	16.7	15.9–18.2	16.9	16.4–17.2	16.6	15.9–18.2
Dorsal fin base	12.4	12.2	10.6–16.2	11.3	11–11.4	12.4	10.6–16.2
Length of anterior part of dorsal fin erected	18.3	18.2	16.7–19.9	18.1	17.6–18.9	18.2	16.7–19.9
Length of anterior part of dorsal fin depressed	19.4	19.7	18.2–21.5	19.8	19.4–20.2	19.6	18.2–21.5
Length of posterior part of dorsal fin erected	4.6	5.6	4.5–6.4	6	5.9–6.1	5.4	4.5–6.4
Anal fin base	12	12.4	10.8–13.8	12.1	11.5–12.5	12.4	10.8–13.8
Length of anterior part of the anal fin	11.9	12.1	11.1–12.8	11.9	11.5–12.2	12.1	11.1–12.8
Adipose fin base	5.1	5.4	4.1–6.7	5.5	5.2–5.9	5.4	4.1–6.7
Caudal fin length	23.7	23.4	21.1–24.9	23.7	23.3–24.4	23.3	21.1–24.9
Caudal peduncle depth	7.4	7.5	7–8.3	7.6	7.3–7.9	7.5	7–8.3
Caudal peduncle length	11.9	12.9	11.6–14.4	12.5	11.9–13.2	13	11.6–14.4
Length from posterior part of adipose fin to caudal fin base	19	18.8	17.5–19.8	18.5	18.2–18.9	18.9	17.5–19.8
Dorsal head length	13.4	14.4	13.4–15.6	14.6	14.1–14.9	14.3	13.4–15.6
Prepelvic length	51.8	50.9	46.5–53.8	53.1	52.6–53.8	50.3	46.5–53.5
Preanal length	78.1	77.3	75.4–80.1	78.4	76.8–80.1	77	75.4–78.5
Predorsal length	44.8	46	42.6–48.4	47	46.1–48.4	45.8	42.6–48.2
Body depth	23.3	23.9	21.6–27.1	24.4	21.7–27.1	23.8	21.6–26
Postdorsal length	43.2	44.5	41.7–47.3	44	41.7–45.6	44.7	42.7–47.3
Head length	20	20.3	19.1–21.5	20.2	19.9–20.4	20.3	19.1–21.5
Total length	119.5	121	117.6–123.1	121.9	121.1–123.1	120.7	117.6–122.3
**Head length (mm)**	54.9	53.3	46.1–58.7	50.4	48.4–52.9	54	46.1–58.7
**Percentage of head length**							
Snout length	23.6	22.9	21.1–24.5	23.3	22.8–24.5	22.7	21.1–24.4
Eye diameter	21.2	22.1	20.4–23.3	22.7	22.4–22.9	21.9	20.4–23.3
Eye cavity	25.7	25.9	23.8–27.9	26.5	26.2–26.8	25.8	23.8–27.9
Eye height	21.8	22.4	20.6–24.7	23.1	22.9–23.5	22.2	20.6–24.7
Eye socket	3.9	4.4	3.4–5.7	4.5	3.6–5.2	4.4	3.4–5.7
Postorbital length	52.3	52.6	50.2–55.8	51.7	51.3–52.3	52.8	50.2–55.8
Head depth	62.9	68.3	62.9–75	69.5	66.9–70.8	68	62.9–75
Mouth width	9	9.5	8.7–10.8	9.8	9.3–10.4	9.4	8.7–10.8
Upper jaw length	29.1	28.1	23.7–30.4	27.3	25–29.9	28.2	23.7–30.4
Lower jaw length	40.4	39.9	38.8–42	39.6	38.8–40.2	40	39.2–42
Length of maxilla	20.6	20.6	18.2–22.3	20.5	19.8–21.2	20.7	18.2–22.3
Snout depth	10	8.3	5.2–10.8	7.9	7.4–8.3	8.4	5.2–10.8
Snout width	14.8	16.1	14.8–18.2	16.2	15.8–16.7	16.1	14.8–18.2
Head width	47	46.4	43.5–49.8	45.3	44.1–46.3	46.7	43.5–49.8
Interorbital width	27.6	27.4	25.4–29.6	27	26.3–27.7	27.5	25.4–29.6
Internarial width	10.2	11.8	10–14.1	12.1	10.6–13.6	11.8	10–14.1
Lower jaw length	7.8	7.6	6.2–9.1	7.4	7.1–7.6	7.6	6.2–9.1
Lower jaw width	22.2	22	20.7–23.8	21.7	21.1–22.7	22.1	20.7–23.8
Middle gill raker length	10	11.9	10–13.6	12.7	11.7–13.3	11.6	10–13.6
Longest gill raker length	10.4	13.1	10.4–16.7	13.7	13.2–14.2	12.9	10.4–16.7
Upper arch length	13	18.4	13–20.6	18.5	17.2–19.7	18.4	13–20.6
Lower arch length	28.3	33.2	28.3–36.3	33	30.1–35.8	33.2	28.3–36.3

*Coregonuslitoralis* – *Coregonussuspensus*

*Coregonuslitoralis* differs from *C.suspensus* by being deeper bodied (23.4–30% SL, mean = 26.9 vs. 21.5–25.1% SL, mean = 23.6), having a longer dorsal fin base (11.9–15.1% SL, mean = 13.4 vs. 10.5–12.1% SL, mean = 11.3), a deeper caudal peduncle (7.5–8.4% SL, mean = 8.1 vs. 7.1–7.5% SL, mean = 7.3), a smaller eye (eye diameter: 18.2–21.4% HL, mean = 20.2 vs. 21.1–22.3% HL, mean = 21.8), a deeper head (70.1–77.9% HL, mean = 73.8 vs. 63.6–70.8% HL, mean = 67.5) and shorter gill rakers (middle gill raker: 7.8–11.8% HL, mean = 10 vs. 11.5–14.3% HL, mean= 13.1; longest gill raker: 9.2–13.1% HL, mean = 11 vs. 13.2–16.4% HL, mean = 14.7) (Tables 2, 4). *Coregonuslitoralis* can be distinguished from *C.suspensus* based on a larger ‘caudal peduncle depth / upper jaw width’ ratio (CD/UJW: 1.74–1.97 vs. 1.5–1.68) and a larger ‘caudal peduncle depth / predorsal length’ ratio (CD/PreD: 0.16–0.18 vs. 0.15–0.16) (Table 10).

**Table 4.** Morphological and meristic data of *Coregonussuspensus* from Lake Lucerne, NMBE-1078100, holotype contemporary specimen; paratypes of contemporary specimens *N* = 4. For males and both sexes the holotype is included in the range and mean.

Species	*Coregonussuspensus*							
Morphological characters	Holotype	Holotype + Paratypes		Paratypes		Holotype + Paratypes		
		Ntotal = 5 *		Nfemales = 2		Nmales = 3		
		mean	range	NMBE-1078082	NMBE-1078081	NMBE-1078099	NMBE-1078100	NMBE-1078101
**Standard length (mm)**	258.5	271.4	255–302	260	301.5	255	259	282
**Percentage of standard length**								
Pelvic fin base	3.8	3.7	3.3–3.8	3.3	3.8	3.8	3.8	3.8
Pelvic fin ‘spine’ length	6.0	5.8	5.4–6.4	5.4	5.4	6.4	6	5.9
Pelvic fin length	16.1	15.3	15–16.1	15	15.2	15.3	16.1	15
Pectoral fin base	3.2	3.1	3–3.2	3	3	3.1	3.2	3
Pectoral fin 1 length	16.4	15.6	14.7–16.8	14.7	16.8	15.6	16.4	14.7
Pectoral fin 2 length	17.1	16.4	15.3–17.2	15.3	17.2	16.9	17.1	15.4
Dorsal fin base	11.5	11.3	10.5–12.1	10.5	11.6	12.1	11.5	10.6
Length of anterior part of dorsal fin erected	17.7	17.6	16.9–17.8	17.8	17.8	17.8	17.7	16.9
Length of anterior part of dorsal fin depressed	19.8	19.1	18.2–19.8	18.7	19.5	19.3	19.8	18.2
Length of posterior part of dorsal fin erected	5.8	5.2	4.8–5.8	5.6	4.8	5	5.8	4.8
Anal fin base	13.1	12.7	11.3–13.1	11.3	13.1	13.1	13.1	12.8
Length of anterior part of the anal fin	12.4	11.9	11.6–12.4	11.6	11.9	11.7	12.4	12.1
Adipose fin base	5.5	5	4.2–5.5	5.3	4.2	4.7	5.5	5.2
Caudal fin length	24.4	23	21.1–24.4	21.1	23.5	22.6	24.4	23.5
Caudal peduncle depth	7.5	7.3	7.1–7.5	7.2	7.1	7.3	7.5	7.2
Caudal peduncle length	11.5	12.4	11.5–13.7	13.7	12	11.9	11.5	12.8
Length from posterior part of adipose fin to caudal fin base	17.8	19	17.8–20.6	20.6	18.3	19	17.8	19.2
Dorsal head length	14.4	13.8	12.8–14.5	13.8	12.8	14.5	14.4	13.4
Prepelvic length	48.8	49.2	47.9–51	51	47.9	48.3	48.8	49.9
Preanal length	77.8	76.5	75.6–77.8	76.7	75.6	75.7	77.8	77
Predorsal length	48.4	47.1	45.6–48.4	46.5	45.6	48.1	48.4	46.9
Body depth	23.2	23.6	21.5–25.1	23.7	25.1	24.3	23.2	21.5
Postdorsal length	44.5	44.3	42.8–46.2	44.8	43	46.2	44.5	42.8
Head length	20.3	19.6	18.7–20.4	19.2	19	20	20	19.4
Total length	122.2	120	118.5–122.2	118.5	118.7	120.6	122.2	119.9
**Head length (mm)**	52.4	53.1	50–56.2	50	56.2	51.9	52.4	54.8
**Percentage of head length**								
Snout length	23.7	23.2	21.9–24	23.4	21.9	24	23.7	23.1
Eye diameter	22.3	21.8	21.2–22.3	22	21.6	21.9	22.3	21.2
Eye cavity	25.6	25.6	25.1–26.4	26.4	25.1	25.4	25.6	25.6
Eye height	22.2	22.2	20.8–23.3	23.3	22.1	22.5	22.2	20.8
Eye socket	2.7	3.8	2.7–5.2	3.7	3.6	4	2.7	5.2
Postorbital length	52.7	53	52.1–54.2	52.1	54.2	53.9	52.7	52.1
Head depth	66.4	67.5	63.6–70.8	70.8	70.4	63.6	66.4	66.3
Mouth width	8.9	9.2	8.9–9.7	9	9.7	9.3	8.9	9.2
Upper jaw length	29.3	28.5	27.1–29.3	28.6	28.5	28.9	29.3	27.1
Lower jaw length	41.6	40.1	37.1–41.6	37.1	40.5	40.8	41.6	40.8
Length of maxilla	18.5	19.9	18.3–21	21	20.9	20.7	18.5	18.3
Snout depth	7.5	8.1	6.5–9.9	7.3	9.5	6.5	7.5	9.9
Snout width	17.6	16.5	15.7–17.6	16.8	15.9	16.6	17.6	15.7
Head width	49.2	48	45.5–50	47.8	50	45.5	49.2	47.4
Interorbital width	27.9	27.5	26.5–28.6	26.5	28.6	26.6	27.9	28
Internarial width	13.6	12.9	12.1–13.6	12.6	13.3	12.1	13.6	13.1
Lower jaw length	7.8	7.5	6.5–8.4	7.7	8.4	6.5	7.8	7.1
Lower jaw width	24.5	23.1	22.1–24.5	22.5	23.3	22.9	24.5	22.1
Middle gill raker length	13.7	13.1	11.5–14.3	11.5	12.9	14.3	13.7	na
Longest gill raker length	15.6	14.7	13.2–16.4	13.7	13.2	16.4	15.6	na
Upper arch length	18	17.8	17.3–18.6	17.4	17.3	18.6	18	na
Lower arch length	35.6	34.7	33.8–35.6	34.9	34.4	33.8	35.6	na

*Coregonuslitoralis* – *Coregonusnobilis*

*Coregonuslitoralis* can be differentiated from *C.nobilis* by being deeper bodied (23.4–30% SL, mean = 26.9 vs. 20.7–25.5% SL, mean = 23.8), having a longer dorsal fin base (11.9–15.1% SL, mean = 13.4 vs. 10.3–12.6% SL, mean = 11.2), a anteriorly longer erected and depressed dorsal fin (erected dorsal fin: 16.9–20.9% SL, mean = 18.9 vs. 15.3–17.2% SL, mean = 16.3; depressed dorsal fin: 17.6–22.6% SL, mean = 20.4 vs. 16.7–18.8% SL, mean = 17.8), a deeper caudal peduncle (7.5–8.4% SL, mean = 8.1 vs. 6.3–7.6% SL, mean = 6.8) and shorter gill rakers (middle gill raker: 7.8–11.8% HL, mean = 10 vs. 10.2–16.6% HL, mean= 13.4; longest gill raker: 9.2–13.1% HL, mean = 11 vs. 10.6–17% HL, mean = 14.2) (Tables 2, 5). Based on ratios *C.litoralis* differs from *C.nobilis* by having a larger ‘caudal peduncle depth / postdorsal length’ ratio (CD/PostD: 0.17–0.21 vs. 0.14–0.16) and a smaller ‘pectoral fin length / length of erected anterior part of dorsal-fin’ ratio (PecF2/DFAe: 0.84–0.92 vs. 0.94–1.1) (Table 10).

**Table 5.** Morphological and meristic data of *Coregonusnobilis* from Lake Lucerne, MHNG-656.056 neotype, historical specimen; syntypes of historical specimens *N* = 2, non-types of contemporary specimens *N* = 21. The neotype is included in the range and mean of the historical specimens.

Species	*Coregonusnobilis*								
Morphological characters	Neotype	Neotype + syntypes		non-types					
		Ntotal = 3 *		Ntotal = 21		Nfemales = 9		Nmales = 12	
		mean	range	mean	range	mean	range	mean	range
**Standard length (mm)**	207	236	207–254	280.4	253–315	276.3	253–313	283.5	264–315
**Percentage of standard length**									
Pelvic fin base	3.9	3.8	3.5–4	3.9	3.2–4.5	3.7	3.2–4.5	4	3.6–4.5
Pelvic fin ‘spine’ length	6.4	6.3	5.9–6.5	6	4–7.2	5.7	4–6.5	6.2	5.2–7.2
Pelvic fin length	17.5	16.3	15.4–17.5	15.2	14.1–16.5	15.2	14.1–16.2	15.2	14.4–16.5
Pectoral fin base	3.7	3.4	3–3.7	3.3	3–3.6	3.3	3–3.5	3.3	3.1–3.6
Pectoral fin 1 length	19.2	18	16.9–19.2	15.8	14.9–16.7	16.1	15.3–16.6	15.6	14.9–16.7
Pectoral fin 2 length	20	18.3	17.2–20	16.6	15.5–17.6	16.8	15.6–17.5	16.4	15.5–17.6
Dorsal fin base	10.2	10.7	10.2–11.2	11.2	10.3–12.6	10.8	10.3–11.2	11.4	10.3–12.6
Length of anterior part of dorsal fin erected	18.3	17.5	16.7–18.3	16.3	15.3–17.2	16.3	15.4–17.1	16.3	15.3–17.2
Length of anterior part of dorsal fin depressed	19.4	18.6	18.1–19.4	17.8	16.7–18.8	17.7	16.7–18.8	17.8	16.9–18.6
Length of posterior part of dorsal fin erected	6.1	5.6	5.3–6.1	5.2	4.7–5.9	5.3	4.9–5.9	5.1	4.7–5.9
Anal fin base	12.3	10.9	9.7–12.3	12.1	10.9–14.2	11.8	10.9–13.3	12.4	11.3–14.2
Length of anterior part of the anal fin	13.1	10.9	9.2–13.1	11.1	10–12.6	11.1	10.1–12.6	11.1	10–11.9
Adipose fin base	6.5	5.5	5–6.5	5.3	4.5–6.5	5.4	4.8–6.5	5.3	4.5–6.3
Caudal fin length	na	na	na	22.3	20.1–23.8	22.1	20.1–23.8	22.5	21.4–23.7
Caudal peduncle depth	7.1	7.2	7.1–7.4	6.8	6.3–7.6	6.8	6.3–7.1	6.9	6.5–7.6
Caudal peduncle length	12.4	13.9	12.4–15.3	13.2	11.6–14.5	13.1	12.3–14.2	13.2	11.6–14.5
Length from posterior part of adipose fin to caudal fin base	20.9	20.5	19.8–20.9	18.8	17–20.5	18.8	17–19.9	18.9	17–20.5
Dorsal head length	16.2	14.8	13.9–16.2	13.6	12.8–14.9	13.4	12.9–14.3	13.7	12.8–14.9
Prepelvic length	52.4	50.3	48.8–52.4	50.5	47.9–52	50.9	48.9–52	50.1	47.9–51.6
Preanal length	76.8	76.8	76.1–77.6	76.5	74.2–78.5	76.9	75.1–78.5	76.3	74.2–77.6
Predorsal length	46.7	46.8	46–47.6	45.8	43.3–48.5	46	43.3–48.5	45.6	43.8–46.7
Body depth	22.4	22.5	22.3–23	23.8	20.7–25.5	24.1	21.7–25.5	23.6	20.7–25.5
Postdorsal length	42.9	45.8	42.9–47.3	44.9	42.6–48.2	44.9	43.5–46.4	44.9	42.6–48.2
Head length	22.4	20.7	19.6–22.4	19.8	18.5–21.9	19.7	18.5–20.6	19.9	18.8–21.9
Total length	na	na	na	119	113.7–122.1	118.7	113.7–122.1	119.2	116.8–121.2
**Head length (mm)**	46.3	48.6	46.3–51	55.5	49.1–59.8	54.3	49.1–57.9	56.3	53.5–59.8
**Percentage of head length**									
Snout length	21.3	20.6	19.9–21.3	23.2	20.8–25.6	22.9	20.8–25.6	23.3	21.2–25.1
Eye diameter	25.6	24.2	23.5–25.6	21.8	20.2–23.1	22.2	21.2–23.1	21.5	20.2–22.7
Eye cavity	31.1	29.1	27.6–31.1	26	24.2–28	26.4	24.8–28	25.8	24.2–27.8
Eye height	25.8	25.9	25.4–26.5	22.3	20.7–24	22.4	21.1–23.2	22.3	20.7–24
Eye socket	4.6	4.7	4.6–5	4.2	3–5.3	4.5	3.4–5.3	3.9	3–5
Postorbital length	49.5	51.6	49.5–53.1	52.6	50.4–55.3	52.7	50.4–54.7	52.5	51.3–55.3
Head depth	73.3	75.2	73.3–76.6	71.2	65.9–77.8	72.3	68.8–77.8	70.5	65.9–74.6
Mouth width	10.8	11	10.8–11.1	9.6	7.9–11.2	9.5	7.9–10.5	9.7	8.7–11.2
Upper jaw length	30.4	29.8	28.5–30.4	29.5	26.3–32	29.8	26.3–32	29.3	27.6–31.4
Lower jaw length	47.9	45.2	43.6–47.9	40	36.6–42.2	40.3	37.7–42.2	39.7	36.6–42
Length of maxilla	23.9	23.6	22.8–24	21	18.3–24.3	21.2	18.3–22.9	20.9	18.9–24.3
Snout depth	8.2	8.8	8.2–9.7	10.5	7.9–12.4	10.2	7.9–11.7	10.8	8.9–12.4
Snout width	16.2	17	16.2–18.3	17.2	15.1–18.8	17.5	15.4–18.2	17	15.1–18.8
Head width	44.8	46.1	44.8–48.2	50.6	46.2–55	50.9	47.1–55	50.4	46.2–52.7
Interorbital width	27.1	28.2	27–30.4	28.4	25.3–31.6	28.5	25.3–31.6	28.3	26.6–31.1
Internarial width	11.8	12.6	11.8–13.7	12.3	10.1–15	12.6	10.8–15	12.1	10.1–13.3
Lower jaw length	10	9	8.3–10	9	7.8–10.2	8.8	8–9.5	9.1	7.8–10.2
Lower jaw width	24.8	24.5	22.3–26.3	24.3	18.7–27.2	24.5	18.7–27.2	24.1	20.6–25.9
Middle gill raker length	14.8	14.8	na	13.4	10.2–16.6	13.1	10.2–16.6	13.6	12.3–15.1
Longest gill raker length	15	15	na	14.2	10.6–17	14.2	10.6–17	14.2	12.6–15.4
Upper arch length	na	na	na	18.7	16.4–20.5	18.9	16.8–20.5	18.6	16.4–19.7
Lower arch length	na	na	na	34.2	31.7–39.3	34.4	31.7–39.3	33.9	32.6–36.4

* N=2 for PecF1 and N=1 for MGR, LGR.

*Coregonuslitoralis* – *Coregonusmuelleri*

Contemporary specimens: *Coregonuslitoralis* differs from *C.muelleri* by having strong pigmentation of all fins vs. none or very little pigmentation of the pectoral, anal, caudal and dorsal fin in *C.muelleri*, by being deeper bodied (23.4–30% SL, mean = 26.9 vs. 19.8–24.9% SL, mean = 21.9), having a longer dorsal fin base (11.9–15.1% SL, mean = 13.4 vs. 9.2–12.7% SL, mean = 11), a shorter adipose fin base (3.8–5.8% SL, mean = 5 vs. 4.6–8.5% SL, mean = 6), a shorter head (19.2–20.9% SL, mean = 19.9 vs. 20.3–23.5% SL, mean = 22), a smaller eye (eye diameter: 18.2–21.4% HL, mean = 20.2 vs. 22.2–26% HL, mean = 24.1; eye cavity: 22.7–26% HL, mean = 24.6 vs. 26.4–31.1% HL, mean = 27.7), eye socket shape (sickle cell-shaped vs. roundish), deeper head (70.1–77.9% HL, mean = 73.8 vs. 59.6–70.8% HL, mean = 63.7), a shorter lower jaw (36.8–42.5% HL, mean = 39.6 vs. 40.3–45.8% HL, mean = 42.7), a shorter maxilla (17.4–20.5% HL, mean = 19.1 vs. 19.4–24% HL, mean = 21.4), a deeper snout (6.9–10.5% HL, mean = 8.6 vs. 4.9–8.7% HL, mean = 7), a wider head (46.4–51.2% HL, mean = 49.1 vs. 37.2–48.7% HL, mean = 43.8) and shorter gill rakers (middle gill raker: 7.8–11.8% HL, mean = 10 vs. 9.6–15.9% HL, mean = 13.5; longest gill raker: 9.2–13.1% HL, mean = 11 vs. 12.5–16.7% HL, mean = 14.5) (Tables 2, 6). Based on ratios *C.litoralis* can be distinguished from *C.muelleri* by having a larger ‘caudal peduncle depth / eye diameter’ ratio (CD/ED: 1.87–2.25 vs. 1.12–1.43) and a larger ‘head depth / snout width’ ratio (HD/SW: 4.45–5.55 vs. 3.45–4.45) (Table 10).

**Table 6.** Morphological and meristic data of *Coregonusmuelleri* from Lake Lucerne, NMBE-1078123 holotype, contemporary specimen; paratypes of contemporary specimens *N* = 29 and paratype material of historical specimens *N* = 8. For males and both sexes the holotype is included in the range and mean of the contemporary specimens.

Species	*Coregonusmuelleri*								
Morphological characters		Contemporary specimens						Historical specimens	
	Holotype	Holotype + Paratypes		Paratypes		Holotype + Paratypes		Paratypes	
		Ntotal = 30 *		Nfemales = 9 **		Nmales = 21 ***		Ntotal= 8 ****	
		mean	range	mean	range	mean	range	mean	range
**Standard length (mm)**	180	180.7	158–198	180.1	161–197	181	158–198	180.2	159–193
**Percentage of standard length**									
Pelvic fin base	3.6	3.6	3–4.1	3.7	3.3–4.1	3.6	3–3.9	3.5	3.2–4
Pelvic fin ‘spine’ length	7.2	6.7	5.3–8.1	6.8	5.3–8.1	6.6	5.5–7.2	5.9	4.8–6.6
Pelvic fin length	15.6	15.9	14.6–16.8	16.2	15–16.6	15.7	14.6–16.8	16.4	14.2–18.9
Pectoral fin base	3.4	3	2.7–3.5	3	2.7–3.3	3.1	2.8–3.5	3	2.4–3.6
Pectoral fin 1 length	16.8	16.4	15–18	16.7	15.8–18	16.2	15–16.9	16.7	15.3–18.8
Pectoral fin 2 length	17.8	17.1	16–18.8	17.3	16.6–18.8	17.1	16–17.9	17.5	15.9–20
Dorsal fin base	10.8	11	9.2–12.7	11.2	10.1–12	11	9.2–12.7	10.6	9.3–11.6
Length of anterior part of dorsal fin erected	18.6	18.1	16.4–20.3	18.8	17.7–20.3	17.8	16.4–19.8	18.4	17.1–20.2
Length of anterior part of dorsal fin depress	e19.6	19.2	17.3–21.1	19.7	18.7–21.1	19	17.3–20.7	19.5	17.9–21.1
Length of posterior part of dorsal fin erected	6.1	5.6	4.5–6.5	5.5	4.5–6.1	5.6	4.9–6.5	6.2	5.4–7.1
Anal fin base	13.8	13	11.2–14.8	13.1	11.6–13.7	12.9	11.2–14.8	12	11.2–13
Length of anterior part of the anal fin	12.4	12	10.5–13	12.3	11.2–13	11.8	10.5–12.7	12	10.9–13.3
Adipose fin base	5.8	6	4.6–8.5	5.9	5–7.2	6.1	4.6–8.5	6.7	5.3–8.6
Caudal fin length	23.9	23.9	22–26.4	24.7	23.1–26.4	23.6	22–25.6	23.5	21–25.4
Caudal peduncle depth	7.2	6.9	6.3–7.4	6.9	6.4–7.4	6.9	6.3–7.3	6.5	6.1–6.8
Caudal peduncle length	13.3	12.8	10.8–14.3	12.7	10.8–14.2	12.8	10.9–14.3	13.8	13.4–15.1
Length from posterior part of adipose fin to caudal fin base	19.2	19.4	17.5–21.1	19.1	17.5–20.6	19.5	18–21.1	20.3	17.7–21.6
Dorsal head length	15.5	15.7	14.4–17	15.8	14.8–17	15.7	14.4–16.4	16	14.8–17.9
Prepelvic length	51.1	51.3	48.2–54.4	52	50.6–54.4	51	48.2–53.3	52.4	49.4–55.3
Preanal length	76.1	76.9	74–80.1	76.4	74–77.4	77.1	74.7–80.1	76.6	75.7–79.4
Predorsal length	47.6	46.7	41.9–49.6	46.1	41.9–48.2	46.9	44.7–49.6	47.3	44.8–51.5
Body depth	22	21.9	19.8–24.9	23.3	21–24.9	21.3	19.8–24	22.5	20–24.7
Postdorsal length	44.8	44.3	41.6–47.5	43.9	41.6–45.6	44.5	42.5–47.5	43.6	39.6–45.8
Head length	21.6	22	20.3–23.5	22.3	21.3–23.5	21.9	20.3–23.3	22.2	20.9–25.1
Total length	123.3	122.1	119.1–124.8	122.2	119.8–124.8	122	119.1–124.7	121.1	119.1–123.6
**Head length (mm)**	38.9	39.8	36.3–44.2	40.1	37.4–44.2	39.6	36.3–42.1	39.9	36.7–46.7
**Percentage of head length**									
Snout length	22	22.8	20.1–25.3	22.6	20.8–23.7	22.9	20.1–25.3	21.3	20–22.6
Eye diameter	26	24.1	22.2–26	24	22.2–25.6	24.2	22.7–26	24.3	23.2–26.6
Eye cavity	28.8	27.7	26.4–31.1	27.8	26.5–31.1	27.6	26.4–29.3	29.2	27–31.9
Eye height	26.1	24.3	22.3–26.2	24.2	22.8–25.1	24.3	22.3–26.2	25.3	23.5–28.3
Eye socket	2.8	3	1.9–4.6	2.9	1.9–3.7	3	2.3–4.6	4	2.8–5.7
Postorbital length	49.9	50.6	48.8–52.6	50.8	49.8–52.2	50.5	48.8–52.6	49.9	47.4–51.9
Head depth	64.9	63.7	59.6–70.8	63.5	61.2–65.7	63.8	59.6–70.8	65.2	61.8–69.7
Mouth width	9.3	9.2	7.6–10.1	9.1	8.5–9.9	9.2	7.6–10.1	10.2	8.9–11.8
Upper jaw length	30	28.8	20.7–31.5	29.6	28.4–31.3	28.4	20.7–31.5	31	28.4–35.9
Lower jaw length	41.4	42.7	40.3–45.8	43.6	41.4–45.8	42.3	40.3–44.9	43.6	39.5–47
Length of maxilla	21.7	21.4	19.4–24	22.1	20.9–23.7	21.2	19.4–24	22.9	21.3–26.2
Snout depth	8.2	7	4.9–8.7	7.1	5.6–8.7	7	4.9–8.3	7.7	7–8.5
Snout width	17.8	16.9	14.2–19.2	16.6	14.2–17.9	17	15.4–19.2	16.5	14.3–17.4
Head width	45	43.8	37.2–48.7	43.8	41.9–47.1	43.7	37.2–48.7	44.5	41.2–47.8
Interorbital width	26.7	26	23.8–29.2	26	24.1–27.2	26.1	23.8–29.2	24.6	22.6–26
Internarial width	10	11	9.6–12.3	11.3	10–12.3	10.9	9.6–12	12.2	11.3–13.8
Lower jaw length	7.6	7.3	5.9–8.7	7.1	6.4–7.9	7.4	5.9–8.7	8.6	7.5–10
Lower jaw width	22.4	21.3	18.5–23.3	21.4	19.2–23	21.2	18.5–23.3	22.8	21–24.7
Middle gill raker length	13.5	13.5	9.6–15.9	13.4	12.4–14	13.6	9.6–15.9	12.8	11.2–15
Longest gill raker length	15.1	14.5	12.5–16.7	14.4	13.5–15.9	14.5	12.5–16.7	14.1	11.2–15.6
Upper arch length	21.6	19.1	15.9–22.3	19.4	16–22	19	15.9–22.3	na	na
Lower arch length	33.6	33.4	26.2–38.7	33	27.2–37.5	33.5	26.2–38.7	na	na

* N=27 for MGR, LGR, UA, LA; ** N=7 for MGR, LGR, UA, LA; *** N=20 for MGR, LGR, UA, LA; **** N=6 for CF, TL; N=5 for MGR, LGR.

Historical specimens: *Coregonuslitoralis* differs from *C.muelleri* by being deeper bodied (21.8–29.9% SL, mean = 26.2 vs. 20–24.7% SL, mean = 22.5), having a deeper caudal peduncle (6.9–8.5% SL, mean = 7.7 vs. 6.1–6.8% SL, mean = 6.5), smaller eye (eye diameter: 17.9–22.7% HL, mean = 20.4 vs. 23.2–26.6% HL, mean = 24.3; eye cavity: 24.1–27.7% HL, mean = 26 vs. 27–31.9% HL, mean = 29.2; eye height: 20.3–24.8% HL, mean = 22.2 vs. 23.5–28.3% HL, mean = 25.3), longer postorbital length (51.6–56.2% HL, mean = 53.5 vs. 47.4–51.9% HL, mean = 49.9), a deeper head (68.1–77.1% HL, mean = 72.5 vs. 61.8–69.7% HL, mean = 65.2), more transverse dorsal scales (10–11, mode = 10 vs. 8–9, mode = 9) and less gill rakers (upper arch gill raker number: 7–11 vs. 13–16; lower arch gill raker number: 12–22 vs. 22–25; total gill raker number: 19–33 vs. 36–39) (Tables 2, 6, 12, 13). The contemporary gill raker range of *C.litoralis* (24 to 32, modes = 26 and 30) overlaps mostly with the historical gill raker ranges given in Fatio (1890: 23–28), Birrer and Schweizer (1938: 23–30, mode = 26) and Steinmann (1950: 20–31) and counted on historical specimens (this study: 19–33). Based on ratios *C.litoralis* can be distinguished from *C.muelleri* by having a larger ‘caudal peduncle depth / dorsal head length’ ratio (CD/DHL: 0.46–0.58 vs. 0.37–0.44) (Table 11).

###### Distribution and notes on biology.

**Figure 2.**
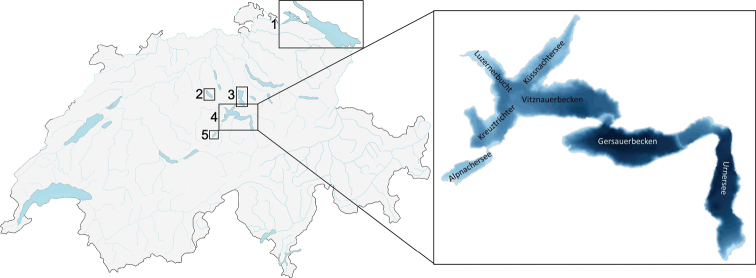
Map of Switzerland with the lakes studied; 1. Lake Constance, 2. Lake Sempach, 3. Lake Zug, 4. Lake Lucerne, 5. Lake Sarnen. On the right is a bathymetric map of Lake Lucerne with the German names of each basin (modified from Alexander et al. 2017a).

*Coregonuslitoralis* occurs in all basins of Lake Lucerne (Fig. 2). Based on genetic assignments it has been identified to be present in Lake Sarnen (Suppl. material 1: figs S2, S3). It is unclear if *C.litoralis* occurs naturally in Lake Sarnen or has been introduced (this is discussed in more detail in the Suppl. material 1: paragraph 1). *Coregonuslitoralis* feeds predominantly on benthic prey (e.g., chironimid, pisidium) and parts of the year on zooplankton (e.g., bythotrephes, daphnia) (stomach content: Cysat 1661; Birrer and Schweizer 1938; isotopic signature: Selz 2008; Hudson 2011; Ingram et al. 2012). Feeding experiments with individuals of this species and other whitefish species from lakes Thun and Lucerne (Lundsgaard-Hansen et al. 2013; Roesch et al. 2013) suggest – based on the functional properties of the number of gill rakers – that the low-rakered *C.litoralis* feeds predominantly on benthic prey. *Coregonuslitoralis* has a fast growth rate (Svarvar and Müller 1982; Müller et al. 2007) and is a large whitefish species. The size (i.e., standard length) at 3 years of age of *C.litoralis* is larger than that of *C.suspensus* and *C.intermundia* and considerably larger than that of *C.muelleri* (Suppl. material 1: table S3). We cannot compare its size to *C.nobilis* since only older individuals of *C.nobilis* have been caught (Hudson et al. 2016). *Coregonuslitoralis* has a short spawning season in winter. The peak spawning period varies over the years from mid-November to late December, which has been attributed to a decrease in lake water temperature below 7–8 °C (per. comm. from fisheries authorities of Lake Lucerne; Birrer and Schweizer 1938). The spawning season lasts for approximately two weeks and the spawning depth is mostly in very shallow waters (1–5 m) but can reach down to 30 m and rarely to 40 m in the main basins of Lake Lucerne excluding Lake Alpnach (Birrer and Schweizer 1938; Steinmann 1950; Hudson et al. 2016). In all the basins of Lake Lucerne except Lake Alpnach the spawning depth of *C.litoralis* overlaps with that of *C.intermundia* (Hudson et al. 2016). Populations of *C.litoralis* in Lake Alpnach usually spawn earlier in the year than in the rest of Lake Lucerne, which has been suggested to be due to a more rapid cooling of the lake water in Lake Alpnach than in the rest of the basins of Lake Lucerne (Svarvar and Müller 1982; A. von Deschwanden and A. Blättler, pers. comm.). *Coregonuslitoralis* mostly spawns though next to the entrance of Lake Alpnach in the adjacent basin ‘Kreuztrichter’ and less so directly in Lake Alpnach (this is discussed in more detail in the Suppl. material 1: paragraph 2). The spawning behaviour of *C.litoralis* has been recorded by Selz and Hofmann (2018). The video by Selz and Hofmann (2018) shows communal pair-spawning behaviour whereby a female is often accompanied by up to three males. Towards the end of the video a pair spawning event can be observed in which the female and male align side by side and synchronically dart from near the bottom up towards the surface. This is the first observation of this behaviour in pre-alpine whitefish species; it had previously only been observed in the vendace (*Coregonusalbula*) in an experimental setting (Karjalainen and Marjomäki 2017).

###### Etymology.

The specific name in Latin means ‘coming from the shore’. The name refers to the specific spawning habitat of this species, spawning in very shallow water of a few metres close to or directly at the lake shore. An adjective.

**Figure 3.**
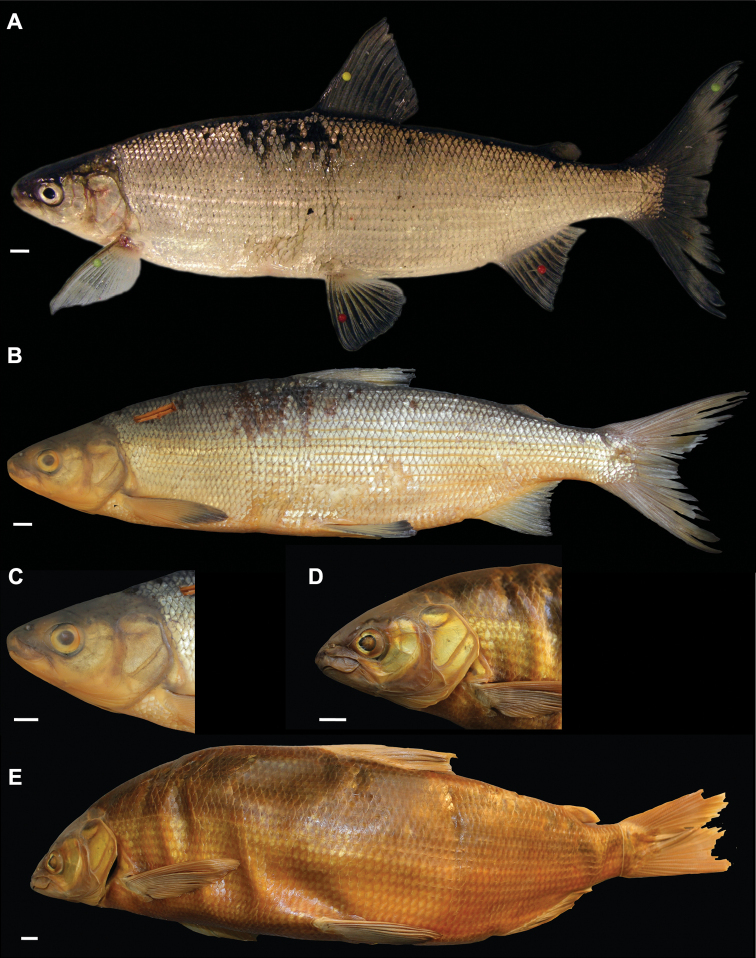
*Coregonuslitoralis*, Lake Lucerne, Switzerland. Contemporary (**A–C**) and historical (**D, E**) specimens **A** holotype, NMBE-1078103, 326 mm SL, male, freshly caught specimen (right side, reversed) **B, C** holotype, NMBE-1078103, preserved specimen **D, E** paratype, MHNG-717.046, 361.5 mm SL. Scale bars: 1 cm.

###### Common names.

Balchen, Bodenbalchen.

##### ﻿ Coregonus intermundia sp. nov.

Taxon classificationAnimaliaSalmoniformesCoregonidae

474FED52-B33F-5B4F-A441-8E8870B6AE80

https://zoobank.org/DC385078-9965-4DF9-AB15-031FF2D2516E

**Figure 4.**
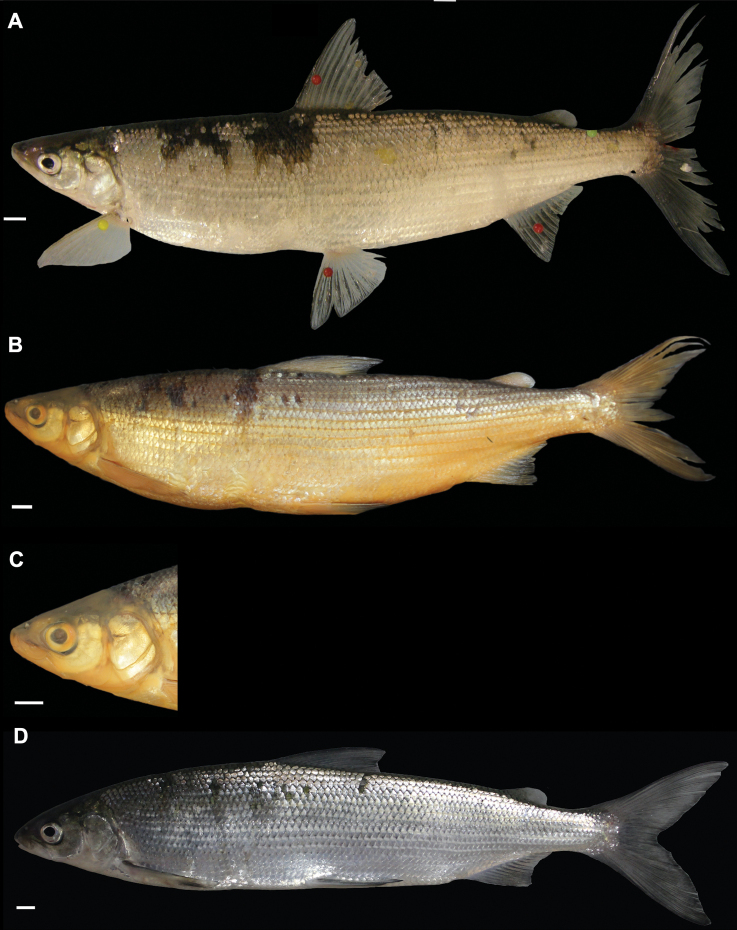
*Coregonusintermundia*, Lake Lucerne, Switzerland **A** holotype, NMBE-1078097, 274 mm SL, male, freshly caught specimen, (right side, reversed) **B, C** holotype, NMBE-1078097, preserved specimen **D** non-type, Eawag-124308, Lake Lucerne, 316 mm SL, male. Scale bars: 1 cm.

Figs 4 , 14 , Tables 3 , 10 , 12 , 13 , 14 , Suppl. material 1: figs S2, S3, tables S1–S3

Coregonus sp. ‘benthic intermediate’: Hudson et al. 2016.
Coregonus sp. ‘Schwebbalchen’: Vonlanthen et al. 2012; Alexander et al. 2017a (see also synonymy of C.suspensus).

###### Material examined.

***Holotype*.** Contemporary specimen (year: 2007): NMBE-1078097, 274 mm SL, male; Switzerland: Lake Lucerne. ***Paratypes*.** All from Switzerland, Lake Lucerne: Contemporary specimens (year: 2007): NMBE-1078076, NMBE-1078077, NMBE-1078078, NMBE-1078084, NMBE-1078086-1078090, NMBE-1078093-1078096, *N* =13, 232–278 mm SL.

###### Diagnosis.

*Coregonusintermundia* is a medium-sized whitefish (standard length at 3 years of age: range = 243–300 mm, mean = 273) with moderate pigmentation of all fins and the body; greenish blue colour on the flanks above the lateral line; moderate number of pigmented small dots on the scales along the flank; short head (19.1–21.5% SL, mean = 20.3); sub-terminal mouth; thick (3.4–5.7% HL, mean = 4.4) and triangular-shaped eye socket; rather short and stout caudal peduncle (caudal peduncle depth: 7–8.3% SL, mean = 7.5; caudal peduncle length: 11.6–14.4% SL, mean = 12.9).

###### Description.

***Shape***: Only slightly deep bodied with greatest body depth anterior of dorsal fin. Dorsal profile from tip of snout to anterior origin of dorsal fin and ventral profile from interorbital area to pelvic fin origin are moderately convex. Head moderately short. Mouth moderately long and mostly sub-terminal and rarely terminal. Lower jaw moderately wide. Rostral plate equally wide as deep, not strongly pronounced with tip of the snout often more pointed than blunt. Eye-socket thick and sickle cell-shaped. Pectoral fin moderately tapered and moderately short. Dorsal fin rather short with anterior unbranched ray of erected dorsal fin mostly 70–80° angle to body axis, almost straight and only very slightly bent posteriorly at end of the ray. Dorsal fin longest anteriorly and progressively shortening posteriorly with outer margin of dorsal fin mostly straight and rarely concave. Caudal peduncle stout and moderately short with caudal fin moderately forked. Unbranched rays of anal fin slightly bent posteriorly. Anal fin longest anteriorly and progressively shortening posteriorly with outer margin of anal fin mostly straight and only rarely slightly concave. ***Meristics***: Moderately many and moderately long gill rakers. ***Colour***: Pigmentation of fins and body overall moderately strong in live specimens. Pectoral fin slightly pigmented at distal parts of fin. Pelvic fin moderately pigmented at the median to distal parts of fin. All other fins strongly pigmented. Silvery appearance along flanks with moderate number of pigmented small dots (aggregation of melanophores) on scales along flank and dorsum. Distribution of dots bound to scale patterning such that dots are found at edge of the scales or at boundary point of two scales. Dorsally above the lateral line the silvery appearance changes to a pale greenish to dark greenish blue colour and sometimes with pale mustard yellow ground colour. Dorsal part of head and snout around nostrils moderately pigmented. Pre-operculum and operculum silvery with one black spot on lower margin of pre-operculum. Preserved specimens pale in colouration with similar pigmentation as described for live specimens. The silvery, translucent, not coloured or unpigmented parts of body become yellowish brown, whereas pigmented parts are conserved and coloured parts (dorsally above the lateral line) become brownish.

###### Differential diagnosis.

The differential diagnosis against *C.litoralis* and *C.sarnensis* are given under those species’ accounts.

*Coregonusintermundia* – *Coregonussuspensus*

*Coregonusintermundia* can be distinguished from *C.suspensus* based on having a smaller ‘predorsal length / eye diameter’ ratio (PreD/ED: 9.92–10.59 vs. 10.73–11.4) (Table 10).

*Coregonusintermundia* – *Coregonusnobilis*

*Coregonusintermundia* differs from *C.nobilis* by having an anteriorly longer erected and depressed dorsal fin (erected dorsal fin: 16.7–19.9% SL, mean = 18.2 vs. 15.3–17.2% SL, mean = 16.3; depressed dorsal fin: 18.2–21.5% SL, mean = 19.7 vs. 16.7–18.8% SL, mean = 17.8), a shallower snout (5.2–10.8% HL, mean = 8.3 vs. 7.9–12.4% HL, mean = 10.5) and head (43.5–49.8% HL, mean = 46.4 vs. 46.2–55% HL, mean = 50.6 (Tables 3, 5). Based on ratios *C.intermundia* differs from *C.nobilis* by having a larger ‘caudal peduncle depth / head width’ ratio (CD/HW: 0.77–0.87 vs. 0.63–0.77) (Table 10).

*Coregonusintermundia* – *Coregonusmuelleri*

*Coregonusintermundia* differs from *C.muelleri* by having strong pigmentation of all fins vs. none or very little pigmentation of the pectoral, anal, caudal and dorsal fin in *C.muelleri*, by having a deeper caudal peduncle (7–8.3% SL, mean = 7.5 vs. 6.3–7.4% SL, mean = 6.9), a shorter head (19.1–21.5% SL, mean = 20.3 vs. 20.3–23.5% SL, mean = 22), smaller eye (eye diameter: 20.4–23.3% HL, mean = 22.1 vs. 22.2–26% HL, mean = 24.1; eye cavity: 23.8–27.9% HL, mean = 25.9 vs. 26.4–31.1% HL, mean = 27.7), differently shaped eye socket (sickle cell-shaped vs. roundish), a longer postorbital length (50.2–55.8% HL, mean = 52.6 vs. 48.4–52.6% HL, mean = 50.6) and a shorter lower jaw (38.8–42% HL, mean = 39.9 vs. 40.3–45.8% HL, mean = 42.7) (Tables 3, 6). Based on ratios *C.intermundia* differs from *C.muelleri* by having a larger ‘caudal peduncle depth / eye diameter’ ratio (CD/ED: 1.53–1.84 vs. 1.12–1.43) (Table 10). Furthermore, the average standard length at 3 years of age differentiates *C.intermundia* (range = 243–300 mm, mean = 273) from *C.muelleri* (160–232 mm, 194 mm). (Suppl. material 1: table S3).

###### Distribution and notes on biology.

*Coregonusintermundia* occurs in all basins of Lake Lucerne (Fig. 2) except possibly in Lake Alpnach. It has been caught during the spawning season in all basins except for Lake Alpnach, where no nets were set by Hudson et al. (2016). There is no isotopic or stomach content data available for *C.intermundia* to infer the prey spectrum of this species. *Coregonusintermundia* is a medium-sized whitefish species and the size (i.e., standard length) at 3 years of age of *C.intermundia* is smaller than that of *C.litoralis*, considerably larger than that of *C.muelleri* and almost equal to that of *C.suspensus* (Suppl. material 1: table S3). We cannot compare its size to *C.nobilis* since only older individuals of the latter species have been caught (Hudson et al. 2016). *Coregonusintermundia* has most likely a short spawning season in winter around the month of December. The spawning season lasts for approximately two weeks and the spawning depth is mostly in shallow waters (10–30 m, rarely 40 m; Hudson et al. 2016). The spawning depth of *C.intermundia* overlaps partially with that of *C.litoralis*, with the former spawning deeper than the latter (Hudson et al. 2016).

###### Etymology.

The specific name intermundia means in Latin ‘spaces between the worlds’. It refers to the observation that this species is intermediate to *C.litoralis* and *C.suspensus* in its ecology and in some morphological characters. A noun in apposition.

###### Common name.

This species was not recognised by local fisheries, fisheries authorities, researchers, or the public before the works of Lundsgaard-Hansen (2009) and Hudson et al. (2016), who named the species as *Coregonus* sp. ‘benthic-intermediate’ (In German: ‘benthischer Schwebbalchen’).

##### ﻿ Coregonus suspensus sp. nov.

Taxon classificationAnimaliaSalmoniformesCoregonidae

042C18DB-D996-5D16-82E1-9B24F429D354

https://zoobank.org/6063EEA5-E663-484D-A61F-6B349E18F81F

**Figure 5.**
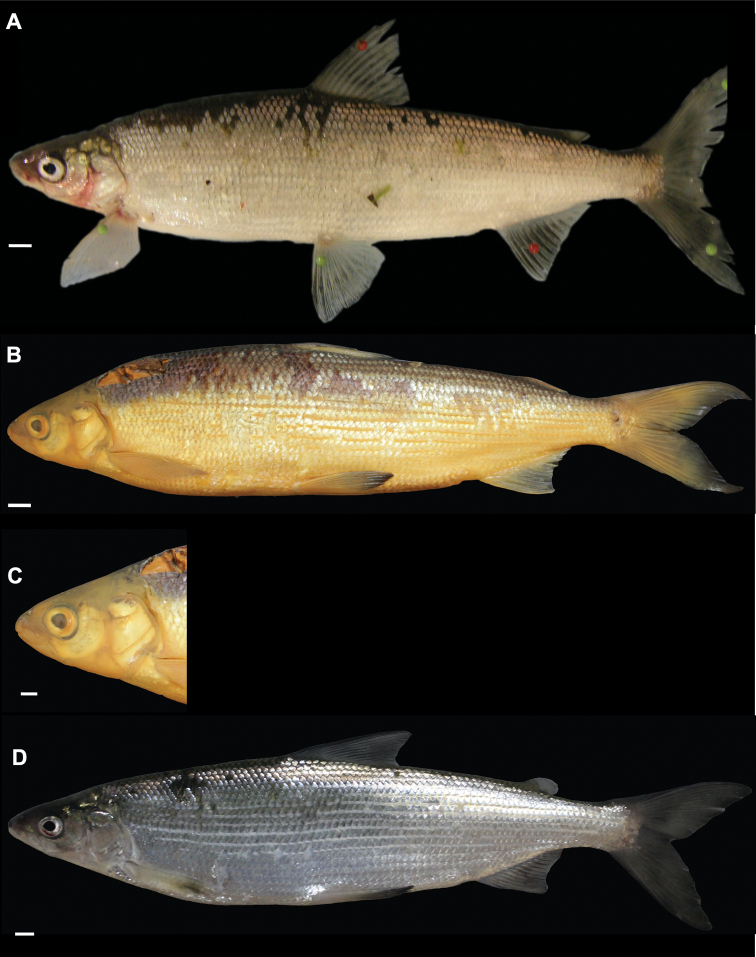
*Coregonussuspensus*, Lake Lucerne, Switzerland **A** holotype, NMBE-1078100, 258 mm SL, male, freshly caught specimen, (right side, reversed) **B, C** holotype, NMBE-1078100, preserved specimen **D** non-type, Eawag-124305, Lake Lucerne, 300 mm SL, male. Scale bars: 1 cm.

Figs 5 , 14 , Tables 4 , 10 , 12 , 13 , 14 , Suppl. material 1: figs S2, S3, tables S1–S3

Coregonus sp. ‘pelagic intermediate’: Hudson et al. 2016.
Coregonus sp. ‘Schwebbalchen’: Vonlanthen et al. 2012; Alexander et al. 2017a (see also synonymy of C.intermundia).

###### Material examined.

***Holotype*.** Contemporary specimen (year: 2007): NMBE-1078100, 258.5 mm SL, male; Switzerland: Lake Lucerne. ***Paratypes*.** All from Switzerland, Lake Lucerne: Contemporary specimens (years: 2007): NMBE-1078081, NMBE-1078082, NMBE-1078099, NMBE-1078101, *N* = 4, 255–301.5 mm SL.

###### Diagnosis.

*Coregonussuspensus* is a medium-sized whitefish (standard length at 3 years of age: range = 266–315 mm, mean = 289 mm) with weak pigmentation of the pectoral fin and moderate pigmentation of all other fins and body; greenish blue colour on the flanks above the lateral line; none to a few pigmented small dots on the scales on the flanks; tip of the snout pointy; triangular eye socket; many and moderately long gill rakers (longest gill raker: 13.2–16.4% HL; total gill rakers number = 33–37).

###### Description.

***Shape***: Only slightly deep bodied with greatest body depth anterior of dorsal fin. Dorsal profile from the tip of snout to anterior origin of dorsal fin and ventral profile from interorbital area to pelvic fin origin is straight and only rarely is dorsal profile from tip of snout to interorbital area slightly convex. Head moderately short. Mouth moderately long and subtly sub-terminal. Lower jaw moderately wide. Rostral plate equally wide as deep, not strongly pronounced with tip of snout often more pointed than blunt. Eye-socket moderately thick and sickle cell-shaped. Pectoral fin moderately tapered and moderately short. Dorsal fin rather short with anterior unbranched ray of erected dorsal fin mostly 60–70° angle to body axis and slightly bent posteriorly. Dorsal fin longest anteriorly and progressively shortening posteriorly with outer margin of dorsal fin mostly concave and rarely straight. Caudal peduncle moderately stout with caudal fin moderately forked. Unbranched rays of anal fin moderately bent posteriorly. Anal fin is longest anteriorly and progressively shortening posteriorly with outer margin of anal fin mostly concave and only rarely straight. ***Meristics***: Many and long gill rakers. ***Colour***: Pigmentation of fins and body overall moderately strong in live specimens. Pectoral fin slightly pigmented at distal parts of fin. Pelvic fin moderately pigmented at median to distal parts of fin. All other fins strongly pigmented. Silvery appearance along flanks with few pigmented small dots (aggregation of melanophores) on scales along flank and dorsum. Distribution of dots bound to scale patterning such that dots are found at edge of scales or at boundary point of two scales. Dorsally above the lateral line silvery appearance changes to a pale greenish or dark greenish blue colour. Dorsal part of head and snout around nostrils moderately pigmented. Pre-operculum and operculum are silvery with one black spot on lower margin of pre-operculum. Preserved specimens pale in colouration with similar pigmentation as described for live specimens. Silvery, translucent, not coloured or unpigmented parts of the body brown-yellowish, whereas pigmented parts conserved and coloured parts (dorsally above lateral line) brownish.

###### Differential diagnosis.

The differential diagnoses against *C.litoralis*, *C.intermundia*, and *C.sarnensis* are given under those species’ accounts. *Coregonussuspensus* shows genetic ancestry contributions from whitefish of Lake Constance, besides its Lake Lucerne ancestry (Douglas and Brunner 2002; Lundsgaard-Hansen 2009; Hudson et al. 2016; De-Kayne et al. 2022; this study). These seem to derive from historically documented introductions of fry of whitefish species from Lake Constance into Lake Lucerne (Svarvar and Müller 1982). Due to the uncertainty of the species origin of the translocated fry and the possibility that there may have been more historically undocumented introductions of other whitefish from Lake Constance we compare the characters of this species with those of all whitefish species from Lake Constance and all other whitefish species in Lake Lucerne.

*Coregonussuspensus* – *Coregonusnobilis*

*Coregonussuspensus* differs from *C.nobilis* by having an anteriorly longer depressed dorsal fin (18.2–19.8% SL, mean = 19.1 vs. 16.7–18.8% SL, mean = 17.8), a shallower head (63.6–70.8% HL, mean = 67.5 vs. 65.9–77.8% HL, mean = 71.2) and snout (6.5–9.9% HL, mean = 8.1 vs. 7.9–12.4% HL, mean = 10.5) (Tables 4, 5). Based on ratios *Coregonussuspensus* can be distinguished from *Coregonusnobilis* by having a smaller ‘pectoral fin base / caudal peduncle depth’ ratio (PecFB/CD: 0.41–0.43 vs. 0.45–0.52) (Table 10).

*Coregonussuspensus* – *Coregonusmuelleri*

*Coregonussuspensus* differs from *C.muelleri* by having strong pigmentation of all fins vs. none or very little pigmentation of the pectoral, anal, caudal and dorsal fin in *C.muelleri*, a deeper caudal peduncle (7.1–7.5% SL, mean = 7.3 vs. 6.3–7.4% SL, mean = 6.9), a shorter head (dorsal head length: 12.8–14.5% SL, mean = 13.8 vs. 14.4–17% SL, mean = 15.7; head length: 18.7–20.4% SL, mean = 19.6 vs. 20.3–23.5% SL, mean = 22), smaller eye (eye diameter: 21.2–22.3% HL, mean = 21.8 vs. 22.2–26% HL, mean = 24.1; eye cavity: 25.1–26.4% HL, mean = 25.6 vs. 26.4–31.1% HL, mean = 27.7; eye height: 20.8–23.3% HL, mean = 22.2 vs. 22.3–26.2% HL, mean = 24.3), differently shaped eye socket (sickle cell-shaped vs. roundish), a longer postorbital length (52.1–54.2% HL, mean = 53 vs. 48.8–52.6% HL, mean = 50.6), a shorter lower jaw (37.1–41.6% HL, mean = 40.1 vs. 40.3–45.8% HL, mean 42.7), a wider head (45.5–50% HL, mean = 48 vs. 37.2–48.7% HL, mean = 43.8) and a wider internarial width (12.1–13.6% HL, mean = 12.9 vs. 9.6–12.3% HL, mean = 11) (Tables 4, 6). *Coregonussuspensus* can be distinguished from *C.muelleri* by having a larger ‘predorsal length / eye diameter’ ratio (PreD/ED: 10.73–11.4 vs. 8.17–9.63) and a larger ‘body depth / lower jaw’ ratio (BD/LJ: 2.71–3.33 vs. 2.01–2.63) (Table 10). Also, the average standard length at 3 years of age differentiates *C.suspensus* (range = 266–315 mm, mean = 289) from *C.muelleri* (160–232 mm, 194 mm) (Suppl. material 1: table S3).

**Lake Constance comparison**

*Coregonussuspensus* – all four Lake Constance species

The shorter dorsal head length of *Coregonussuspensus* (12.8–14.5% SL, mean = 13.8) differentiates it from all species of Lake Constance, *C.wartmanni* (14.5% SL), *C.macrophthalmus* (14.4–16.5% SL, mean = 15.7), *C.gutturosus* (15.4–18.1% SL, mean = 16.8) and *C.arenicolus* (14.8–15.3% SL, mean = 15.1).

*Coregonussuspensus* – *Coregonuswartmanni*

*Coregonussuspensus* differs from *C.wartmanni* by having a longer adipose fin base (4.2–5.5% SL, mean = 5 vs. 4% SL), a larger eye (eye diameter: 21.2–22.3% HL, mean = 21.8 vs. 18.9% HL; eye cavity: 25.1–26.4% HL, mean = 25.6 vs. 23.9% HL; eye height: 20.8–23.3% HL, mean = 22.2 vs. 19% HL), a shorter lower jaw (37.1–41.6% HL, mean = 40.1 vs. 43.5% HL), a shorter maxilla (18.3–21% HL, mean 19.9 vs. 22% HL), longer gill rakers (middle gill raker: 11.5–14.3% HL, mean = 13.1 vs. 10.8% HL; longest gill raker: 13.2–16.4% HL, mean = 14.7 vs. 11.3% HL) and fewer branched pelvic fin rays (10–11, mode = 10 vs. 12) (Tables 4, 9, 14).

*Coregonussuspensus* – *Coregonusmacrophthalmus*

*Coregonussuspensus* can be differentiated from *C.macrophthalmus* by having a smaller eye (eye diameter: 21.2–22.3% HL, mean = 21.8 vs. 21.3–26.1% HL, mean = 24.1; eye cavity: 25.1–26.4% HL, mean = 25.6 vs. 25.4–30.8% HL, mean = 28.9), a shorter lower jaw (37.1–41.6% HL, mean = 40.1 vs. 40–44.4% HL, mean = 42.2), a shorter maxilla (18.3–21% HL, mean 19.9 vs. 20.1–24.7% HL, mean = 23.1), a wider head (45.5–50% HL, men = 48 vs. 39.3–43.3% HL, mean = 41.6) and more lateral line scales (80–91 vs. 73–80, mode = 80) (Tables 4, 9, 12).

*Coregonussuspensus* – *Coregonusgutturosus*

*Coregonussuspensus* differs from *C.gutturosus* by having a longer pelvic fin base (3.3–3.8% SL, mean = 3.7 vs. 3.7–4.4% SL, mean = 4.1), a shorter pelvic fin (15–16.1% SL, mean = 15.3 vs. 15.4–19.1% SL, mean = 17.1), a shorter pectoral fin (PecF2: 15.3–17.2% SL, mean = 16.4 vs. 16.8–20.3% SL, mean = 18.2), an anteriorly shorter erected dorsal fin (16.9–17.8% SL, mean = 17.6 vs. 17.6–21.6% SL, mean = 19.3), a shorter prepelvic length (47.9–51% SL, mean = 49.2 vs. 50.4–54.1% SL, mean = 52.7), a shallower head (63.6–70.8% HL, mean = 67.5 vs. 69.9–80.6% SL, mean = 74.2), a longer lower jaw (37.1–41.6% HL, mean = 40.1 vs. 34.3–39.1% HL, mean = 36.6), a shallower snout (6.5–9.9% HL, mean = 8.1 vs. 9.3–11.9% HL, mean = 10.2), longer gill rakers (middle gill raker: 11.5–14.3% HL, mean = 13.1 vs. 4.1–8.7% HL, mean = 6.9; longest gill raker: 13.2–16.4% HL, mean = 14.7 vs. 6.7–10.6% HL, mean = 8.2) and fewer gill rakers (33–37 vs. 16–21, mode = 17, 18, 19) (Tables 4, 9, 13).

*Coregonussuspensus* – *Coregonusarenicolus*

*Coregonussuspensus* can be differentiated from *C.arenicolus* by having a shorter pelvic fin base (3.3–3.8% SL, mean = 3.7 vs. 3.9–4.6% SL, mean = 4.4), a shorter pelvic fin (15–16.1% SL, mean = 15.3 vs. 16.8–18.1% SL, mean = 17.3), a shorter pectoral fin base (3–3.2% SL, mean = 3.1 vs. 3.2–3.5% SL, mean = 3.4), an anteriorly shorter erected and depressed dorsal fin (erected dorsal fin: 16.9–17.8% SL, mean = 17.6 vs. 18–20.3% SL, mean = 19.2; depressed dorsal fin: 18.2–19.8% SL, mean = 19.1 vs. 19.3–21.9% SL, mean 20.5), an anteriorly shorter anal fin (11.6–12.4% SL, mean = 11.9 vs. 12.9–13.8% SL, mean = 13.3), a shallower caudal peduncle (7.1–7.5% SL, mean = 7.3 vs. 7.7–8.2% SL, mean = 8.1), a shorter dorsal head length (12.8–14.5% SL, mean = 13.8 vs. 14.8–15.3% SL, mean = 15.1), being shallower bodied (21.5–25.1% SL, mean = 23.6 vs. 24.4–27.1% SL, mean 26.2), a larger eye (eye diameter: 21.2–22.3% HL, mean = 21.8 vs. 17.3–19.6% HL, mean = 17.7; eye height: 20.8–23.3% HL, mean = 22.2 vs. 18.8–20.8% SL, mean = 19.6), a shorter mouth width (8.9–9.7% HL, mean = 9.2 vs. 10–11% HL, mean = 10.5), a shallower snout (6.5–9.9% HL, mean = 8.1 vs. 9.7–12.3% HL, mean = 10.9), a less wide head (45.5–50% HL, mean = 48 vs. 50.5–51.8% HL, mean = 50.8), a less wide interorbital width (26.5–28.6% HL, mean = 27.5 vs. 28.8–30.8% HL, mean = 29.7), a less wide lower jaw (22.1–24.5% HL, mean = 23.1 vs. 24.9–27.2% HL, mean = 26.4), longer gill rakers (middle gill raker: 11.5–14.3% HL, mean =13.1 vs. 9.8–10.6% HL, mean = 10.2; longest gill raker: 13.2–16.4% HL, mean = 14.7 vs. 10.9–12% HL, mean = 11.5), fewer predorsal scales (31–34, mode = 32 vs. 36–44) and more gill rakers (33–37 vs. 22–31) (Tables 4, 9, 12, 13).

###### Distribution and notes on biology.

*Coregonussuspensus* occurs in all basins of Lake Lucerne (Fig. 2) except possibly in Lake Alpnach. It has been caught during the spawning season in all basins except for Lake Alpnach, where no nets were set by Hudson et al. (2016). There is no isotopic or stomach content data available for *C.suspensus* to infer the prey spectrum of this species. *Coregonussuspensus* is a medium-sized whitefish species and the size (i.e., standard length) at 3 years of age of *C.suspensus* is smaller than that of *C.litoralis*, considerably larger than that of *C.muelleri* and almost equal to that of *C.intermundia* (Suppl. material 1: table S3). We cannot compare its size to *C.nobilis* since only older individuals of the latter species have been caught (Hudson et al. 2016). *Coregonussuspensus* shows genetic ancestry contributions from whitefish of Lake Constance, besides its Lake Lucerne ancestry (Hudson et al. 2011, 2016). We therefore also compared it to the four described species from Lake Constance, namely *C.wartmanni* Bloch, 1784, *C.macrophthalmus* Nüsslin, 1882, *C.arenicolus* Kottelat, 1997, and the extinct *C.gutturosus* Gmelin, 1818. Early indications that *C.suspensus* may be of partially allochthonous origin, closely related to the radiation of Lake Constance with genetic contributions from Lake Lucerne, were seen in work by Douglas and Brunner (2002). They named a population from Lake Lucerne simply as ‘Blaufelchen’ (i.e., the local name for the species *C.wartmanni* from Lake Constance), which grouped in their study closest to Lake Constance taxa and another species of partially allochthonous origin from Lake Thun, *C.acrinasus*. More recent work confirmed these findings, showing that *C.suspensus* has a genetic affinity (based on pairwise DAPC, neighbour-joining tree and private allele analyses) with *C.wartmanni* from Lake Constance (Hudson et al. 2016; Suppl. material 1: fig. S3). Interestingly, *C.suspensus* may not just share a genetic affinity with *C.wartmanni* but also a phenotypic affinity. During the spawning season sexually mature individuals of *C.suspensus* were caught exclusively in the open water, which may suggest that they also spawn in the open water. This unique spawning behaviour has so far only been documented for individuals of *C.wartmanni* from Lake Constance (Brehm 1884; Fatio 1890; Steinmann 1950). Further research is needed to resolve if the same spawning behaviour is present in *C.suspensus*. It cannot be inferred solely based on the catch locality, although all ripe individuals of *C.suspensus* were caught with pelagic nets in the pelagic habitat (Hudson et al. 2016). This is not unique to this species though, as also *C.litoralis* and *C.intermundia* can be caught during the spawning season in the pelagic waters. However, in contrast to *C.suspensus* they seem to move from the pelagic into the benthic habitat at the time around sunset and are thus caught in the benthic habitat during spawning (Hudson et al. 2016). *Coregonussuspensus* has most likely a short spawning season in winter around the month of December. The spawning season lasts for approx. two weeks. Since the 1940s whitefish fry have been stocked from Lake Constance (among other lakes, see discussion) into Lake Lucerne, specifically in Lake Alpnach (Svarvar and Müller 1982; E. Odermatt and J. Muggli, pers. comm.). Nothing is written about the time span of these introductions nor which quantity of fry from Lake Constance were introduced into Lake Lucerne. Steinmann (1950) reported individuals not corresponding to any of the known Lake Lucerne whitefish species and questioned if additional unknown species were residing in Lake Lucerne. Specifically, Steinmann (1950) mentions an increase of whitefish individuals in 1949–1950 that have been caught mostly in the basins ‘Gersauerbecken’, ‘Urnerbecken’ and Lake Alpnach (Fig. 2), which resemble in gill raker number individuals of *C.nobilis*, but which spawn in December in the upper water column (i.e., in German ‘oberflächlich’). These individuals have been named by the commercial fishermen after the colouration of their dorsum, which was blueish, as ‘Blaufelchen’ and were even noted in the commercial fisheries statistics of Lake Lucerne for a certain time period (J. Muggli, pers. comm.). This is the same local name that is given to *C.wartmanni* from Lake Constance. This could possibly be the first mention of *C.suspensus* in the scientific literature. However, already in the year 1661 Cysat described three forms of large-type whitefish in Lake Lucerne, the ‘Krautbalchen’, the ‘Schwembalchen’ and the ‘Steinbalchen’ and a local fisherman, J. Blättler, mentioned in 1908 that he was able to distinguish between three forms of large-type whitefish in the lake (Muggli 2015). It is thus unclear, if *C.suspensus* derived from recent allochthonous stocking and/or if one of these three forms may partially be what we know today as *C.suspensus*.

###### Etymology.

The specific name suspensus in Latin means being ‘suspended’ or ‘levitating’. The name refers to the fact that this species has only been caught during the spawning season in the pelagic water column. An adjective.

###### Common names.

This species was not recognised by the local fisheries, fisheries authorities, researchers, or the public before the work by Lundsgaard-Hansen (2009) and Hudson et al. (2016). Lundsgaard-Hansen (2009) and Hudson et al. (2016) named the species as *Coregonus* sp. ‘*pelagic-intermediate*’, in German ‘pelagischer Schwebbalchen’.

##### ﻿ Coregonus nobilis

Taxon classificationAnimaliaSalmoniformesCoregonidae

Haack, 1882

B19C84DF-C149-55B7-8082-E51710D45A40

**Figure 6.**
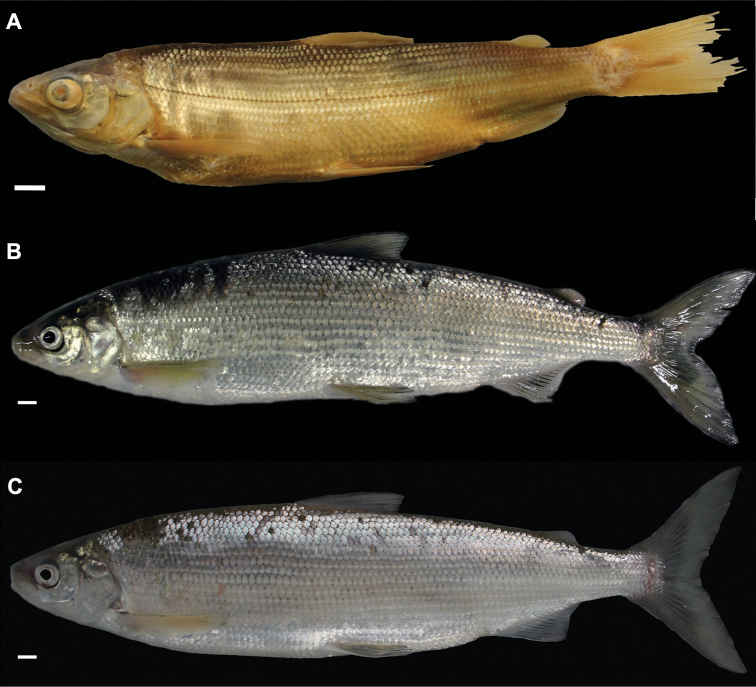
*Coregonusnobilis*, Lake Lucerne, Switzerland **A** neotype, MHNG-656.056, 207 mm SL, sex unknown, preserved specimen, (right side, reversed) **B** non-type, NMBE-1078071, freshly caught specimen, 287 mm SL, male **C** non-type, freshly caught specimen, not preserved, Lake Lucerne, 280 mm SL, sex unknown. Scale bars: 1 cm.

Figs 6 , 14 , Tables 5 , 10 , 12 , 13 , 14 , Suppl. material 1: figs S2, S3, tables S1, S2

Coregonus crassirostris : Fatio 1885.
Coregonus ‘Edelfisch’: Steinmann 1950.
Coregonus lavaretus nat. *riusensis*, oekot. *pelagicus*: Steinmann 1950.
Coregonus nobilis : Kottelat 1997; Kottelat and Freyhof 2007; Müller 2007; Hudson et al. 2011, 2013, 2016; Ingram et al. 2012; Vonlanthen et al. 2012; Alexander et al. 2017a.
Coregonus wartmanni nobilis : Fatio 1890; Birrer and Schweizer 1936a.

###### Material examined.

***Neotype*.** Historical specimen (year 1885): MHNG-656.056, 207 mm SL, sex unknown; Switzerland: Lake Lucerne. ***Non-types*.** All from Switzerland, Lake Lucerne: Contemporary specimens (year 2005): NMBE-1078053-1078063, NMBE-1078065-1078074, *N* = 21, 253–315 mm SL. Historical specimens (years 1882, 1885): MHNG-807.26, *N* = 2, 247 and 254 mm SL.

###### Diagnosis.

*Coregonusnobilis* is a medium-sized whitefish with weak pigmentation of the pectoral and pelvic fin and moderate pigmentation of the anal, caudal, adipose and dorsal fin and body; pectoral and pelvic fin with yellowish undertone; pale olive-brown colouration on the flanks above the lateral line with pale greenish undertone that is most pronounced on dorsal part of the head; moderate number of pigmented small dots on the scales on the flanks; slender, elongated, and slightly torpedo-like body; caudal peduncle elongated (12.4–15.3% SL, mean = 13.9); tip of snout is fleshy and ranges from being square-like to roundish; large eye (eye diameter: 23.5–25.6% HL, mean = 25.6) with a thick (4.6–5% HL, mean = 4.7) and triangular eye socket; many and long gill rakers (longest gill raker: 15% HL; total gill raker number = 41).

###### Description.

***Shape***: Slender body. Slender bodied with greatest body depth anterior of dorsal fin resulting in a slightly torpedo-like form. Dorsal and ventral profile similar and slightly arched. Dorsal and ventral profile from tip of snout to interorbital area mostly straight and then slightly convex to dorsal and pelvic fin origin respectively. Head moderately short. Mouth long and terminal or subtly sub-terminal. Lower jaw wide. Rostral plate equally wide as deep, quite pronounced with tip of snout fleshy and either roundish or blunt (square-like form). The eye is moderately large and that of historical specimens is larger than of contemporary specimens. Eye-socket thick and sickle cell-shaped. Pectoral fin moderately tapered and moderately long. Dorsal fin moderately long. Caudal peduncle narrow and elongated with caudal fin moderately forked and sometimes moderately to strongly asymmetrical. ***Meristics***: Many and long gill rakers. ***Colour***: Pigmentation of fins and body overall weak in live specimens. Pectoral and pelvic fin mostly transparent with yellowish ground colour, which is more pronounced in pectoral fin than pelvic fin. Pectoral fin rarely pigmented at distal part of fin and pelvic fin rarely moderately pigmented at median to distal parts of fin. Dorsal, anal, caudal and adipose fin moderately pigmented. Body weakly pigmented. Silvery appearance along flanks with moderate amount of pigmented small dots (aggregation of melanophores) on scales along flank and dorsum. Dorsally above the lateral line silvery appearance changes to a pale olive-brown and with a pale greenish ground colour that is most pronounced on dorsal part of head. Dorsal part of head and snout around nostrils moderately pigmented. Pre-operculum and operculum silvery with one black spot on lower margin of pre-operculum. Preserved specimens pale in colouration with similar pigmentation as described for live specimens. Silvery, translucent, not coloured or unpigmented parts of body become brown-yellowish, whereas pigmented parts are conserved and coloured parts (dorsally above the lateral line) become brownish.

###### Differential diagnosis.

We compare the contemporary specimens of *C.nobilis* to the contemporary specimens of the other species from Lake Lucerne. The differential diagnoses between *C.litoralis*, *C.intermundia* and *C.suspensus*, and *C.sarnensis* are given under those species’ accounts.

*Coregonusnobilis* – *Coregonusmuelleri*

*Coregonusnobilis* differs from *C.muelleri* by having an anteriorly shorter erected and depressed dorsal fin (erected dorsal fin: 15.3–17.2% SL, mean = 16.3 vs. 16.4–20.3% SL, mean = 18.1; depressed dorsal fin: 16.7–18.8% SL, mean = 17.8 vs. 17.3–21.1% SL, mean = 19.2), a shorter caudal fin (20.1–23.8% SL, mean = 22.3 vs. 22–26.4% SL, mean = 23.9), a shorter dorsal head length (12.8–14.9% SL, mean = 13.6 vs. 14.4–17% SL, mean = 15.7), smaller eye (eye diameter: 20.2–23.1% HL, mean = 21.8 vs. 22.2–26% HL, mean = 24.1; eye cavity: 24.2–28% HL, mean = 26 vs. 26.4–31.1% HL, mean = 27.7; eye height (20.7–24% HL, mean = 22.3 vs. 22.3–26.2% HL, mean = 24.3), differently shaped eye socket (sickle cell-shaped vs. roundish), a deeper head and snout (head depth: 65.9–77.8% HL, mean = 71.2 vs. 59.6–70.8% HL, mean = 63.7; snout depth: 7.9–12.4% HL, mean = 10.5 vs. 4.9–8.7% HL, mean = 7) and a wider head and lower jaw (head width: 46.2–55% HL, mean = 50.6 vs. 37.2–48.7% HL, mean = 43.8; lower jaw width: 7.8–10.2% HL, mean = 9 vs. 5.9–8.7, mean = 7.3) (Tables 5, 6). Based on ratios *C.nobilis* can be differentiated from *C.muelleri* by having a smaller ‘eye diameter / head depth’ ratio (ED/HD: 0.27–0.33 vs. 0.35–0.42) (Table 10).

###### Distribution and notes on biology.

*Coregonusnobilis* occurs in all basins of Lake Lucerne (Fig. 2) except for Lake Alpnach (Nufer 1905). Since the species is endangered no commercial and recreational fisheries are permitted all year round (details on the decline of the population of *C.nobilis* and its putative extinction and its rediscovery is discussed in the Suppl. material 1: Paragraph 3). Hence, to understand the distribution of *C.nobilis* outside of the spawning season we can only consult historical catch records. These suggest that it was caught in large quantities and migrated heavily throughout the year within the basins of Lake Lucerne except for Lake Alpnach (Birrer and Schweizer 1936a; Steinmann 1950). Specifically, it was caught in water depths of 5–25 m in the pelagic habitats in the basins ‘Kreuztrichter’, ‘Gersauerbecken’, ‘Urnerbecken’, ‘Vitznauerbecken’ and ‘Küssnachtersee’ (Birrer and Schweizer 1936a; Fig. 2). *Coregonusnobilis* feeds – depending on the time of the year – on different pelagic prey (i.e., variety of zooplankton such as *Bythotrephes*, *Daphnia*, *Bosmina*, Copepoda, Leptodora, and rarely Chironomidae) (Birrer and Schweizer 1936a). It is a medium-sized whitefish species and has a moderate growth rate (Müller et al. 2007). It is the only whitefish species in Lake Lucerne that spawns in late summer in great water depths. The spawning season of *C.nobilis* stretches from late July to mid-September in water depths of 80 to 214 m (Birrer and Schweizer 1936a; Müller 2007; Vonlanthen et al. 2012; Hudson et al. 2016; Alexander et al. 2017a). During the spawning season it has been caught both historically and more recently in the inner basins of Lake Lucerne; ‘Gersauerbecken’ (Birrer and Schweizer 1936a; Hudson et al. 2016) and ‘Vitznauerbecken’ (Alexander et al. 2017a). Birrer and Schweizer (1936a) note that the spawning grounds can also be found in the deeper waters at gravel beds that are fed by nearby stream mouths.

###### Common name.

Edelfisch.

##### ﻿ Coregonus muelleri sp. nov.

Taxon classificationAnimaliaSalmoniformesCoregonidae

B83A0FC4-C766-539C-B0F7-4E1947EE3E16

https://zoobank.org/1D01BB7F-517B-4672-BC4D-B8700D56EFB2

**Figure 7.**
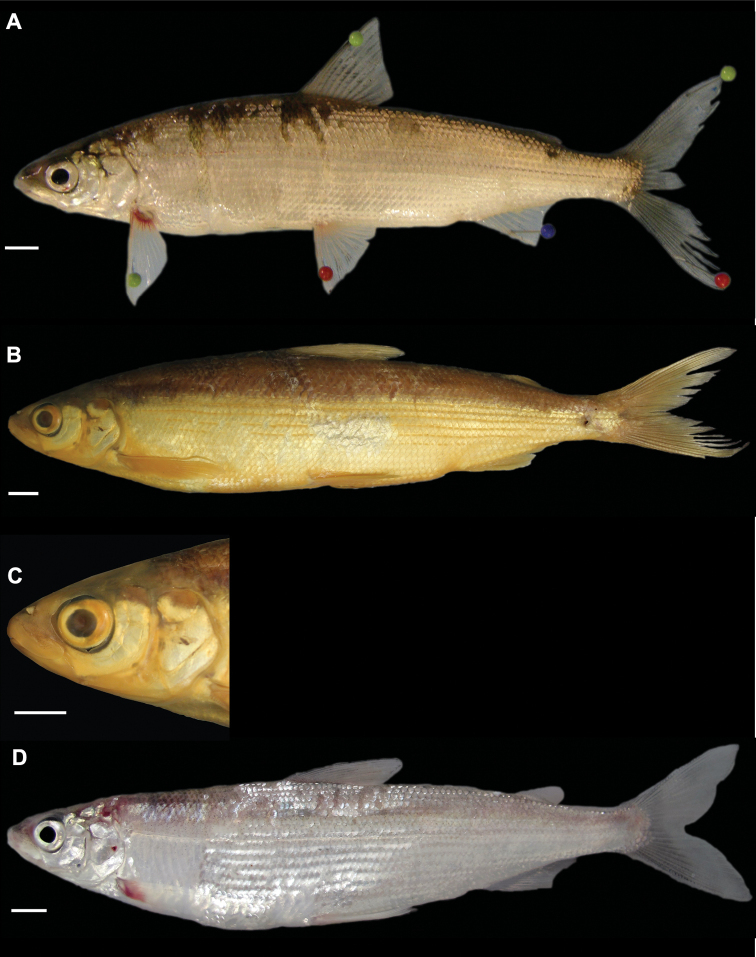
*Coregonusmuelleri*, Lake Lucerne, Switzerland **A** holotype, NMBE-1078123, 180 mm SL, male, freshly caught specimen, (right side, reversed) **B, C** holotype, NMBE-1078123, preserved specimen **D** non-type, Eawag-59186, Lake Lucerne, 175 mm SL, male. Scale bars: 1 cm.

Figs 7 , 14 , Tables 6 , 10 , 12 , 13 , 14 , Suppl. material 1: figs S2, S3, tables S1–S3

Coregonus ‘Albeli’: Steinmann 1950; Douglas et al. 1999; Douglas and Brunner 2002.
Coregonus exiguus albellus : Fatio 1890; Birrer and Schweizer 1935, 1936b.
Coregonus lavaretus nat. *riusensis*, oekot. *nanus*: Steinmann 1950 (see also synonymy of C.zugensis and C.sarnensis).
Coregonus sp. ‘small’: Hudson et al. 2016.
Coregonus ‘Weissfelchen’: Steinmann 1950.
Coregonus ‘Weissfisch’: Douglas and Brunner 2002.
Coregonus zugensis : Kottelat 1997; Kottelat and Freyhof 2007; Wedekind et al. 2007a, b; Rudolfsen et al. 2008; Hudson et al. 2011, 2013; Ingram et al. 2012; Vonlanthen et al. 2012; Lundsgaard-Hansen et al. 2013; Roesch et al. 2013; Alexander et al. 2017a (see also synonymy of C.zugensis).

###### Material examined.

***Holotype*.** Contemporary specimen (year 2007): NMBE-1078123, 180 mm SL, male; Switzerland: Lake Lucerne. ***Paratypes*.** All from Switzerland, Lake Lucerne: Contemporary specimens (year 2007): NMBE-1078106–NMBE-1078122, NMBE-1078124–NMBE-1078135, *N* = 29, 158–198 mm SL. Historical specimens (years 1892, 1940, 1947): NMBE-1076284, NMBE-1076289, NMBE-107628, NMBE-1076290 (Eawag-305-1, Eawag-305-2, Eawag-305-3, Eawag-305-4, Eawag-305-5), NMBE-1076291, *N* = 8, 158–193 mm.

###### Diagnosis.

*Coregonusmuelleri* is a small whitefish species (standard length at 3 years of age: range = 160–232, mean = 194) with weak pigmentation of all fins and body; pale olive-brown colouration on the flanks above the lateral line; elongate slender body; large eye (eye diameter: 22.2–26% HL, mean = 24.1) with a subtle triangular eye socket; tip of snout pointy; many and long gill rakers (longest gill raker: 12.5–16.7% HL, mean = 14.5; total gill raker number: 33–43).

###### Description.

***Shape***: Body elongated and slender. Greatest body depth anterior of dorsal fin. Ventral profile and dorsal profile similar and slightly arched. Dorsal and ventral profile from tip of snout to interorbital mostly straight and then slightly convex to dorsal and pelvic fin origin respectively. Head long. Snout long and tip of snout if often pointed and seldomly fleshy resulting in a not pronounced rostral plate. Mouth long and terminal. Large eye with a subtle sickle cell-shaped (seldom roundish) eye-socket in the historical specimens and a mostly roundish (seldom sickle cell-shaped) eye-socket in the contemporary specimens. The eye of the historical specimens is larger than those of the contemporary specimens. Pectoral fin moderately long and tapered. Anterior unbranched ray of the erected dorsal fin ranges from almost vertically straight to an ~ 70–80° angle to body axis and only bent slightly posteriorly at end of the ray. Caudal peduncle narrow and elongated with caudal fin forked and sometimes moderately to strongly asymmetrical with often ventral part being longer. Unbranched rays of anal fin straight and rarely bent posteriorly at end of ray. Anal fin longest anteriorly and progressively shortening posteriorly with outer margin of the anal fin slightly concave. ***Meristics***: Many and long gill rakers. ***Colour***: Pigmentation of fins and body weak in live specimens. Pectoral fin transparent and pelvic and anal fin mostly transparent. Pectoral fin very rarely pigmented at distal part of the fin and anal and pelvic fin rarely moderately pigmented at median to distal parts of fin. Dorsal, caudal and adipose fin moderately pigmented. Silvery appearance along flanks. Dorsally above the lateral line silvery appearance changes to a pale olive-brown. Dorsal part of head and snout around nostrils moderately pigmented. Pre-operculum and operculum silvery with one black spot on lower margin of the pre-operculum. Preserved specimens pale in colouration with similar pigmentation as described for live specimens. In contemporary specimens silvery, translucent, not coloured or unpigmented parts of body become brown-yellowish, whereas pigmented parts are conserved and coloured parts (dorsally above the lateral line) become brownish. In historical specimens all body parts are brownish.

###### Differential diagnosis.

The differential diagnoses against the contemporary specimens of *C.litoralis*, *C.intermundia*, *C.suspensus*, *C.nobilis* and *C.sarnensis* and against the historical specimens of *C.litoralis* from Lake Lucerne and *C.zugensis* from Lake Zug are given under those species’ accounts.

###### Distribution and notes on biology.

*Coregonusmuelleri* occurs in all basins of Lake Lucerne (Fig. 2) except for Lake Alpnach (Steinmann 1950; J. Muggli, pers. comm.). *Coregonusmuelleri* was (besides *C.nobilis*) historically and is today the most abundant and commercially important whitefish species in Lake Lucerne (Birrer and Schweizer 1935; Muggli 2015; Alexander et al. 2017a). *Coregonusmuelleri* feeds – depending on the time of the year – on different pelagic prey (i.e., variety of zooplankton such as Bythotrephes, Daphnia, Bosmina, Copepoda, Leptodora and rarely Chironomidae) (Birrer and Schweizer 1935). It is a small whitefish species with the size at 3 years of age of *C.muelleri* being considerably smaller than that of *C.litoralis*, *C.intermundia* and *C.suspensus* (Suppl. material 1: table S3). We cannot compare its size to *C.nobilis* since only older individuals of the latter species have been caught (Hudson et al. 2016). It has a slow growth rate (Birrer and Schweizer 1935; Steinmann 1950). *Coregonusmuelleri* has a long spawning season with presumably two spawning peaks. The historical literature suggests that *C.muelleri* has a spawning peak in the early summer from July to August and a second peak in the late autumn to early winter from October to December at a spawning depth of between 60 to 200 m (Fatio 1890; Surbeck 1913; Birrer und Schweizer 1935; Steinmann 1950; Muggli 2015). However, according to the fishery warden J. Muggli it is unclear if these summer-spawning *C.muelleri* were once present in Lake Lucerne, at least in the last few centuries such summer-spawning *C.muelleri* have not been caught. The population of whitefish that we have described as *C.muelleri* was originally grouped under the species name *C.zugensis*. However, independent multilocus microsatellite (Suppl. material 1: figs S2, S3) and large genomic AFLP (Hudson et al. 2011) data sets have shown that the whitefish species from Lake Lucerne and the whitefish populations of Lake Zug, are two independent genetic groups suggesting that *C.muelleri* is an endemic species of Lake Lucerne and should not be grouped under the name *C.zugensis*. Furthermore, *C.muelleri* can be distinguished based on morphological characters and ratios from historical specimens of *C.zugensis*, as well as from the other two species of Lake Zug, *C.obliterus* and *C.supersum* (see details under those species’ account).

###### Etymology.

The species is named after the late Dr. Rudolf Müller, a fisheries biologist and former department head and research group leader at EAWAG, Center for Ecology, Evolution and Biogeochemistry (CEEB) in Kastanienbaum, Switzerland. He dedicated a large part of his career to understanding the ecology of whitefish in Swiss lakes, and confirmed the re-discovery of *C.nobilis*.

###### Common name.

Albeli.

#### Lake Sarnen whitefish

##### ﻿ Coregonus sarnensis sp. nov.

Taxon classificationAnimaliaSalmoniformesCoregonidae

BA3CB832-F9F2-546A-AF69-6C8564F07BDD

https://zoobank.org/34B59895-283B-4081-B964-78FFA2FF9C32

**Figure 8.**
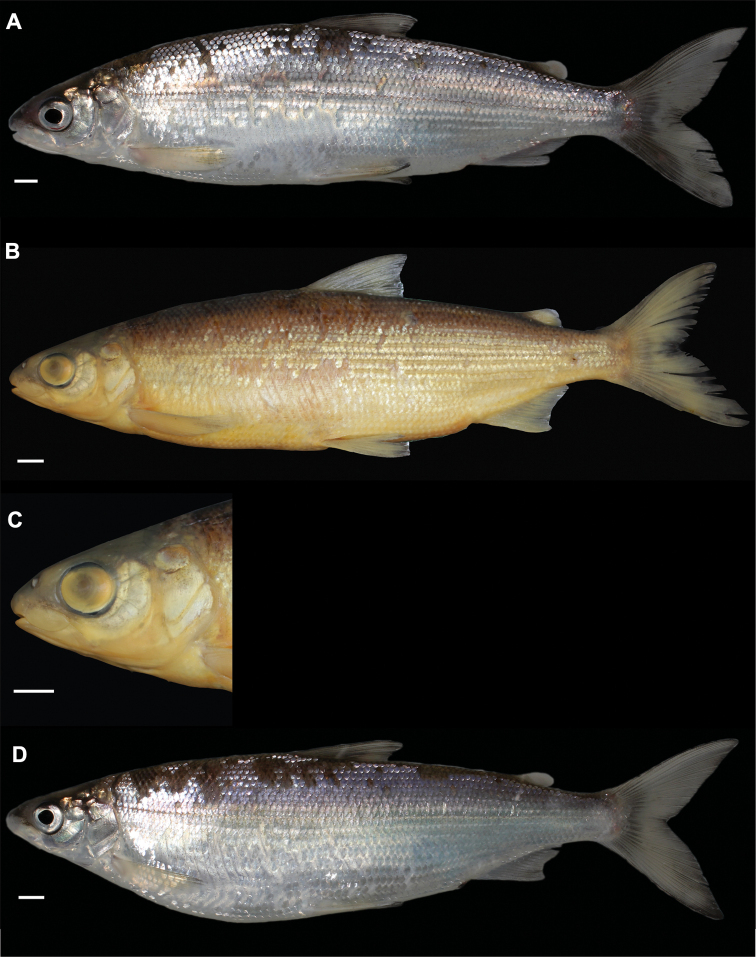
*Coregonussarnensis*, Lake Sarnen, Switzerland **A** holotype, NMBE-1078159, 230 mm SL, male, freshly caught specimen **B, C** holotype, NMBE-1078159, preserved specimen **D** paratype, NMBE-1078146, Lake Lucerne, 223 mm SL, female. Scale bars: 1cm.

Figs 8 , 14 , Tables 7 , 10 , 12 , 13 , 14 , Suppl. material 1: figs S1–S3, tables S1–S3

Coregonus lavaretus nat. *riusensis*, oekot. *primigenius*: Steinmann 1950 (see also synonymy of C.supersum, C.suidteri, and C.litoralis).
Coregonus ‘Sarnerbalchli’: Steinmann 1950.
Coregonus ‘Sarnerfelchen’: Steinmann 1950.
Coregonus sp. ‘Sarnerfelchen’: Vonlanthen et al. 2012.
Coregonus zugensis : Vonlanthen and Périat 2018 (see also synonymy of C.muelleri and C.zugensis).

###### Material examined.

***Holotype*.**NMBE-1078159, 230 mm SL, male; Switzerland: Lake Sarnen. ***Paratypes*.** All from Switzerland, Lake Sarnen: NMBE-1078143–NMBE-1078155, NMBE-1078157, NMBE-1078158, NMBE-1078160–NMBE-1078169, NMBE-1078171 – NMBE-1078173, NMBE-1078174, NMBE-1078175, *N* = 27, 188–261.5 mm SL.

###### Diagnosis.

*Coregonussarnensis* is a medium-sized whitefish (standard length at 3 years of age: range = 188–223, mean = 211) with weak pigmentation of the pectoral and pelvic fin and moderate pigmentation of the anal, caudal, adipose and dorsal fin and body; pale green to pale olive-brown colouration on the flanks above the lateral line; moderate number of pigmented small dots on the scales on the flanks; slender, elongated body; caudal peduncle mostly elongated (caudal peduncle depth: 7.1–8.6% SL, mean = 7.7; caudal peduncle length: 11.5–14.1% SL, mean = 12.6); tip of snout is fleshy, roundish, and often has an ~ 40–50° angle to the body axis anterior of the eye, such that the profile from the tip of the snout to the vertical projection where the anterior part of the eye crosses the dorsal profile is straight and afterwards slightly convex; large eye (eye diameter: 22.2–27.1% HL, mean = 23.9); many and long gill rakers (longest gill raker: 10.6–15.8% HL, mean = 13; total gill raker number: 33–40).

###### Description.

***Shape***: Body elongated and slender. Greatest body depth anterior of dorsal fin resulting in a slightly torpedo-like form. Ventral profile and dorsal profile similar and slightly arched. Dorsal and ventral profile from tip of snout to interorbital mostly straight and then slightly convex to dorsal and pelvic fin origin respectively. Head moderately long. Snout often has an ~ 40–50° angle to body axis anterior of eye, such that profile from tip of snout to vertical projection where anterior part of eye crosses dorsal profile is straight and afterwards slightly convex. Tip of snout if often roundish and seldomly fleshy resulting in a not strongly pronounced rostral plate. Mouth thick (i.e., width of upper and lower jaw), long and often terminal and only rarely slightly sub-terminal. Large eye with a variable eye-socket shape ranging from sickle cell-shaped to roundish. Pectoral fin moderately long and tapered. Anterior unbranched ray of erected dorsal fin ranges from almost vertically straight to an ~ 70–80° angle to body axis and only bent slightly posteriorly at end of ray. Caudal peduncle often narrow and elongated (seldomly stout) with caudal fin forked. Unbranched rays of anal fin straight. Anal fin longest anteriorly and progressively shortening posteriorly with outer margin of anal fin slightly concave. ***Meristics***: Many and long gill rakers. ***Colour***: Pigmentation of fins and body moderate in live specimens. Pectoral fin transparent and pelvic and anal fin mostly transparent. Pectoral fin is only pigmented at distal part of fin and anal and pelvic fin are moderately pigmented at median to distal parts of fin. Dorsal, caudal and adipose fin moderately pigmented. Silvery appearance along flanks. Dorsally above lateral line silvery appearance changes from a pale green colouration (e.g., to an olive-green colouration. Dorsal part of head and snout around nostrils moderately pigmented. Pre-operculum and operculum silvery with one black spot on lower margin of pre-operculum. Along flank and dorsum moderately pigmented small dots (aggregation of melanophores) on scales. Preserved specimens pale in colouration with similar pigmentation as described for live specimens. Silvery, translucent, not coloured or unpigmented parts of body become brown-yellowish, whereas pigmented parts are conserved and coloured parts (dorsally above lateral line) become brownish.

###### Differential diagnosis.

*Coregonussarnensis* – *Coregonuslitoralis*

*Coregonussarnensis* can be differentiated from *C.litoralis* by having a shorter dorsal fin base (9.5–12.7% SL, mean = 11.5 vs. 11.9–15.1% SL, mean = 13.4), longer head (20.8–24% SL, mean = 22 vs. 19.2–20.9% SL, mean = 19.9), larger eye (eye diameter: 22.1–27.1% HL, mean = 23.9 vs. 18.2–21.4% HL, mean = 20.2; eye cavity: 26.3–31% HL, mean = 28.6 vs. 22.7–26% HL, mean = 24.6), a shallower head (62.7–74.3% HL, mean = 67.8 vs. 70.1–77.9% HL, mean = 73.8), less wide interorbital width (23.8–28.1% HL, mean = 26.2 vs. 26.3–29.8% HL, mean = 28.2), longer gill rakers (gill raker: 10.6–15.8% HL, mean = 13 vs. 9.2–13.1% HL, mean = 11) and more gill rakers (33–40, mode = 35, 36 vs. 24–32, mode = 26, 30) (Tables 2, 7, 13). Based on ratios *C.sarnensis* can be differentiated from *C.litoralis* by having a larger ‘eye depth / head depth’ ratio (ED/HD: 0.33–0.39 vs. 0.25–0.30) (Table 10)

**Table 7.** Morphological and meristic data of *Coregonussarnensis* from Lake Sarnen, NMBE-1078159 holotype, contemporary specimen; paratypes of contemporary specimens *N* = 27. For males and both sexes the holotype is included in the range and mean.

Species	*Coregonussarnensis*						
Morphological characters	Holotype	Holotype + Paratypes		Paratypes		Holotype + Paratypes	
		Ntotal = 28		Nfemales = 7		Nmales = 21	
		mean	range	mean	range	mean	range
**Standard length (mm)**	230	221.8	188–262	219	207–242	222.7	188–262
**Percentage of standard length**							
Pelvic fin base	3.9	3.9	3.3–4.6	3.8	3.4–4.4	4	3.3–4.6
Pelvic fin ‘spine’ length	6.2	6.2	4.8–7.1	6	4.8–7	6.2	5.1–7.1
Pelvic fin length	15.7	17	15.4–18.3	16.4	15.4–18.3	17.1	15.7–18.3
Pectoral fin base	3.1	3.2	2.7–3.8	3.1	2.8–3.4	3.3	2.7–3.8
Pectoral fin 1 length	17	17	15.4–19.4	16.6	15.7–18	17.2	15.4–19.4
Pectoral fin 2 length	18.2	18	16–21.3	17.6	16.2–19.1	18.2	16–21.3
Dorsal fin base	11	11.5	9.5–12.7	11.4	10.9–12.7	11.5	9.5–12.6
Length of anterior part of dorsal fin erected	16.7	18.3	16.7–19.5	18.3	17–19.4	18.3	16.7–19.5
Length of anterior part of dorsal fin depressed	18.4	19.8	17.8–21.6	19.5	17.8–20.5	19.9	18.4–21.6
Length of posterior part of dorsal fin erected	5.7	5.6	4.6–6.5	5.6	4.8–6.2	5.5	4.6–6.5
Anal fin base	12.2	12.2	10.5–13.9	12.3	11.6–13.9	12.2	10.5–13.4
Length of anterior part of the anal fin	12.5	12.8	11.9–14.2	12.6	11.9–14	12.9	11.9–14.2
Adipose fin base	5.2	5	3.8–6.3	5.1	3.9–5.7	5	3.8–6.3
Caudal fin length	25	24.2	22.4–26.4	24.2	22.5–26.4	24.2	22.4–25.5
Caudal peduncle depth	7.8	7.7	7.1–8.6	7.7	7.1–8.6	7.7	7.2–8.2
Caudal peduncle length	13	12.6	11.5–14.1	12.4	11.5–13.7	12.6	11.7–14.1
Length from posterior part of adipose fin to caudal fin base	18.2	18.2	15.5–21.7	18.3	15.5–21.7	18.2	16.2–20
Dorsal head length	16.2	15.4	14.1–17	15.4	14.2–17	15.4	14.1–16.6
Prepelvic length	50.5	51.4	49–55.5	52.2	49.6–55.5	51.1	49–54
Preanal length	75.7	77.8	75.5–81.3	78.4	76.2–81.3	77.7	75.5–79.2
Predorsal length	49.7	47.7	44.1–51.5	47.5	44.1–51.2	47.8	45.1–51.5
Body depth	25.9	25.3	22.4–27.8	25.4	24–27.8	25.3	22.4–27.5
Postdorsal length	42.5	42.9	39.4–46.2	42.3	39.4–46.2	43	41.2–45.8
Head length	23.1	22	20.8–24	21.9	20.8–24	22	20.8–23.8
Total length	123.7	121.9	119.3–124.6	121.7	120.5–123.6	122	119.3–124.6
**Head length (mm)**	53.1	48.8	40.2–58.5	48.1	43–55.4	49	40.2–58.5
**Percentage of head length**							
Snout length	23.6	22.4	19.8–24.3	21.9	20.1–24.3	22.5	19.8–23.9
Eye diameter	25.2	23.9	22.1–27.1	24	23–24.7	23.9	22.1–27.1
Eye cavity	27.7	28.6	26.3–31	29.2	28.9–29.8	28.4	26.3–31
Eye height	25.1	24	21–26.3	24.8	23.2–25.6	23.7	21–26.3
Eye socket	3.8	4.3	3.3–6.2	4.1	3.3–5.6	4.4	3.3–6.2
Postorbital length	50.5	51	47.7–55.9	51.4	49.8–54	50.8	47.7–55.9
Head depth	69.9	67.8	62.7–74.3	68	65.3–71.7	67.8	62.7–74.3
Mouth width	9.3	9.4	7.6–10.9	8.9	7.6–10.2	9.5	8.4–10.9
Upper jaw length	30.2	28.7	26.8–30.9	29.1	27.8–30.9	28.6	26.8–30.8
Lower jaw length	39.8	40.9	37.1–43.3	41.3	40.4–42.2	40.8	37.1–43.3
Length of maxilla	22.8	21.2	18.8–23.5	21.3	19.1–23.5	21.2	18.8–23
Snout depth	6.5	7.6	6.2–9.2	7.7	6.4–8.9	7.5	6.2–9.2
Snout width	14.5	15.5	12.5–18.1	15.5	14–16	15.5	12.5–18.1
Head width	51.7	47.8	43.7–53.3	47.6	44.8–50.3	47.9	43.7–53.3
Interorbital width	26.8	26.2	23.8–28.1	26.6	24.5–28.1	26	23.8–27.7
Internarial width	12	12	10.6–13.6	11.9	10.6–12.7	12.1	10.7–13.6
Lower jaw length	7.9	8.2	6.7–9.2	7.8	6.7–9.2	8.3	6.9–9.2
Lower jaw width	23	22.7	18.9–26.9	23	20.8–26.9	22.5	18.9–26.9
Middle gill raker length	10.7	11.5	9.2–14.7	11.1	9.5–12.4	11.6	9.2–14.7
Longest gill raker length	11.5	13	10.6–15.8	12.6	11.6–14.9	13.1	10.6–15.8
Upper arch length	19.6	18.2	15.6–21.4	18.7	16.6–20.4	18.1	15.6–21.4
Lower arch length	28.1	33.9	28.1–38.1	34.3	31.9–36.7	33.8	28.1–38.1

*Coregonussarnensis* – *Coregonusintermundia*

*Coregonussarnensis* can be differentiated from *C.intermundia* by having a longer head (20.8–24% SL, mean = 22 vs. 19.1–21.5% SL, mean = 20.3) and a larger eye cavity (26.3–31% HL, mean = 28.6 vs. 23.8–27.9% HL, mean = 25.9) (Tables 3, 7).

*Coregonussarnensis* – *Coregonussuspensus*

*Coregonussarnensis* can be differentiated from *C.suspensus* by having longer pectoral fins (pectoral fin 2: 16–21.3% SL, mean = 18 vs. 15.3–17.2% SL, mean = 16.4), a longer dorsal head length (14.1–17% SL, mean = 15.4 vs. 12.8–14.5% SL, mean = 13.8), longer head (20.8–24% SL, mean = 22 vs. 18.7–20.4% SL, mean = 19.6) and larger eyes (eye diameter: 22.1–27.1% HL, mean = 23.9 vs. 21.2–22.3% HL, mean = 21.8; eye cavity: 26.3–31% HL, mean = 28.6 vs. 25.1–26.4% HL, mean = 25.6) (Tables 4, 7). Based on ratios *C.sarnensis* can be differentiated from *C.suspensus* by having a smaller ‘predorsal length / eye depth’ ratio (PreD/ED: 7.42–10.21 vs. 10.73–11.40) (Table 10).

*Coregonussarnensis* – *Coregonusnobilis*

*Coregonussarnensis* can be differentiated from *C.nobilis* by having a longer erected and depressed anterior part of dorsal fin (erected: 16.7–19.5% SL, mean = 18.3 vs. 15.3–17.2% SL, mean = 16.3; depressed: 17.8–21.6% SL, mean = 19.8 vs. 16.7–18.8% SL, mean = 17.8), longer caudal fin (22.4–26.4% SL, mean = 24.2 vs. 20.1–23.8% SL, mean = 22.3), deeper caudal peduncle (7.1–8.6% SL, mean = 7.7 vs. 6.3–7.6% SL, mean = 6.8), longer dorsal head length (14.1–17% SL, mean = 15.4 vs. 12.8–14.9% SL, mean = 13.6), larger eye (eye diameter: 22.1–27.1% HL, mean = 23.9 vs. 20.2–23.1% HL, mean = 21.8; eye cavity: 26.3–31% HL, mean = 28.6 vs. 24.2–28% HL, mean = 26) and a shallower snout (6.2–9.2% HL, mean = 7.6 vs. 7.9–12.4% HL, mean = 10.5) (Tables 5, 7). Based on ratios *C.sarnensis* can be differentiated from *C.nobilis* by having a smaller ‘postdorsal length / eye cavity’ ratio (PostD/EC: 5.94–7.94 vs. 7.87–9.77) (Table 10).

*Coregonussarnensis* – *Coregonusmuelleri*

*Coregonussarnensis* can be differentiated from *C.muelleri* by having a deeper caudal peduncle (7.1–8.6% SL, mean = 7.7 vs. 6.3–7.4% SL, mean = 6.9), being deeper bodied (22.4–27.8% SL, mean = 25.3 vs. 19.8–24.9% SL, mean = 21.9), having a wider eye socket (3.3–6.2% SL, mean = 4.3 vs. 1.9–4.6% SL, mean = 3) and wider head (43.7–53.3% SL, mean = 47.8 vs. 37.2–48.7% SL, mean = 43.8) (Tables 6, 7).

###### Distribution and notes on biology.

*Coregonussarnensis* occurs in Lake Sarnen. It has further been identified by genetic assignments to be present in Lake Alpnach (Suppl. material 1: figs S2, S3), which it most likely colonised naturally over the River Sarner Aa. It has also been shown to be present south of the alps in Lake Maggiore. The ‘Bondella’ (local name of the population) of Lake Maggiore derives from translocations and has been shown to group in a neighbour-joining tree with *C.sarnensis* from Lake Sarnen (Hudson et al. 2011). Lake Sarnen was once part of Lake Lucerne (connected through Lake Alpnach respectively) and got disconnected by amassing of sediments from the rivers Grosse Schlieren, Kleine Schlieren, and Grosse Melchaa several thousand years ago (Steinmann 1950; Pfiffner 2021). The occurrence of whitefish scales in sediment cores from Lake Sarnen that predate (scales found in the sediment layer of 1861–1857 (Suppl. material 1: fig. S1)) the earliest allochthonous whitefish introductions (1888) and independent genetic grouping of the Lake Sarnen population (AFLP data: Hudson et al. 2011; Microsattelite data: Suppl. material 1: figs S2, S3) from the lakes that seeded the allochthonous whitefish introductions (lakes Sempach, Zug, Lucerne) suggest that Lake Sarnen harbours an endemic whitefish species (this is discussed in more detail in the Suppl. material 1: paragraph 1). Information on the spawning season of *C.sarnensis* derive from two targeted spawning events by O.M. Selz and the fishery warden A. von Deschwanden in November of the year 2018 (pers. comm.). Thus, the spawning season of *C.sarnensis* is known only for November (although it may be stretching into December based on anecdotal information from recreational fishermen, which see large aggregations of putative whitefish in the deeper waters in December on their echosounders and in some cases they catch pike at these places with ripe whitefish in their stomachs in December) and it spawns at depth of 20 metres down to the lake bottom at 50 metres.

###### Etymology.

The specific name *sarnensis* refers to Sarnen, a village on the shores of the lake to which it gave its name. An adjective.

###### Common names.

Sarnerfelchen, Sarneralbeli.

#### Lake Zug whitefish

##### ﻿ Coregonus supersum sp. nov.

Taxon classificationAnimaliaSalmoniformesCoregonidae

E28F2697-CFC6-530B-8FFB-9929C9CCD124

https://zoobank.org/7502CA0A-1E34-4DC5-825D-DFEE7F4B1E0C

**Figure 9.**
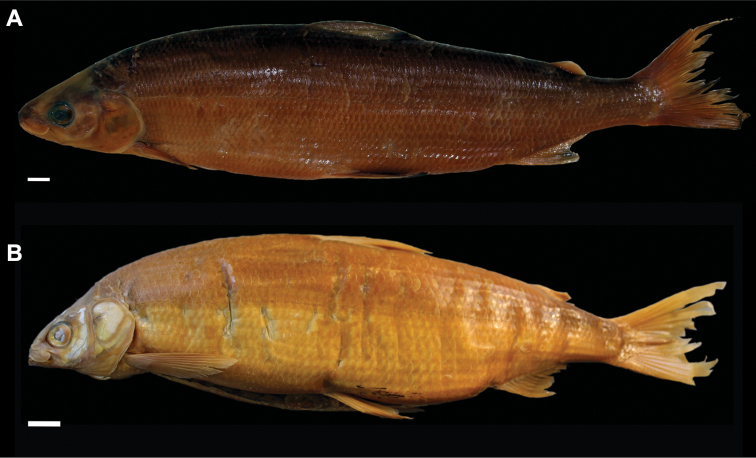
*Coregonussupersum*, Lake Zug, Switzerland **A** holotype, NMBE-1076275, 288mm SL, male, preserved specimen **B** paratype, MHNG-2786.063, 286mm SL, sex unknown, preserved specimen. Scale bars: 1 cm

Figs 9 , 14 , Tables 8 , 10 , 11 , 12 , 13 , 14 , Suppl material 1: figs S2, S3, table S13

Coregonus ‘Balchen’: Douglas and Brunner 2002.
Coregonus crassirostris compactus : Fatio 1885 (see also synonymy of C.zugensis).
Coregonus lavaretus nat. *riusensis*, oekot. *primigenius*: Steinmann 1950 (see also synonymy of C.sarnensis, C.suidteri, and C.litoralis).
Coregonus schinzii helveticus var. zugensis : Fatio 1890 (see also synonymy of C.litoralis)
Coregonus suidteri : Kottelat 1997; Kottelat and Freyhof 2007; Vonlanthen et al. 2012, 2015 (see also synonymy of C.litoralis and C.suidteri).
Coregonus sp. ‘Zugerbalchen’: Vonlanthen et al. 2012.
Coregonus sp. ‘Zugerseebalchen’: Steinmann 1950.

###### Material examined.

***Holotype*.** Historical specimen (year 1939): NMBE-1076275, 288 mm SL, male; Switzerland: Lake Zug. ***Paratypes*.** All from Switzerland, Lake Zug: Historical specimens (years 1907, 1937, 1939, 1941): NMBE-1076265, NMBE-1076267, NMBE-1076268, NMBE-1076277, MHNG 2786.063, *N* = 5, 263–411 mm SL.

###### Diagnosis.

*Coregonussupersum* is a large whitefish species with strong pigmentation of all fins and the body; greenish blue colour on the flanks above the lateral line; deep bodied (23.2–26.6% SL, mean = 24.4); blunt snout; short head (19.8–21.8% SL, mean = 20.9); sub-terminal mouth; small eye (eye diameter: 18.2–21.7% HL, mean = 19.9) with a thick and triangular-shaped eye socket; short and stout caudal peduncle (caudal peduncle depth: 6.7–8.1% SL, mean = 7.2; caudal peduncle length: 12.2–14.4% SL, mean = 13.1); few and short gill rakers (longest gill raker: 8.9–12.4% HL, mean = 10.1; total gill raker number: 21–27).

###### Description.

***Shape***: Generally, deep bodied with greatest body depth anterior of dorsal fin. Dorsal profile from tip of snout to anterior origin of dorsal fin is strongly convex and ventral profile moderately convex or almost straight from interorbital area to the pelvic fin origin. Head short. Mouth short, wide and sub-terminal. Rostral plate pronounced and a bit wider than deep resulting in a slightly rectangular shape. Tip of snout often blunt. Small eye with a thick and mostly sickle cell-shaped eye-socket. Pectoral fin moderately tapered and moderately short. Dorsal fin long. Caudal peduncle stout and short with caudal fin moderately forked. Unbranched rays of anal fin slightly bent posteriorly. Anal fin longest anteriorly and progressively shortening posteriorly with outer margin of anal fin mostly straight and only rarely slightly concave. ***Meristics***: Few and short gill rakers. ***Colour***: Pigmentation of fins and body overall strong. Pectoral fin moderately pigmented at the median to distal parts of the fin. All other fins are strongly pigmented. Silvery appearance along the flanks. The preserved specimens have moderate to many pigmented small dots (aggregation of melanophores) on the scales along the flank and the dorsum. Dorsally above the lateral line the silvery appearance changes to a greenish blue colour. The dorsal part of the head and the snout around the nostrils is strongly pigmented. The pre-operculum and operculum are silvery with one black spot on the lower margin of the pre-operculum. Preserved specimens are pale in colouration with similar pigmentation as described for live specimens. Preserved fish are brownish in colouration.

###### Differential diagnosis.

*Coregonussupersum* – *Coregonuszugensis*

*Coregonussupersum* can be differentiated from *C.zugensis* by having a shorter head (dorsal head length: 13.6–15.4% SL, mean = 14.4 vs. 14.9–16.8% SL, mean = 15.7; head length: 19.8–21.8% SL, mean = 20.9 vs. 21.3–23.5% SL, mean = 22), a smaller eye (eye diameter: 18.2–21.7% HL, mean = 19.9 vs. 19.7–25.3% HL, mean = 22.9; eye height: 19.4–22.2% HL, mean = 20.9 vs. 21.7–26.7% HL, mean = 23.9), a longer upper and lower jaw and maxilla (upper jaw: 24.3–28% HL, mean = 26.5 vs. 26.7–33.3% HL, mean = 30.4; lower jaw: 36.3–41.6% HL, 37.9 vs. 38.4–44.6% HL, mean = 42; maxilla: 17.8–21.3% HL, mean = 18.8 vs. 20.2–25.4% HL, 22.9), a deeper snout (9.2–12% HL, mean = 10.6 vs. 6.6–9.2% HL, mean = 8), shorter gill rakers (middle gill raker: 6.8–11.3% HL, mean = 8.4 vs. 10.2–15% HL, mean = 12.6; longest gill raker: 8.9–12.4% Hl, mean = 10.1 vs. 11.2–17.7% HL, mean = 14.3) and less gill rakers (upper arch gill raker number: 7–9, mode = 9 vs. 11–15, mode =12; lower arch gill raker number: 14–18, mode = 15 vs. 21–26, mode = 22; total number of gill rakers: 21–27 vs. 33–40) (Tables 8, 13). Based on ratios *C.supersum* can be differentiated from *C.zugensis* by having a larger ‘caudal peduncle depth / dorsal head length’ ratio (CD/DHL: 0.46–0.58 vs. 0.37–0.44) (Table 11).

**Table 8.** Morphological and meristic data of historical specimen*s* of *Coregonussupersum*, *C.obliterus* and *C.zugensis* from Lake Zug and of *C.suidteri* from Lake Sempach. *Coregonussupersum*, NMBE-1076275, holotype; paratypes *N* = 5. *C.obliterus*, NMBE-1076268, holotype; paratypes *N* = 6. *C.zugensis*, syntypes *N* = 12. *C.suidteri*, syntypes, *N* = 2.

Lake	Zug									Sempach	
Species	*Coregonussupersum*			*Coregonusobliterus*			*Coregonuszugensis*			*Coregonussuidteri*	
	Holotype	Holotype + Paratypes		Holotype	Holotype + Paratypes		Lectotype	Lectotype + Paralectotypes		Syntypes	
		Ntotal = 6 *			Ntotal = 7 **			Ntotal= 12 ***		Ntotal= 2	
Morphological characters		mean	range		mean	range		mean	range	MHNG-715.089	MHNG-816.026
**Standard length (mm)**	288	299	263.5–411	282	272.9	250–288	205	198.5	174–236	311	312
**Percentage of standard length**											
Pelvic fin base	4.3	3.9	3.6–4.3	3.9	3.5	3–4	3.9	3.8	2.8–4.5	5	4.6
Pelvic fin ‘spine’ length	6.3	6.4	5.6–6.9	4.9	5.4	4.8–6.5	6.7	6.4	4.9–7.5	7	4.6
Pelvic fin length	17.2	15.8	14.6–17.9	16.1	16.3	15.4–17.7	16.5	17.2	15.7–19	18.9	16.2
Pectoral fin base	2.8	3.2	2.8–3.7	3.3	3.4	2.9–4	3.4	3.6	2.5–4.2	3.9	3.5
Pectoral fin 1 length	14.8	15.3	13.3–17.7	14.8	15.2	14.6–17	17.2	17	10.5–19.2	17.5	15.2
Pectoral fin 2 length	15.9	16.2	14.2–18.3	16.4	16.4	15.7–18	18.4	18.5	13.8–20.9	18.8	15.9
Dorsal fin base	11.6	12.1	10–14.5	10.8	11.8	10.8–13.7	10.7	11	9.5–12.8	11.3	11.7
Length of anterior part of dorsal fin erected	18.1	17.4	15.2–19.6	15.6	17.3	15.6–18.8	17.6	18.5	16.8–20.2	19.1	18.3
Length of anterior part of dorsal fin depressed	19	18.9	16.4–21.3	16.7	18.5	16.7–20.1	18.4	19.7	18.4–21.8	20.6	19.5
Length of posterior part of dorsal fin erected	4.5	5.2	4.4–6.2	5.1	5.4	4.7–6.1	6.2	6	5.5–7.2	7.3	5.8
Anal fin base	11	11.5	10.8–12	11.2	11.7	10.9–12.8	11.3	12.2	9.4–14.3	11.4	12.5
Length of anterior part of the anal fin	11.3	11.8	11.3–12.8	12.2	12.2	11.7–13	11.2	12.5	11.2–13.7	14.2	12.4
Adipose fin base	5.1	5.4	5–5.6	5.4	4.5	3–5.4	7.3	6	4.8–7.3	5	3.9
Caudal fin length	20.9	19.4	15.8–21.7	21.7	22.2	21.2–24.1	na	23	19.9–27.2	na	na
Caudal peduncle depth	7.2	7.2	6.7–8.1	6.7	7	6.6–7.4	7.2	7.2	6.4–8	8.7	7.8
Caudal peduncle length	14.4	13.1	12.2–14.4	12.9	13.7	12.1–16.4	13.1	13.8	12.5–14.8	13.3	13.5
Length from posterior part of adipose fin to caudal fin base	17.2	18.4	17.2–19.7	17.9	18.1	15.8–19.4	19.1	19.5	18.2–21.2	15.6	13.7
Dorsal head length	13.6	14.4	13.6–15.4	14.8	15.1	14.1–15.9	15.1	15.7	14.9–16.8	15.7	14.2
Prepelvic length	49.2	50.2	47.4–54.5	51.1	51	48.9–54.7	50.5	51.7	47.8–56.5	56.4	49.8
Preanal length	78.6	78.3	75.9–80.1	77.2	77.4	74.6–80.8	77.6	76.6	72.4–78.2	79.7	78.2
Predorsal length	48	47.3	46.4–48.6	48.6	47	44.1–48.6	46.7	47.4	45.4–50.2	50.2	49.5
Body depth	24.3	24.4	23.2–26.6	22.7	24.3	22.7–26.1	23	24	22.5–25.9	29	25.4
Postdorsal length	43.7	43.6	41.3–45.3	43.7	44.4	41.2–51.2	44.1	43.7	42.5–45.3	42.8	39.5
Head length	20.5	20.9	19.8–21.8	21.7	21.5	20.4–22.4	21.3	22	21.3–23.5	22	20
Total length	120	116.5	109.9–120	122	119.8	115.5–122	na	121.4	116.2–126	120	114
**Head length (mm)**	59.2	62.5	52.3–86.8	61.2	58.6	55.2–61.2	43.7	43.8	38.3–53.6	69.3	63.1
**Percentage of head length**											
Snout length	20.3	21	19.6–22.7	21.4	21.3	18.8–22.8	19.4	21.4	19.4–23.8	21.5	20.8
Eye diameter	19	19.9	18.2–21.7	24.7	23.2	21.5–24.7	22.3	22.9	19.7–25.3	21.9	23.9
Eye cavity	24.1	25.2	23.1–27.2	28.8	28.9	27.9–31	26.8	27.4	24.5–29	29.6	30
Eye height	19.4	20.9	19.4–22.2	24.5	23.9	23–25.2	23.6	23.9	21.7–26.7	23.6	23.7
Eye socket	5	4.5	2.6–5.1	2	4.5	2–6.5	3.9	3.8	1.6–5.9	6.5	4.5
Postorbital length	54.5	54.1	52.7–54.6	53.1	52.4	49.2–54.6	52.7	51.5	49.1–54.1	50.9	51.7
Head depth	65.9	70.3	65.2–81.9	68.4	70.4	65–74.7	71.4	69.3	64.6–73.6	78.7	74
Mouth width	9.2	9.3	8.4–10.5	8.9	9.4	8.7–10.5	9	9.5	6.7–11.3	11.1	10.3
Upper jaw length	25.7	26.5	24.3–28	27.3	26.6	22.4–28.7	26.7	30.4	26.7–33.3	30.1	27.8
Lower jaw length	36.5	37.9	36.3–41.6	39.5	38.5	37.1–42	43	42	38.4–44.6	41.5	42.2
Length of maxilla	18.5	18.8	17.8–21.3	18.4	19.6	17–21.7	20.2	22.9	20.2–25.4	21.3	23
Snout depth	10.5	10.6	9.2–12	9.1	10.1	8–13	6.6	8	6.6–9.2	10.2	9.9
Snout width	13.3	15.1	13.3–16.4	13.8	14.3	13.4–15.2	16.3	15.5	13.7–17.1	17	15.6
Head width	47.1	48.5	44.1–51.6	44.1	45.9	42.9–47.7	44.7	46	43.2–49	49.1	46.1
Interorbital width	26.9	27.5	25.8–29.7	28	28.4	26.4–29.7	24.2	25.3	23.3–27.1	25.4	19.7
Internarial width	11.6	12	10.7–13.9	14.1	13.2	10.7–14.1	12.2	11.9	10–13.5	15.4	13.8
Lower jaw length	7.7	8.1	7.2–9.2	6.9	8	6.9–8.8	8.6	8.4	7.4–9	12.3	11.7
Lower jaw width	22	23.1	20.5–25.6	22.4	23.4	21.8–25.1	20.1	22.9	20.1–25.6	24.6	24.2
Middle gill raker length	7.5	8.4	6.8–11.3	7.9	7.8	5.3–10.6	na	12.6	10.2–15	8.9	na
Longest gill raker length	10.2	10.1	8.9–12.4	8.3	8.8	7.6–10.6	na	14.3	11.2–17.7	9.1	na
Upper arch length	na	na	na	na	na	na	na	15.1	na	17.3	na
Lower arch length	na	na	na	na	na	na	na	29.7	na	30.9	na

* N=5 for CF, TL, MGR; LGR, UA, LA; ** N=11 for SL, PELVB, PELVFS, PELVF, PECFB, PECF1, PECF2, DFB, DFPe, DFPd, DFAe, AFB, AFPe, AdFB, CD, CL, PAdC, DHL, PreP, PreA, PreD, BD, PostD, HL, MGR, LGR; N=10 for CF; N=8 for TL; N=1 for UA, LA; ** N=6 for DFPe, DFPd, AFPe, TL, LJW; N=5 for CF; *** N=11 for SL, PELVB, PELVFS, PELVF, PECFB, PECF1, PECF2, DFB, DFPe, DFPd, DFAe, AFB, AFPe, AdFB, CD, CL, PAdC, DHL, PreP, PreA, PreD, BD, PostD, HL, MGR, LGR; N=10 for CF; N=8 for TL; N=1 for UA, LA.

*Coregonussupersum* – *Coregonusobliterus*

*Coregonussupersum* can be differentiated from *C.obliterus* by having a shorter caudal fin (15.8–21.7% SL, mean = 19.4 vs. 21.2–24.1% SL, mean = 22.2), a smaller eye (eye diameter: 18.2–21.7% HL, mean = 19.9 vs. 21.5–24.7% HL, mean = 23.2; eye cavity: 23.1–27.2, mean = 25.2 vs. 27.9–31% HL, mean = 28.9; eye height: 19.4–22.2% HL, mean = 20.9 vs. 23–25.2% HL, mean = 23.9) and longer longest gill raker (8.9–12.4% HL, mean = 10.1 vs. 7.6–10.6% HL, mean = 8.8) (Table 8). Based on ratios *C.supersum* can be differentiated from *C.obliterus* by having a larger ‘predorsal length / eye height’ ratio (PreD/EH: 10.52–12.07 vs. 8.46–9.73) and a larger ‘adipose fin base / eye depth’ ratio (AdFB/ED: 1.15–1.42 vs. 0.64–1) (Table 11).

*Coregonussupersum* – *Coregonuslitoralis*

*Coregonussupersum* can be differentiated from *C.litoralis* by having a shorter dorsal head length (13.6–15.4% SL, mean = 14.4 vs. 14.4–17.9% SL, mean = 15.2), a shorter middle gill raker (6.8–11.3% HL, mean = 8.4 vs. 8.9–13.4% HL, mean = 11.1) and by having a larger ‘mouth width / snout depth’ ratio (MW/SD: 1–1.21 vs. 0.75–1) (Tables 2, 8, 11).

*Coregonussupersum* – *Coregonussuidteri*

*Coregonussupersum* can be differentiated from *C.suidteri* by having a shallower pelvic fin base (3.6–4.3% SL, mean = 3.9 vs. 4.6–4.9% SL, mean = 4.7), a shallower pectoral fin base (2.8–3.7% SL, mean = 3.2 vs. 3.5–3.9% SL, mean = 3.7), a shorter erected anterior anal fin (11.3–12.8% SL, mean = 11.8 vs. 12.4–14.2% SL, mean = 13.3), a longer distance from the posterior part of the adipose fin to the caudal fin base (17.2–19.7% SL, mean = 18.4 vs. 13.7–15.6% SL, mean = 14.6), a shorter pre-dorsal distance (46.4–48.6% SL, mean = 47.3 vs. 49.5–50.2% SL, mean = 49.8), a smaller eye (eye diameter: 18.2–21.7% HL, mean = 19.9 vs. 21.9–23.9% HL, mean = 22.9; eye cavity: 23.1–27.2% HL, mean = 25.2 vs. 29.6–30% HL, mean = 29.8; 19.4–22.2% HL, mean = 20.9 vs. 23.6–23.7% HL, mean = 23.7), a less wide and shorter mouth (mouth width: 8.4–10.5% HL, mean = 9.3 vs. 10.3–11.1% HL, mean = 10.7; upper jaw length: 24.3–28% HL, mean = 26.5 vs. 27.8–30.1% HL, mean = 28.9; lower jaw length: 36.3–41.6% HL, mean = 37.9 vs. 41.5–42.2% HL, mean = 41.9; maxilla: 17.8–21.3% HL, mean = 18.8 vs. 21.3–23% HL, mean = 22.1), a wider interorbital width (25.8–29.7% HL, mean = 27.5 vs. 19.7–25.4% HL, mean = 22.6), a less wide internarial width (10.7–13.9% HL, mean = 12 vs. 13.8–15.4% HL, mean = 14.6) and lower jaw width (7.2–9.2% HL, mean = 8.1 vs. 11.7–12.3% HL, mean = 12) (Table 8).

###### Distribution and notes on biology.

*Coregonussupersum* occurs in Lake Zug and it has been shown with genetic analysis (Hudson et al. 2011) that translocated populations of whitefish in Lake Maggiore (local name of the population is ‘Lavarello’) and Lake Lugano group in a neighbour-joining tree with the extant population of whitefish from Lake Zug, suggesting that *C.supersum* also occurs in these two lakes south of the alps. *Coregonussupersum* is most likely the only species out of three species that did not go extinct in Lake Zug (this is discussed in more detail in the Suppl. material 1: paragraph 4). Lake Zug used to harbour three whitefish species, of which two are extinct, *C.zugensis* and *C.obliterus*. The only extant whitefish species of Lake Zug is *C.supersum*. The extinction of *C.obliterus* and *C.zugensis* can be attributed to strong anthropogenic-induced eutrophication that was present in many Swiss lakes, which was accompanied by population collapse, speciation reversal, and extinction of Swiss whitefish (Vonlanthen et al. 2012; Frei et al. 2022a, b). Steinmann (1950) notes that *C.supersum* feeds on zooplankton and benthic prey items. A habitat-stratified fishing campaign in Lake Zug during a short time window in summer showed that the whitefish population of Lake Zug only occupies the first 20 metres of the pelagic and benthic water column, despite Lake Zug being almost 200 metres deep; below 81 metres no fish were caught, which is attributed to anoxic conditions in the hypolimnion (Vonlanthen et al. 2015). The spawning season of *C.supersum* used to take place in the last few weeks of December (Steinmann 1950), but in recent years this has shifted to the first weeks of January (P. Reichlin, pers. comm.). *Coregonussupersum* spawns in depths of ~ 10–40 m (Steinmann 1950; P. Reichlin, pers. comm.).

###### Etymology.

The specific name *supersum* means in Latin to be ‘left over’ or to have ‘survived’. The name refers to the fact that this is the only species to exist to date in Lake Zug. A verb.

###### Common name.

We suggest the German name Zugerbalchen.

##### ﻿ Coregonus zugensis

Taxon classificationAnimaliaSalmoniformesCoregonidae

Nüsslin, 1882

1342D46E-B382-52D2-BD38-B3FB2BDC746E

**Figure 10.**
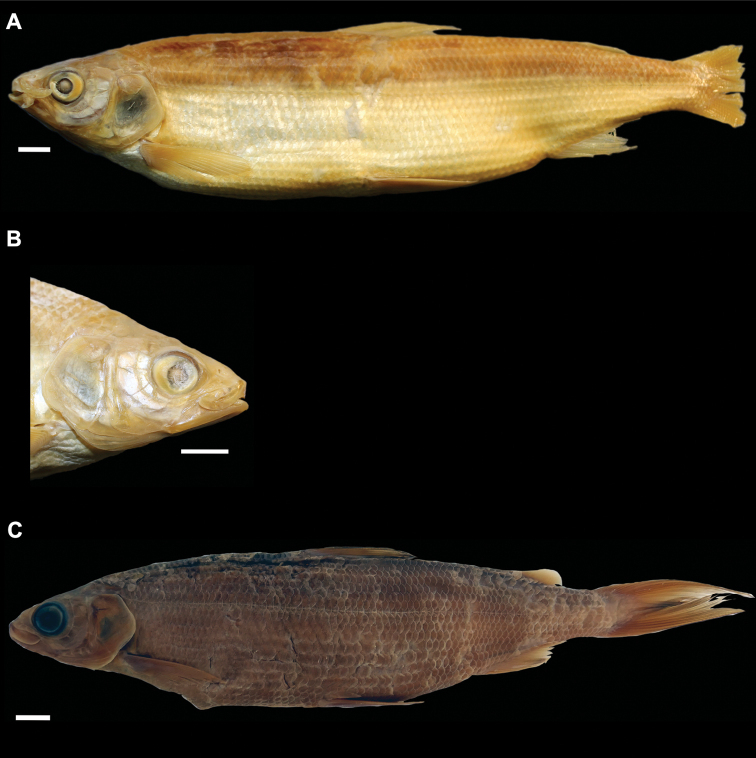
*Coregonuszugensis*, Lake Zug, Switzerland **A, B** syntype, MHNG-715.093, 205 mm SL, sex unknown, preserved specimen **C** syntype, NMBE-1076275 (Steinmann-290-2), 188 mm SL, female, preserved specimen. Scale bars: 1 cm.

Figs 10 , 14 , Tables 8 , 10 , 11 , 12 , 13 , 14 , Suppl. material 1: tables S1, S2

Coregonus sp. ‘Albeli’: Fatio 1885.
Coregonus sp. ‘Albeli-Albock’: Fatio 1890 (see also synonymy of C.obliterus).
Coregonus crassirostris compactus : Fatio 1885 (see also synonymy of C.supersum).
Coregonus lavaretus nat. *riusensis*, oekot. *primigenius nanus*: Steinmann 1950 (see also synonymy of C.muelleri and C.sarnensis).
Coregonus macrophthalmus var. zugensis : Nüsslin 1882.
Coregonus wartmanni compactus : Fatio 1890 (see also synonymy of C.obliterus).
Coregonus sp. ‘Zugeralbeli’: Vonlanthen et al. 2012, 2015.
Coregonus ‘Zugerseealbeli’: Steinmann 1950.

###### Material examined.

***Syntypes*.** All from Switzerland, Lake Zug: Historical specimens (years 1879, 1890, 1937, 1939): MHNG-715.093, NMBE-1076263 (EAWAG-279-1, EAWAG279-2), NMBE-1076264 (EAWAG-280-1, EAWAG-280-2), NMBE-1076266 (EAWAG-282-1, EAWAG-282-2), NMBE-1076275 (EAWAG-290-2, EAWAG-290-3, EAWAG-290-3, EAWAG-290-4, EAWAG-290-5, EAWAG-290-6), *N* = 12, 173–236mm SL.

###### Diagnosis.

*Coregonuszugensis* is a small whitefish species with weak pigmentation of all fins and body; pale olive-brown colouration on the flanks above the lateral line; elongate slender body (body depth: 22.5–25.9% SL, mean = 24); large eye (eye diameter: 19.7–25.3% SL, mean = 22.9) with a subtle triangular eye socket (1.6–5.9% HL, mean = 3.8); many and long gill rakers (longest gill raker: 11.2–17.7% HL, mean = 14.3; total gill raker number: 33–40).

###### Description.

***Shape***: Body elongated and slender. Greatest body depth anterior of the dorsal fin. Ventral profile and dorsal profile similar and slightly arched. Dorsal and ventral profile from tip of snout to interorbital mostly straight and then slightly convex to dorsal and pelvic fin origin respectively. Head long. Snout long and tip of snout if slightly pointed resulting in a not strongly pronounced rostral plate. Mouth long and terminal. Large eye with a subtle sickle cell-shaped (seldom roundish) eye-socket. Pectoral fin long and tapered. Caudal peduncle moderately stout. ***Meristics***: Many and long gill rakers. ***Colour***: Pigmentation of fins and body weak in live specimens. Pectoral fin transparent and pelvic and anal fin mostly transparent. Pectoral fin very rarely pigmented at the distal part of the fin and the anal and pelvic fin rarely moderately pigmented at the median to distal parts of the fin. Dorsal, caudal and adipose fin moderately pigmented. Silvery appearance along the flanks. Dorsally above the lateral line the silvery appearance changes to a pale olive-brown. Pre-operculum and operculum with one black spot on the lower margin of the pre-operculum. Preserved specimens are brownish in colouration.

###### Differential diagnosis.

The differential diagnoses against the historical specimens of *C.supersum* from Lake Zug are given under that species account.

*Coregonuszugensis* – *Coregonusobliterus*

*Coregonuszugensis* can be differentiated from *C.obliterus* by having a wider adipose fin base (4.8–7.3% SL, mean = 6 vs. 3–5.4% SL, mean = 4.5), a smaller eye cavity (24.5–29% HL, mean = 27.4 vs. 27.9–31% HL, mean = 28.9), a longer maxilla (20.2–25.4% HL, mean = 22.9 vs. 17–21.7% HL, mean = 19.6), a shallower snout (6.6–9.2% HL, mean = 8 vs. 8–13% HL, mean = 10.1), a less wide interorbital (23.3–27.1% HL, mean = 25.3 vs. 26.4–29.7% HL, mean = 28.4) and longer and more gill rakers (middle gill raker: 10.2–15% HL, mean = 12.6 vs. 5.3–10.6% HL, mean = 7.8; longest gill raker: 11.2–17.7% HL, mean = 14.3 vs. 7.6–10.6% HL, mean = 8.8; upper arch gill raker number: 11–15, mode = 12 vs. 7–9, mode = 9; lower arch gill raker number: 21–26, mode = 22 vs. 13–18, mode= 14; total gill raker number: 33–40, mode = 35, 37 vs. 21–26, mode = 25). Based on ratios *C.zugensis* can be differentiated from *C.obliterus* by having a smaller ‘eye diameter / maxilla’ ratio (EC/M: 1.11–1.34 vs. 1.38–1.65) (Tables 8, 11, 13).

*Coregonuszugensis* – *Coregonusmuelleri*

*Coregonuszugensis* can be differentiated from *C.muelleri* by having a deeper caudal peduncle (6.4–8% SL, mean = 7.2 vs. 6.1–6.8% SL, mean = 6.5), a smaller eye cavity (24.5–29% HL, mean = 27.4 vs. 27–31.9% HL, mean = 29.2) and a deeper head (64.6–73.6% HL mean = 69.3 vs. 61.8–69.7% HL, mean = 65.2). Based on ratios *C.zugensis* can be differentiated from *C.muelleri* by having a smaller ‘caudal peduncle depth / prepelvic length’ ratio (CD/PreP: 0.13–0.15 vs. 0.12–0.13) (Tables 6, 8, 11).

###### Distribution and notes on biology.

*Coregonuszugensis* occurred in Lake Zug and is extinct today (this is discussed in more detail in the Suppl. material 1: paragraph 4). Fatio (1885) mentions in a table three whitefish species for lake Zug ‘Balchen’, ‘Albock’, and ‘Albeli’, but later Fatio (1890) only mentions two whitefish species for Lake Zug, namely the ‘Balchen’ (Coregonusschinziihelveticusvar.zugensis) and the ‘Albeli-Albock’ (*Coregonuswartmannicompactus*). Interestingly, Wagler (1937) noted two species to be present in Lake Zug with a remark that a third species may exist, but that its status is uncertain. Fatio (1890) does mention that fishermen have suggested that among the ‘Albeli-Albock’ there are individuals that are smaller and spawn later in the year, which they refer to as ‘Albeli’, when compared to the individuals they refer to as ‘Albock’ that are slightly larger and spawn earlier in the year. However, the specimens that Fatio (1890) could examine did not warrant this distinction and thus he suggested to group the ‘Albeli’ and ‘Albock’ under the name ‘Albeli-Albock’. Fatio (1890) suggested that the ‘Albeli-Albock’ spawn in mid-September to mid-October in Lake Zug at depths of 150–180 metres. However, with the findings that Lake Zug did harbour three species of whitefish (see the notes on biology of *C.supersum* and the Suppl. material 1: paragraph 4 for details) and taking into account the spawning Table in Fatio (1885) and the reports by local fishermen from that time period (Fatio 1885, 1890) we suggest that the ‘Albeli’, *C.zugensis*, spawned in comparison to *C.obliterus* (‘Albock’) later in the year from mid-October to late December in deep waters of the lake. Fatio (1885) states that the ‘Albeli’, *C.zugensis*, spawns in the deeper parts of the lake (‘fond’ in French) and that the ‘Albock’, *C.obliterus*, spawns in even deeper waters of the lake (‘grand fond’ in French). Fatio (1890) groups the ‘Albeli-Albock’ under *Coregonuswartmannicompactus*, which includes several other small and pelagic whitefish species from other lakes. This and the number and length of gill rakers of *C.zugensis* (many and long gill rakers) suggests, based on the functional properties of the number of gill rakers experimentally tested with specimens of other whitefish species from lakes Thun and Lucerne (Lundsgaard-Hansen et al. 2013; Roesch et al. 2013), that *C.zugensis* was feeding predominantly on zooplankton.

###### Etymology.

The specific name zugensis refers to Zug, a city that gave its name to Lake Zug. An adjective.

###### Common name.

Zugeralbeli.

##### ﻿ Coregonus obliterus sp. nov.

Taxon classificationAnimaliaSalmoniformesCoregonidae

9BEBB939-1F48-5C8D-A3FA-B204E567730D

https://zoobank.org/F466C3E4-459C-41C7-9940-29A33293FED0

**Figure 11.**
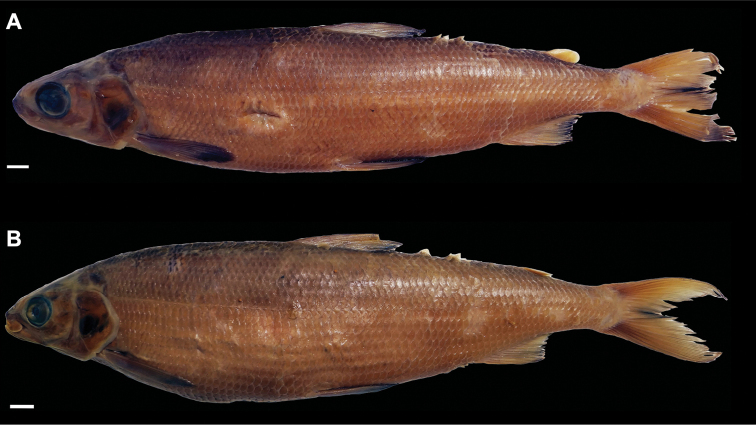
*Coregonusobliterus*, Lake Zug, Switzerland **A** holotype, NMBE-1076268, 281mm SL, male, preserved specimen **B** paratype, NMBE-1076268, 276 mm SL, female, preserved specimen. Scale bars: 1 cm.

Figs 11 , 14 , Tables 8 , 10 , 11 , 12 , 13 , 14

Coregonus sp. ‘Albock’: Fatio 1885.
Coregonus sp. ‘Albeli-Albock’: Fatio 1890 (see also synonymy of C.zugensis).
Coregonus lavaretus nat. *riusensis*, oekot. *primigenius nanus*: Steinmann 1950 (see also synonymy of C.muelleri and C.sarnensis).
Coregonus wartmanni compactus : Fatio 1890 (see also synonymy of C.zugensis).

###### Material examined.

***Holotype*.** Historical specimen (year 1937): NMBE-1076268 (EAWAG-284-1), 281 mm SL, sex male; Switzerland: Lake Zug. ***Paratypes*.** All from Switzerland, Lake Zug: Historical specimens (years 1937, 1939): NMBE-1076268 (EAWAG-284-2, EAWAG-284-4), NMBE-1076271, NMBE-1076276, NMBE-1076906 (EAWAG-956-1, EAWAG-956-2), *N* = 6, 250–288 mm SL.

###### Diagnosis.

*Coregonusobliterus* is a small whitefish species with moderate pigmentation of all fins and body; elongated slender body (body depth: 22.7–26.1% SL, mean = 24.3); short mouth (length of maxilla: 17–21.7% HL, mean = 19.6); pronounced rostral plate; very large eye (eye diameter: 21.5–24.7% HL, mean = 23.2) with a subtle triangular (seldom roundish) eye socket; few and very short gill rakers (longest gill raker: 7.6–10.6% HL, mean = 8.8; total gill raker number: 21–26).

###### Description.

***Shape***: Body elongated and slender. Greatest body depth anterior of the dorsal fin. Ventral profile and dorsal profile similar and slightly arched. Dorsal and ventral profile from tip of snout to interorbital mostly straight and then slightly convex to dorsal and pelvic fin origin respectively. Head long. Snout often 40–50° angle to the body axis anterior of the eye, such that the profile from the tip of the snout to the vertical projection where the anterior part of the eye crosses the dorsal profile is straight and afterwards slightly convex. Snout moderately long and tip of snout quite deep with a strongly pronounced rostral plate. Mouth short and terminal to sub-terminal. Very large eye with a subtle sickle cell-shaped (seldom roundish) eye-socket. Pectoral fin short and tapered. Caudal peduncle moderately stout. ***Meristics***: Few and very short gill rakers. ***Colour***: Pigmentation of fins and body moderate in preserved specimens. All fins moderately pigmented at the median to distal parts of the fin. Operculum with one black spot on the lower margin of the pre-operculum. Preserved specimens are brownish in colouration.

###### Differential diagnosis.

The differential diagnoses against the historical specimens of *C.supersum* and *C.zugensis* from Lake Zug are given under those species’ accounts.

###### Distribution and notes on biology.

*Coregonusobliterus* occurred in Lake Zug and is extinct today (this is discussed in more detail in the Suppl. material 1: paragraph 4). Fatio (1885) mentions three whitefish species for lake Zug, ‘Balchen’, ‘Albock’, and ‘Albeli’, but later Fatio (1890) only mentions two whitefish species for Lake Zug, namely the ‘Balchen’ (Coregonusschinziihelveticusvar.zugensis) and the ‘Albeli-Albock’ (*Coregonuswartmannicompactus*). Interestingly, Wagler (1937) noted two species to be present in Lake Zug with a remark that a third species may exist, but that it’s status is uncertain. Fatio (1890) does mention that fishermen have suggested that among the ‘Albeli-Albock’ there are individuals that are smaller and spawn later in the year, which they refer to as ‘Albeli’, when compared to the individuals they refer to as ‘Albock’ that are slightly larger and spawn earlier in the year. However, the specimens that Fatio (1890) could examine did not warrant this distinction and thus he suggested to group the two entities under the name ‘Albeli-Albock’. Fatio (1890) suggest the the ‘Albeli-Albock’ spawn in mid-September to mid-October in Lake Zug at depths of 150–180 metres. In the table Fatio (1885: table 1) states that the ‘Albeli’, *C.zugensis*, spawns in the deeper parts of the lake (‘fond’ in French) and that the ‘Albock’, *C.obliterus*, spawns in even deeper waters of the lake (‘grand fond’ in French). Based on the findings that Lake Zug did harbour three species of whitefish and taking into account the spawning table and notes referring to the table in Fatio (1885) and the reports by local fishermen from that time period (Fatio 1885, 1890) we suggest that this spawning period and depth should be accounted for ‘Albock’, *C.obliterus*. It seems based on the phenotype of *C.obliterus* including the spawning time and depth and the few and short gill rakers of this species, that this species must have occupied a similar ecological niche as is known for two whitefish species from two other lakes in Switzerland. *C.gutturosus* (today extinct) from Lake Constance and *C.profundus* from Lake Thun share many ecological and morphological characters with *C.obliterus*. More is known about the biology of these two species; *C.gutturosus* was (extinct today) and *C.profundus* is a profundal specialist feeding predominantly on benthic prey items and living and spawning in great water depths.

###### Etymology.

The name in Latin *obliterus* means ‘erased from memory’ or ‘forgotten’. The name highlights that this species was forgotten for more than a century in the scientific literature. An adjective.

###### Common name.

We suggest the German name Zugeralbock.

#### Lake Sempach whitefish

##### ﻿ Coregonus suidteri

Taxon classificationAnimaliaSalmoniformesCoregonidae

Fatio, 1885

6A3D31B3-779E-52A9-9F77-C9DDF814A9E1

**Figure 12.**
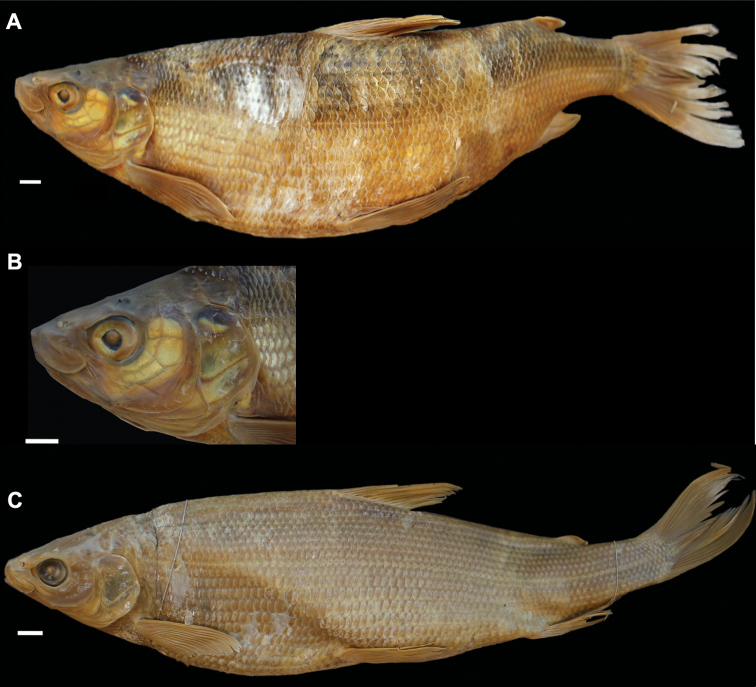
*Coregonussuidteri*, Lake Sempach, Switzerland **A, B** syntype, MHNG-715.089, 311 mm SL, sex unknown, preserved specimen **C** syntype, MHNG-816.026, 312 mm SL, sex unknown, preserved specimen. Scale bars: 1 cm.

Figs 12 , 14 , Tables 8 , 10 , 12 , 13 , 14 , Suppl. material 1: figs S2, S3, tables S1, S2

Coregonus annectus : Fatio 1885.
Coregonus annectus balleoides : Fatio 1885.
Coregonus lavaretus nat. *riusensis*, oekot. Primigenius: Steinmann 1950 (see also synonymy of C.sarnensis, C.supersum, and C.litoralis).
Coregonus Schinzii helveticus var. zugensis : Fatio 1890.
Coregonus Schinzii helveticus var. lucernensis : Fatio 1890.
Coregonus ‘Sempacherballen’: Douglas and Brunner 2002.
Coregonus suidteri : Kottelat 1997; Kottelat and Freyhof 2007; Vonlanthen et al. 2011, 2015 (see also synonymy of C.litoralis and C.supersum).
Coregonus lavaretus fera natio vogti: Berg 1932.
Coregonus lavaretus natio riusensis: Steinmann 1950.

###### Material examined.

***Syntypes*.** All from Switzerland, Lake Sempach: Historical specimens (years 1885, 1899): MHNG-715.089, MHNG-816.026, *N* = 2, 311 mm and 312 mm SL, sex unknown; MHNG-676.007, branchial arch, which probably belongs to one of the syntypes (used for gill raker measurements).

###### Diagnosis.

*Coregonussuidteri* is a large whitefish species with strong pigmentation of all fins and the body; greenish blue colour on the flanks above the lateral line; deep bodied (body depth: 25.4 and 29% SL); blunt snout; short head (20 and 22% SL); sub-terminal mouth; moderately large eye (eye diameter: 21.9 and 23.9% HL) with a thick (4.5 and 6.5% HL) and triangular-shaped eye socket; short and stout caudal peduncle (caudal peduncle depth: 7.8 and 8.7% SL; caudal peduncle length: 13.3 and 13.5% SL); many but rather short gill rakers (longest gill raker: 9.1% HL; total gill raker number: 35).

###### Description.

***Shape***: Generally, deep bodied with greatest body depth anterior of dorsal fin. Dorsal profile from tip of snout to anterior origin of dorsal fin strongly convex and ventral profile moderately convex or almost straight from interorbital area to pelvic fin origin. Head short. Mouth short, wide and sub-terminal. Rostral plate pronounced. Tip of snout blunt. Large eye with a thick and sickle cell-shaped eye-socket. Pectoral fin moderately tapered and short. Dorsal fin long. Caudal peduncle stout and short. ***Meristics***: Many but rather short gill rakers. ***Colour***: Pigmentation of fins and body overall strong with a silvery appearance along the flanks and dorsally above the lateral line the silvery appearance changes to a greenish blue colour (based on preserved specimens and by the description by Fatio (1890)). The dorsal part of the head and the snout around the nostrils is strongly pigmented. Preserved fish are brownish in colouration.

###### Differential diagnosis.

The differential diagnoses against the historical specimens of *C.supersum* from Lake Zug are given under that species account.

*Coregonussuidteri* – *Coregonuslitoralis*

*Coregonussuidteri* can be differentiated from *C.litoralis* by having a shorter length from the posterior part of the adipose fin to the caudal fin base (13.7–15.6% SL, mean = 14.6 vs. 15.8–22.9% SL, mean = 19.3), shorter snout (20.8–21.5% HL, mean = 21.1 vs. 19–29% HL, mean = 23.4), a larger eye cavity (29.6–30% HL, mean = 29.8 vs. 24.1–27.7% HL, mean = 26), a shorter postorbital length (50.9–51.7% HL, mean = 51.3 vs. 51.6–56.2% HL, mean 53.5), a deeper head (74–78.7% HL, mean = 76.4 vs. 68.1–77.1% HL, mean = 72.5), a less wide interorbital width (19.7–25.4% HL, mean = 22.6 vs. 23.5–31.3% HL, mean = 28.1), a wider internarial width (13.8–15.4% HL, mean = 14.6 vs. 10.7–14.5% HL, mean = 12.9) and a wider lower jaw (11.7–12.3% HL, mean = 12 vs. 6.9–9.2% HL, mean = 8.1) (Tables 2, 8).

###### Distribution and notes on biology.

*Coregonussuidteri* is found in Lake Sempach. *Coregonussuidteri* was previously thought to naturally occur in several lakes in Switzerland, namely lakes Lucerne, Zug, Sempach, Hallwil and Baldegg. Independent multilocus microsatellite (Suppl. material 1: figs S2, S3) and large genomic AFLP (Hudson et al. 2011) data sets have shown that *C.suidteri* is composed of multiple species endemic to different lakes in Switzerland. Both population-based neighbour-joining tree’s and individual-based population structure analysis suggest that contemporary samples of whitefish from Lake Zug, Sempach and Lucerne group into independent genetic clusters (Suppl. material 1: figs S2, S3; Hudson et al. 2011). The status of the extinct species C.cf.suidteri from lakes Hallwil and Baldegg is unresolved since no genetic material is present to date. The extant population of Lake Sempach whitefish on which these independent multilocus microsatellite and large genomic AFLP analyses are based on, show strong signals of genetic association with whitefish from Lake Zug and Lake Lucerne. As has been noted before for the other lakes in this study (and many more lakes in Switzerland) historical records report several incidences of introductions of whitefish from other Swiss, German and even North American lakes into Lake Sempach (Surbeck 1920; Steinmann 1950). Surbeck (1920) and Steinmann (1950) note that in the years from 1895–1902 there were several introductions of ‘Balchen’ (most likely *C.litoralis* and/or *C.intermundia*) from Lake Lucerne into Lake Sempach. Steinmann (1950) further notes that other species of whitefish from many lakes were also introduced, but then only specifically mentions *C.maraena* and whitefish individuals from North America. Most likely many different whitefish species from Swiss lakes and beyond were introduced. This is not unique to Lake Sempach and was unfortunately common practice at that time. These deliberate introductions from other lakes took place after the population decline of whitefish in Lake Sempach, which were attributed to the lowering of the lake level in the 19^th^ century (Steinmann 1950). Prior to this population decline in 19^th^ century Lake Sempach seemed to harbour a large and productive whitefish fishery according to fisheries catch accountings (Steinmann 1950). Interestingly though, the catches of whitefish of the years 1418–1795 reported that the whitefish species in those times were rather small individuals (125 grams) compared to the larger individuals of the late 19^th^ century (375–750 grams) which led Steinmann (1950) to suggest that Lake Sempach may have harboured more than one species of whitefish. Fatio (1890) also notes that the size of the whitefish species in Lake Sempach were smaller and increased with the population decline towards the end of the 19^th^ century. Steinmann (1950) named the small type whitefish of Lake Sempach ‘Kleiner Balchen des Sempachersees’ and the large-type whitefish species ‘Sempacherbalchen’. It is the latter species that is most likely represented by the type specimens of *C.suidteri* that were given to Victor Fatio in the 1880s by Otto Suidter (Steinmann 1950). Furthermore Steinmann (1950) suggests that the ‘Sempacherbalchen’ (i.e., *C.suidteri*), which he could examine in the last half of the 20^th^ century, should be regarded as a ‘mixtum compositum’, i.e., a mixed population composed of different whitefish species including the original large-type whitefish species of Lake Sempach. Indeed, Fatio (1890) already questioned if the small whitefish individuals from Lake Sempach would have had to be described as an independent species, which was displaced by larger individuals of a less common second whitefish species after the decline of the former. Given these uncertainties expressed both by Fatio (1890) and Steinmann (1950) regarding the number of species that were initially present in Lake Sempach, coupled with 1) a population decline in the 19^th^ century of smaller individuals of a putative small-type species resulting in fewer but larger individuals of a second large-type species, 2) deliberate introductions in the late 19^th^ and early 20^th^ century, and 3) a strong anthropogenic-induced eutrophication during the last half of the 20^th^ century in Lake Sempach, which may have been accompanied (as has been shown for other lakes) by population collapse, speciation reversal, and extinction (Vonlanthen et al. 2012; Frei et al. 2022a, b), it has to be considered that the extant population of *C.suidteri* does not represent the original population of this species in Lake Sempach and thus should be considered extinct. Future work comparing individuals of the extant population of Lake Sempach with ancient DNA samples from sediment cores or museums samples may help to resolve if the extant population still carries a genetic legacy from the original species. We suggest that the extant population of whitefish in Lake Sempach are most likely a hybrid population with a possible genetic legacy of the original population of *C.suidteri* from Lake Sempach. According to Fatio (1885, 1890) *C.suidteri* spawned rather shallow in the month of November.

###### Common names.

Sempacherfelchen, Sempacherbalchen.

###### Comparative material.

**Figure 13.**
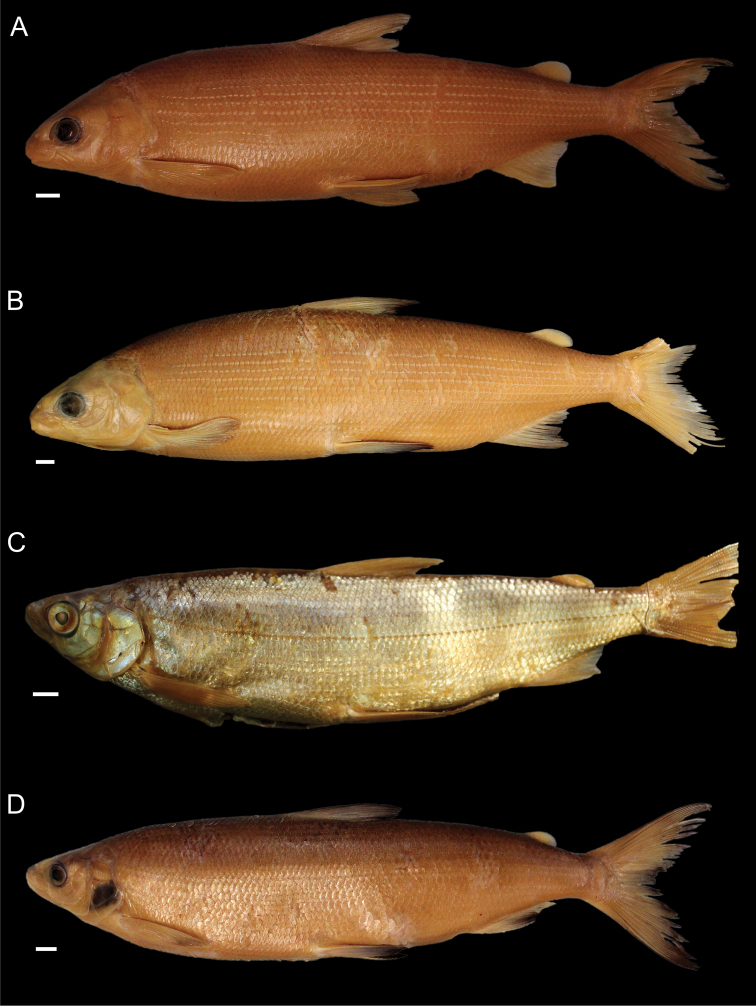
*Coregonus* species of Lake Constance, Switzerland **A***Coregonusgutturosus*, non-type, NMBE-1076232 (Eawag-248-1), 250 mm, sex unknown, preserved specimen **B***Coregonusarenicolus*, holotype, 296 mm, NMBE-1076223 (Eawag-239-1), sex unknown preserved specimen **C***Coregonusmacrophthalmus*, syntype, MHNG-716.052, 215 mm, sex unknown, preserved specimen **D***Coregonuswartmanni*, non-type, NMBE-1076206, 301 mm, female, preserved specimen. Scale bars: 1 cm.

All the fish detailed below were collected from Switzerland, Lake Constance. They are illustrated in Fig. 13 and listed in Tables 9, 12–14.

**Table 9.** Morphological and meristic data of *Coregonusgutturosus*, *C.arenicolus*, *C.macrophthalmus* and *C.wartmanni* from Lake Constance. *Coregonusgutturosus*, non-types *N* = 10. *Coregonusarenicolus*, holotype, NMBE-1076223 (Eawag-239-1), sex unknown; paratypes *N* = 3. *Coregonusmacrophthalmus*, syntypes *N* = 7. *C.wartmanni*, non-type, NMBE-1076206, female.

Species	*Coregonusgutturosus*		*Coregonusarenicolus*			*Coregonusmacrophthalmus*		*Coregonuswartmanni*
Morphological characters	Non-types N = 10 *		Holotype	Paratypes N = 3 **		Syntypes N = 7 ***		Non-type
	mean	range		mean	range	mean	Range	
**Standard length (mm)**	220.4	169–292	296.0	301.3	289–314	213.9	193–235	301
**Percentage of standard length**								
Pelvic fin base	4.1	3.7–4.4	3.9	4.4	3.9–4.6	3.8	3.3–4.2	3.8
Pelvic fin ‘spine’ length	6.1	5.3–6.7	5.4	5.7	5.2–6.1	5.7	4.8–6.9	6.5
Pelvic fin length	17.1	15.4–19.1	17.3	17.3	16.8–18.1	16.5	15.2–17.6	15.4
Pectoral fin base	3.4	2.9–3.9	3.3	3.4	3.2–3.5	3.2	2.8–3.9	3
Pectoral fin 1 length	16.8	14.8–18.9	14.4	16.8	14.4–17.2	16.4	15.1–18.1	16
Pectoral fin 2 length	18.2	16.8–20.3	15.7	17.4	15.7–18	17.1	15.6–18.4	17
Dorsal fin base	11.9	10.7–12.8	12.2	12.2	11.0–13.1	11.6	10.8–12.4	11.2
Length of anterior part of dorsal fin erected	19.3	17.6–21.6	18.9	19.2	18.0–20.3	18.2	16.6–19.6	16.6
Length of anterior part of dorsal fin depressed	20.4	19.0–22.2	20.2	20.5	19.3–21.9	19.2	17.2–20.5	18.2
Length of posterior part of dorsal fin erected	5.5	4.8–7.0	5.2	5.5	5.2–5.7	5.2	4.4–5.9	4.6
Anal fin base	12.4	11.4–13.4	11.9	11.5	10.7–12.7	12.3	10.6–14.2	12.5
Length of anterior part of the anal fin	12.3	10.7–13.9	13.2	13.3	12.9–13.8	12.1	10.8–13.9	11.1
Adipose fin base	5.6	4.9–6.1	5.7	5	3.7–6.2	5.3	4.9–5.8	4
Caudal fin length	23.2	20.8–25.6	na	24	24–24.1	22.6	21.8–24	23.8
Caudal peduncle depth	7.4	6.7–8.2	7.7	8.1	7.7–8.2	7.4	6.9–8	7.4
Caudal peduncle length	12.9	11.5–13.9	14.4	12.9	12.0–14.4	13.8	12.4–16.5	13.2
Length from posterior part of adipose fin to caudal fin base	18.5	17.4–19.3	19.6	17.2	14.6–19.6	18.9	17.6–20.2	17.8
Dorsal head length	16.8	15.4–18.1	15.1	15.1	14.8–15.3	15.7	14.4–16.5	14.5
Prepelvic length	52.7	50.4–54.1	49.5	50.6	49.5–51.0	51.7	48.1–53.1	50.7
Preanal length	77.9	76.0–80.4	75.0	79.2	75.0–80.3	76.8	75.7–78.3	77.4
Predorsal length	48.4	46.8–49.6	47.9	49.2	47.9–49.6	47	45.8–48.5	47.3
Body depth	25.9	22.9–29.6	24.4	26.2	24.4–27.1	23.5	21.0–26.9	23.5
Postdorsal length	43	40.6–45.4	44.8	42.2	40.4–44.8	43	41.6–45.7	44
Head length	22.9	21.4–24.6	20.9	21	20.6–21.4	22.2	21.8–23.5	21.5
Total length	120.2	115.1–124.3	na	121.5	119.4–123.5	119.2	118.9–120	120.6
**Head length (mm)**	50.3	41.6–62.4	61.8	63.7	59.6–67.2	47.5	42.6–51.3	64.7
**Percentage of head length**								
Snout length	22.4	21.1–23.1	23.4	23.4	21.6–24.6	21.7	18–25.6	24
Eye diameter	21.1	19.4–23	19.6	17.7	17.3–19.6	24.1	21.3–26.1	18.9
Eye cavity	26.9	25.4–29.3	25.7	25	24.1–25.7	28.9	25.4–30.8	23.9
Eye height	21.3	20.5–22.6	20.8	19.6	18.8–20.8	23.2	19.5–25.6	19
Eye socket	4.8	3.5–5.6	4.9	5.2	4.6–5.5	3.9	2.7–4.6	5.1
Postorbital length	52.8	51.5–54.4	55.7	54	53–55.7	50.2	48.5–53.2	53.4
Head depth	74.2	69.9–80.6	68.2	72.6	68.2–75	68.6	61.6–76.3	67.6
Mouth width	9.8	9–11.2	10.4	10.5	10–11	10.1	8.7–11.1	10.6
Upper jaw length	26.8	24.6–29	27.2	29.3	27.2–30.1	30.3	26.7–33.8	28.8
Lower jaw length	36.6	34.3–39.1	37.8	38.7	37.8–39.1	42.2	40–44.4	43.5
Length of maxilla	18.9	17.3–21.7	21.1	19.7	18.6–21.1	23.1	20.1–24.7	22
Snout depth	10.2	9.3–11.9	9.7	10.9	9.7–12.3	7.4	5.5–9.5	6.8
Snout width	15.1	12.3–17.6	14.9	17.8	14.9–18.5	15.6	14.1–17.4	15
Head width	56.1	46.7–62.3	51.8	50.8	50.5–51.8	41.6	39.3–43.3	45.5
Interorbital width	28.4	26.2–31.6	29.6	29.7	28.8–30.8	26.1	23.8–28.9	24.2
Internarial width	11.9	10.7–12.7	12.0	13.7	12–13.8	11.9	10.7–14.1	12.7
Lower jaw length	7.7	6.8–9.9	7.8	8.1	7.8–8.5	7.8	6.4–8.8	8.1
Lower jaw width	25.2	23.1–26.8	24.9	26.4	24.9–27.2	21.6	18.6–24.6	22.7
Middle gill raker length	6.9	4.1–8.7	9.9	10.2	9.8–10.6	12.5	11.6–14.7	10.8
Longest gill raker length	8.2	6.7–10.6	10.9	11.5	10.9–12	14.6	13.3–16.1	11.3
Upper arch length	na	na	na	na	na	na	na	na
Lower arch length	na	na	na	na	na	na	na	na

*N=9forCF, TL, MGR, LGR; **N=2forCF, TL, MGR, LGR; ***N=6forMW; N=5for AFAe, M, INW, LJW; N=4forMGR, LGR.

**Table 10.** The first- and second-best ratios retrieved from the LDA ratio extractor of pair-wise comparisons of all or a subset of head and body characters of the contemporary specimens from the species of lakes Lucerne and Sarnen. For some comparisons only a subset of characters could be used (a-j); the respective characters that were included are listed at the end of the table. δ is a measure of how good shape discriminates in comparison to size (i.e., the smaller the less allometry). Ratios marked with an asterisk * have very little (not more than one specimen of one species overlaps with the other species) or no overlap and were thus eligible for use in the species key and the diagnoses.

Species comparison	Best ratio	Range species 1	Range species 2	Standard distance	δ (shape vs. size)
*C.litoralis* vs. *C.intermundia* (a)	PreD/ED *	10.89–12.75	9.92–10.59	19.5	0.17
	SN/M *	1.17–1.39	1.02–1.17	18.95	0.18
*C.litoralis* vs. *C.suspensus* (b)	CD/UJW *	1.74–1.97	1.5–1.68	11.98	0.27
	CD/PreD *	0.16–0.18	0.15–0.16	11.43	0.28
*C.litoralis* vs. *C.nobilis* (c)	CD/PostD *	0.17–0.21	0.14–0.16	54.78	0.06
	PecF2/DFAe *	0.84–0.92	0.94–1.10	54.52	0.06
*C.litoralis* vs. *C.muelleri* (d)	CD/ED *	1.87–2.25	1.12–1.43	34.61	0.24
	HD/SW *	4.45–5.55	3.45–4.45	33.84	0.25
**Species comparison**	**Best ratio**	**Range species 1**	**Range species 2**	**Standard distance**	δ **(shape vs. size)**
*C.intermundia* vs. *C.suspensus* (e)	PreD/ED *	9.92–10.59	10.73–11.4	14.96	0.01
*C.intermundia* vs. *C.nobilis* (f)	CD/HW *	0.77–0.87	0.63–0.77	13.81	0.06
*C.intermundia* vs. *C.muelleri* (g)	CD/ED *	1.53–1.84	1.12–1.43	16.58	0.28
**Species comparison**	**Best ratio**	**Range species 1**	**Range species 2**	**Standard distance**	δ **(shape vs. size)**
*C.suspensus* vs. *C.nobilis* (h)	PecFB/CD *	0.41–0.43	0.45–0.52	49.76	0.02
*C.suspensus* vs. *C.muelleri* (i)	PreD/ED *	10.73–11.4	8.17–9.63	28	0.19
	BD/LJ *	2.71–3.33	2.01–2.63	27.46	0.2
**Species comparison**	**Best ratio**	**Range species 1**	**Range species 2**	**Standard distance**	δ **(shape vs. size)**
*C.nobilis* vs. *C.muelleri* (j)	ED/HD *	0.27–0.33	0.35–0.42	29.85	0.21
**Species comparison**	**Best ratio**	**Range species 1**	**Range species 2**	**Standard distance**	δ **(shape vs. size)**
*C.sarnensis* vs. *C.litoralis* (k)	ED/HD *	0.33–0.39	0.25–0.30	99.33	0.04
*C.sarnensis* vs. *C.intermundia* (l)	SL/ED	16.29–21.67	20.59–24.04	19.6	0.07
*C.sarnensis* vs. *C.suspensus* (m)	PreD/ED *	7.42–10.21	10.73–11.40	7.24	0.17
	PelvF/PreA *	0.20–0.24	0.20–0.21	6.56	0.17
*C.sarnensis* vs.*C.nobilis* (n)	PostD/EC *	5.94–7.94	7.87–9.77	14.28	0.14
**Multispecies comparison**					
*C.muelleri* vs. *C.intermundia + C.nobilis* (o)	TL/ED *	21.37–24.65	24.45–29.59	9.75	0.40

(a) PecF2, DFB, DFAe, DFAd, DFPe, CD, CL, PAdC, DHL, PreP, PreA, PreD, PostD, SN, ED, EH, HL, MW, UJ, LJ, M, SW, HW, UJW, ES (b) DFAe, CD, PAdC, DHL, PreP, PreA, PreD, PostD, SN, ED, HL, MW, UJ, LJ, M, UJW (c) PelvF, PecF2, DFB, DFAe, DFAd, DFPe, AFAe, AdFB, CD, CL, PAdC, DHL, PreP, PreA, SL, PreD, PostD, SN, ED, EC, EH, HL, HD, MW, UJ, LJ, M, SW, HW, IOW, UJW, ES (d) all characters except PelvFS, SL, INW, CF, TL (e) PelvFB, CD, DHL, PreP, PreA, PreD, PostD, SN, ED, HL, MW, UJ, LJ, M, SD, UJW, ES (f) PelvF, PecF2, DFB, DFAe, DFAd, DFPe, AFAe, CD, CL, PAdC, DHL, PreP, PreA, PreD, BD, PostD, SN, ED, EH, PostO, HL, HD, MW, UJ, LJ, M, SW, HW, IOW, LJW, UJW, ES (g) all characters except CF, TL (h) PelvFB, PELVF, PECFB, PECF2, DFAe, CD, CL, DHL, PreP, PreA, PreD, BD, SN, ED, PostO, MW, LJ, M, SD, SW, IOW, INW, LJW (i) PelvF, PecF2, DFB, DFAe, DFAd, DFPe, AFB, AFAe, CD, CL, PAdC, DHL, PreP, PreA, PreD, BD, PostD, SN, ED, EH, PostO, HD, HL, MW, UJ, LJ, M, SD, SW, HW, IOW, UJW, ES (j) PelvF, PecF2, DFAe, DFAd, AFB, AFAe, CD, CL, DHL, PreP, PreD, BD, PostD, SN, ED, PostO, HL, HD, LJ, M, SD, SW, HW, IOW, UJW, ES (k) all characters except TL, CF, EC, PecF1, AdFB (l) all characters except TL, CF, EC, PecF1 (m) PelvFB, PelvF, PecFB, PecF2, DFAe, CD, CL, DHL, PreP, PreA, PreD, BD, SN, ED, PostO, MW, LJ, M, SD, SW, IOW, INW, LJW (n) all characters (o) all characters.

##### ﻿ Coregonus gutturosus

Taxon classificationAnimaliaSalmoniformesCoregonidae

Gmelin, 1818

8B3DFA53-885E-50D6-8286-F5468D07DA62

###### Historical specimens

(years 1940, 1950): ***Non-types*.**NMBE-1076230 (Eawag-246), NMBE-1076232 (Eawag-248-1), NMBE-1076233 (*N* = 6: Eawag-249-1, Eawag-249-2, Eawag-249-3, Eawag-249-4, Eawag-249-5, Eawag-249-6), NMBE-1076232 (*N* = 2: Eawag-248-2, Eawag-248-3), *N* = 10, 169–292 mm SL.

**Table 11.** The first- and second-best ratios retrieved from the LDA ratio extractor of pair-wise comparisons of a subset of head and body characters of the historical specimens from the species of lakes Lucerne and Zug. For all comparisons only a subset of characters could be used (a-f); the respective characters that were included are listed at the end of the table. δ is a measure of how good shape discriminates in comparison to size (i.e., the smaller δ the less allometry). Ratios marked with an asterisk * have very little (not more than one specimen of one species overlaps with the other species) or no overlap and were thus eligible for use in the species key and the diagnoses.

Species comparison	Best ratio	Range species 1	Range species 2	Standard distance	δ (shape vs. size)
*C.litoralis* vs. *C.muelleri* (a)	CD/DHL *	0.46–0.58	0.37–0.44	25.04	0.11
*C.litoralis* vs. *C.supersum* (b)	MW/SD *	1–1.21	0.75–1	97.44	0.003
*C.litoralis* vs. *C.obliterus* (c)	PecF2/EC *	2.69–3.64	2.46–2.72	9.35	0.1
	AdFB/EC *	0.89–1.17	0.49–0.87	8.85	0.1
*C.supersum* vs. *C.zugensis (d)*	DFB/UJ *	1.94–2.52	1.27–1.9	16.92	0.14
*C.supersum* vs. *C.obliterus (e)*	PreD/EH *	10.52–12.07	8.46–9.73	6.77	0.07
	AdFB/ED*	1.15–1.42	0.64–1	5.67	0.08
*C.zugensis vs. C.obliterus* (f)	EC/M *	1.11–1.34	1.38–1.65	28.1	0.13
*C.zugensis* vs. *C.muelleri* (g)	CD/PreP *	0.13–0.15	0.12–0.13	5.38	0.16

(a) PecF2, DFB, AdFB, CD, DHL, EC, HL, MW, UJ, M, SW, UJW, ES (b) CD, DHL, AFB, PostO, MW, UJ, LJ, M, HL, SD, SW (c) DFPe, CD, PAdC, PreP, TL, PreD, EC, PostO, HD, UJ, LJ (d) PelvFB, PelvF, PecF1, DFB, DFAe, AdFB, UJW, DHL, PostD, SN, EC, PostO, MW, UJ, ES (e) AdFB, CD, CL, DHL, PreA, PreD, PostD, SN, ED, EH, HL (f) PelvFB, PelvF, PelvFS, PecF2, DFB, DFPe, UJW, HL, DHL, PostD, SN, EC, PostO, MW, LJ, M (g) PelvFB, PelvF, PecF1, DFB, DFAe, AdFB, CD, DHL, PreP, PostD, SN, EC, PostO, MW, UJ, UJW, ES.

##### ﻿ Coregonus arenicolus

Taxon classificationAnimaliaSalmoniformesCoregonidae

Kottelat, 1997

B347496D-3707-59E3-B038-5C7172043F50

###### Historical specimens

(year 1950): ***Holotype*.**NMBE-1076223 (Eawag-239-1), 296 mm SL, sex unknown; Switzerland: Lake Constance. ***Paratypes*.**NMBE-1076223 (*N* = 3: Eawag-239-2, Eawag-239-3, Eawag-239-4), *N* = 3, 289–314 mm SL.

**Table 12.** Frequency of occurrence of lateral line, predorsal and transverse dorsal, anal and pelvic scales (mode in bold) in the 13 whitefish species from lakes Lucerne (*C.litoralis*, *C.intermundia*, *C.suspensus*, *C.nobilis*, *C.muelleri*,), Sarnen (*C.sarnensis*), Sempach (*C.suidteri*), Zug (*C.zugensis*, *C.obliterus*, *C.supersum*), and Constance (*C.gutturosus*, *C.arenicolus*, *C.macrophthalmus*, *C.wartmanni*).

Species	Lake	Ntotal	Number of lateral line scales																									
			71	72	73	74	75	76	77	78	79	80	81	82	83	84	85	86	87	88	89	90	91	92	93	94	95	96
*C.litoralis* contemporary	Lucerne	13										1	2			1	**3**	1			1		1	**3**				
*C.litoralis* historical	Lucerne	7											1					1	1	**2**				1	1			
*C.intermundia*	Lucerne	11							1	1		1				1	1			1	**2**			1	**2**			
*C.suspensus*	Lucerne	5										1			1		1			1			1					
*C.nobilis* contemporary	Lucerne	21													1	**4**	1	2	2	**4**	2	3					1	1
*C.nobilis* historical	Lucerne	3												1			1		1									
*C.muelleri* contemporary	Lucerne	30				1	1		3	3	5	**9**	3	1		1		1	1						1			
*C.muelleri* historical	Lucerne	8	1								1	1	1	1	1				1	1								
*C.sarnensis*	Sarnen	28							1	1	**4**			3	3	1	3	2	3	1	1	3		1	1			
*C.suidteri*	Sempach	2													1			1										
*C.zugensis*	Zug	12			1	1	**2**		1		1	1	1			1	1		1					1				
*C.obliterus*	Zug	7		1	1		1	1			1				1		1											
*C.supersum*	Zug	6					**2**		1									1	1		1							
*C.gutturosus*	Constance	10						1	2	**3**	1	2		1														
*C.arenicolus*	Constance	4												1				1			1	1						
*C.macrophthalmus*	Constance	7			1			1		1	1	**3**																
**Species**	**Lake**	**Ntotal**	**Number of predorsal scales**																									
			**26**	**27**	**28**	**29**	**30**	**31**	**32**	**33**	**34**	**35**	**36**	**37**	**38**	**39**	**40**	**41**	**42**	**43**	**44**							
*C.litoralis* contemporary	Lucerne	13				1	1	1	**2**	1	1	4	1	1														
*C.litoralis* historical	Lucerne	7									**3**	2	2															
*C.intermundia*	Lucerne	11					2	1	2		1	1	**3**		1													
*C.suspensus*	Lucerne	5						1	2	1	1																	
*C.nobilis* contemporary	Lucerne	21						4		1	5	**6**	1	4														
*C.nobilis* historical	Lucerne	3									**2**			1														
*C.muelleri* contemporary	Lucerne	30	2		4	4	6	**7**	2	3		1	1															
*C.muelleri* historical	Lucerne	8					2		**3**	2	1																	
*C.sarnensis*	Sarnen	28				1	1	1	3	**6**	5	5	4	1	1													
*C.suidteri*	Sempach	2													1						1							
*C.zugensis*	Zug	12			1	2	2	2	**3**	2																		
*C.obliterus*	Zug	5			1		1	1		1			1															
*C.supersum*	Zug	6							1		**3**	1			1													
*C.gutturosus*	Constance	9						1	3	**4**		1																
*C.arenicolus*	Constance	4											1	1			1				1							
*C.macrophthalmus*	Constance	7							**3**	2	1		1															
*C.wartmanni*	Constance	1									1																	
			**Number of scales**																									
**Species**	**Lake**	**Ntotal**	**transverse dorsal**				**transverse anal**				**transverse pelvic**																	
			**8**	**9**	**10**	**11**	**7**	**8**	**9**	**10**	**7**	**8**	**9**	**10**														
*C.litoralis* contemporary	Lucerne	13		1	**9**	3	1	5	**7**			5	**7**	1														
*C.litoralis* historical	Lucerne	7			**4**	3	1	**3**	2	1	1	**3**	2	1														
*C.intermundia*	Lucerne	11	1	4	**5**	1	2	**5**	4			3	**6**	2														
*C.suspensus*	Lucerne	5	1	**3**	1		1	**4**				2	**3**															
*C.nobilis* contemporary	Lucerne	21		**11**	10		**13**	7	1			1	**18**	2														
*C.nobilis* historical	Lucerne	3		**3**			1	2				**2**	1															
*C.muelleri* contemporary	Lucerne	30	2	**22**	6		5	**20**	5			**18**	10	2														
*C.muelleri* historical	Lucerne	8	3	**5**			1	**7**			1	**6**	1															
*C.sarnensis*	Sarnen	28	3	11	**14**		4	**16**	8			1	10	**17**														
*C.suidteri*	Sempach	2			2																							
*C.zugensis*	Zug	12	**4**	**4**	**4**		4	**5**	3		2	**5**	**5**															
*C.obliterus*	Zug	7		1	**5**	1		**5**	2			**5**	2															
*C.supersum*	Zug	6	1	1	**4**		1	**5**				2	**4**															
*C.gutturosus*	Constance	9	4	**5**			**4**	3	2			**8**	1															
*C.arenicolus*	Constance	4			**3**	1		1	**3**			1	**3**															
*C.wartmanni*	Constance	1			1			1				1																

##### ﻿ Coregonus macrophthalmus

Taxon classificationAnimaliaSalmoniformesCoregonidae

Nüsslin, 1882

E71AAC33-ACDA-50B1-BFB0-BEE0D03227F1

###### Historical specimens

(years 1895, 1901, 1907, 1921): ***Syntypes***: MHNG 716.052, MHNG-716.051, MHNG-816.02, MHNG-715.094 (*N* = 2: MHNG-715.094), NMBE-1076211 (*N* = 2: Eawag-227-1, Eawag-227-2), *N* = 7, 193–235 mm SL.

**Table 13.** Frequency of occurrence of the total number of gill rakers (mode in bold) in the 13 whitefish species from lakes Lucerne (*C.litoralis*, *C.intermundia*, *C.suspensus*, *C.nobilis*, *C.muelleri*,), Sarnen (*C.sarnensis*), Sempach (*C.suidteri*), Zug (*C.zugensis*, *C.obliterus*, *C.supersum*), and Constance (*C.gutturosus*, *C.arenicolus*, *C.macrophthalmus*, *C.wartmanni*).

Species	Lake	Ntotal	Total number of gill rakers																											
			16	17	18	19	20	21	22	23	24	25	26	27	28	29	30	31	32	33	34	35	36	37	38	39	40	41	42	43
*C.litoralis* contemporary	Lucerne	13									1		**3**	2	1	1	**3**	1	1											
*C.litoralis* historical	Lucerne	7				1				1		1			1	1	1			1										
*C.intermundia*	Lucerne	10															2			1	2	**3**		2						
*C.suspensus*	Lucerne	4																		1	1	1		1						
*C.nobilis* contemporary	Lucerne	21																			2		2	4	**8**	3	2			
*C.nobilis* historical	Lucerne	1																										**1**		
*C.muelleri* contemporary	Lucerne	27																		1	1	3	1	5	**6**	5	2	2		1
*C.muelleri* historical	Lucerne	5																					1	**2**		**2**				
*C.sarnensis*	Sarnen	28																		1	4	**7**	**7**	5	3		1			
*C.suidteri*	Sempach	1																				1								
*C.zugensis*	Zug	12																		2	1	**3**	1	**3**	1		1			
*C.obliterus*	Zug	7						1	1	1	1	**2**	1																	
*C.supersum*	Zug	5						1		1	1		1	1																
*C.gutturosus*	Constance	9	1	**2**	**2**	**2**	1	1																						
*C.arenicolus*	Constance	3							1			1						1												
*C.macrophthalmus*	Constance	4																					**2**	1	1					

##### ﻿ Coregonus wartmanni

Taxon classificationAnimaliaSalmoniformesCoregonidae

Bloch, 1784

CF19C408-D928-5A2A-999F-43E6525B40B8

###### Historical specimen

(year 1895): ***Non-type***: NMBE-1076206, Switzerland, 301 mm SL.

### ﻿Identification key to the species of Lake Lucerne

This identification key is based on contemporary specimens of the five species from Lake Lucerne. The key leads in two distinct ways to *C.nobilis*. The species key needs to be followed completely to be able to distinguish *C.nobilis* from all other whitefish species of Lake Lucerne.

**Table:** 

1	Predorsal length / eye diameter ratio is 10.73–12.75	**2**
–	Predorsal length / eye diameter ratio is 8.17–10.59	**4**
2	Total number of gill rakers is 24–32	***C.litoralis***
–	Total number of gill rakers is 33–40	***3***
3	Pectoral fin base / caudal peduncle depth ratio is 0.41–0.43	***C.suspensus***
–	Pectoral fin base / caudal peduncle depth ratio is 0.45–0.52	***C.nobilis***
4	Total length / eye diameter ratio is 21.37–24.45	***C.muelleri***
–	Total length / eye diameter ratio is 24.5–29.59	**5**
5	Caudal peduncle depth / head width ratio is 0.77–0.87 and the erected anterior part of the dorsal fin is larger than 17.3–19.9% SL	***C.intermundia***
–	Caudal peduncle depth / head width ratio is 0.63–0.77 and the erected anterior part of the dorsal fin is 15.3–17.2% SL	***C.nobilis***

## ﻿Discussion

All pre-alpine whitefish species have been shown based on phylogeographic studies to belong to a monophyletic clade of hybrid origin involving two anciently divergent glacial lineages that must have come into secondary contact several hundred thousand years after their separation. Independent events of intra-lacustrine speciation led to a series of adaptive radiations in each major lake system north of the Alps (Hudson et al. 2011). Up to six endemic species can be found in the most diverse of these adaptive radiations (Vonlanthen et al. 2012; Doenz et al. 2018; Selz et al. 2020). Herein we distinguish and characterise ten distinct whitefish species from the Swiss lakes Lucerne, Sarnen, Sempach, and Zug, of which seven species were unnamed.

**Figure 14.**
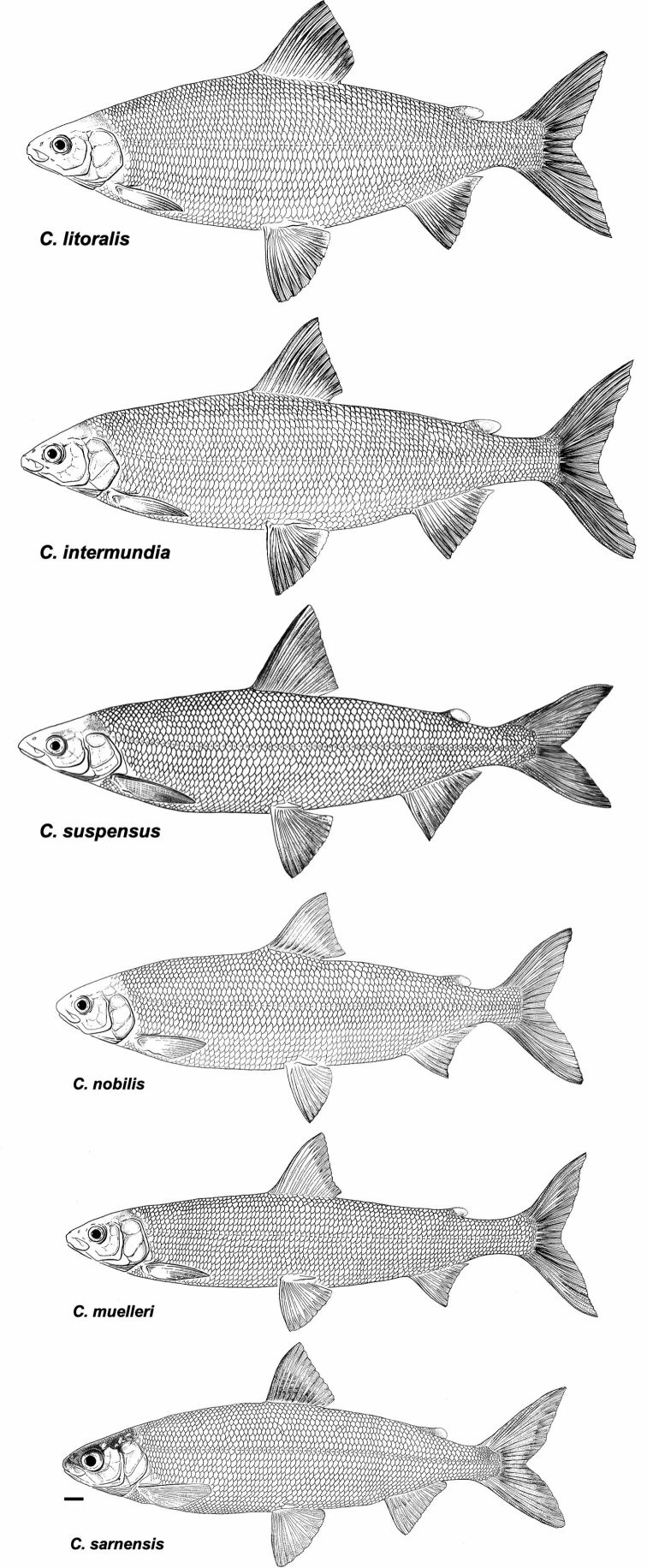
Illustrations of specimens of each species of *Coregonus* from Lake Lucerne and Sarnen. Illustrations of Lake Lucerne species are based on several individuals. Illustration of *C.sarnensis* is based on NMBE-1078159, 230 mm, male. Scale bar for *C.sarnensis*: 1 cm.

We show with two independent genetic datasets (Hudson et al. 2011, 2016; this study) that two of the three re-described species, *C.suidteri* and *C.zugensis*, which were previously thought to naturally occur in several lakes in Switzerland, namely for *C.suidteri* lakes Lucerne, Zug, Sempach, Hallwil, and Baldegg and for *C.zugensis* lakes Lucerne and Zug (Kottelat 1997), are composed of multiple species endemic to different lakes in Switzerland. The names *C.suidteri* and *C.zugensis* are retained for the lakes Sempach and Zug, respectively. The status of the species previously grouped under *C.suidteri* and *C.zugensis* are resolved in this study except for the extinct species C.cf.suidteri from lakes Hallwil and Baldegg. We describe two additional species for Lake Zug, *Coregonussupersum*, which has previously been identified as *C.suidteri* and *C.obliterus*, which was forgotten for more than a century in the scientific literature only to be resurrected in this study due to a re-examination of the Steinmann collection. *Coregonusobliterus* and *C.zugensis* are extinct and most likely *C.suidteri* is also extinct as the extant population of whitefish in Lake Sempach shows strong genetic affiliations to whitefish from other lakes. The third re-described species, *C.nobilis*, is one of five species of the adaptive radiation of Lake Lucerne. Among the other four species from Lake Lucerne, two have previously been identified as *C.suidteri* and *C.zugensis* and which we now describe as *Coregonuslitoralis* and *Coregonusmuelleri*, respectively. The other two species of Lake Lucerne have only been known to science since the work by Lundsgaard-Hansen (2009) and Hudson et al. (2016) and are described as *Coregonussuspensus* and *Coregonusintermundia*. Finally, for lake Sarnen we have described one new species, *Coregonussarnensis*.

**Table 14.** Frequency of occurrence of the number of branched rays of the pelvic, pectoral, dorsal and anal fin (mode in bold) in the 13 whitefish species from lakes Lucerne (*C.litoralis*, *C.intermundia*, *C.suspensus*, *C.nobilis*, *C.muelleri*), Sarnen (*C.sarnensis*), Sempach (*C.suidteri*), Zug (*C.zugensis*, *C.obliterus*, *C.supersum*), and Constance (*C.gutturosus*, *C.arenicolus*, *C.macrophthalmus*, *C.wartmanni*).

Species	Lake	Ntotal	Number of branched rays of																									
			pelvic fin				Ntotal	pectoral fin									Ntotal	dorsal fin					Ntotal	anal fin				
			9	10	11	12		9	10	11	12	13	14	15	16	17		9	10	11	12	13		10	11	12	13	14
*C.litoralis* contemporary	Lucerne	13		**7**	6		13						5	**8**			13		1	**10**	2		13		**7**	6		
*C.litoralis* historical	Lucerne	7	1	**3**	**3**		7		1			1	**2**	1	1	1	6		1	2	**3**		6	1	2	**3**		
*C.intermundia*	Lucerne	11		5	**6**		10						2	**7**	1		11		**5**	3	2	1	11		2	**9**		
*C.suspensus*	Lucerne	5		**4**	1		5						1	**3**	1		5		**2**	**2**	1		5		1	**3**	1	
*C.nobilis* contemporary	Lucerne	21	2	**19**			21							3	**18**		21		9	**10**	2		21		9	**11**	1	
*C.nobilis* historical	Lucerne	3		2	1		2								**2**		3	1	**2**				3		1	**2**		
*C.muelleri* contemporary	Lucerne	30	2	**25**	3		27						1	**16**	10		30	3	**15**	10	2		30		3	**19**	6	2
*C.muelleri* historical	Lucerne	8		**5**	3		8							**5**	3		8	2	2	**4**			8	2	**3**	**3**		
*C.sarnensis*	Sarnen	28		**17**	11		28						5	**16**	7		28	3	**21**	4			28	2	8	**18**		
*C.suidteri*	Sempach	2		1	1		2							1	1		2			1	1		2		1	1		
*C.zugensis*	Zug	12	1	**8**	3		12						2	3	**6**	1	12	1	**9**	2			12	2	2	**5**	2	1
*C.obliterus*	Zug	7		**7**			7					**4**	1	1	1		7		**3**	**3**	1		7	**2**	1	**2**	**2**	
*C.supersum*	Zug	6		**4**	2		6	1					1	**4**			1		1	**3**	2		1	1	**3**	2		
*C.gutturosus*	Constance	9	1	3	**5**		9				2	**4**	3				9	1	**8**				9	2	**5**	2		
*C.arenicolus*	Constance	4			**4**		4				**2**	1	1				4	1	**3**				4	**3**	1			
*C.macrophthalmus*	Constance	7		**4**	3		7						3	**4**			7	**4**	3				7	1	**3**	1	2	
*C.wartmanni*	Constance	1				1	1								1		1		1				1				1	

Many Swiss lakes experienced strong anthropogenic-induced eutrophication which among the lakes in this study was pronounced in lakes Zug and Sempach. It has been accompanied by population collapse, speciation reversals, and extinction of Swiss whitefish (Vonlanthen et al. 2012; Frei et al. 2022a, b). Furthermore, many Swiss lakes were also deliberately stocked with whitefish species from Swiss lakes as well as from European and North American lakes resulting in hybridization that has left introgressive signals of non-native genetic material in extant populations in Swiss lakes (Lundsgaard-Hansen 2009; Hudson et al. 2016; Doenz et al. 2018; Selz et al. 2020; De-Kayne et al. 2022) (this is discussed in more detail in the Suppl. material 1: paragraph 5). These deliberate translocations of non-native whitefish species can be seen today in *C.suspensus* which shows genetic ancestry contributions from whitefish of Lake Constance, besides its Lake Lucerne ancestry. Similarly, strong genetic signals of Lake Lucerne and Lake Zug whitefish can be seen in the extant population of Lake Sempach suggesting that this population does not represent the original population of *C.suidteri*. It also has cast doubt if the occurrence of *C.litoralis* in lake Sarnen, which was part of Lake Lucerne before it’s separation due to the amassing of sediments through several rivers, is of natural origin or due to such translocations. Scales retrieved from sediment cores prior to such translocations suggest that Lake Sarnen did harbour an endemic whitefish species, which we could show with contemporary specimens is genetically distinct and which we have described as *C.sarnensis*, but this does not resolve the origin of *C.litoralis* in Lake Sarnen. Furthermore, the many introductions of non-native whitefish into the mesotrophic Lake Alpnach, which is a side-arm of the oligotrophic Lake Lucerne, may explain why the whitefish from this part of the lake group genetically with whitefish from Lake Sempach and Lake Zug (Suppl. material 1: fig. S3). There has been allot of uncertainty in the scientific literature regarding the species status of the whitefish in Lake Alpnach due to the many introductions of allochthonous whitefish coupled with the large phenotypic variability found in this population (Birrer and Schweizer 1936b; Steinmann 1950; Svarvar and Müller 1982; Hudson et al. 2016). A future study incorporating a quantitative sampling campaign coupled with high throughput genomic sequencing may be able to resolve the species status of the whitefish population found in Lake Alpnach. The lack of such data did not allow us to examine if the population of whitefish found in Lake Alpnach is composed of several native whitefish with possible genetic signatures from non-native introductions or if it harbours a unique whitefish population. The translocations of Swiss whitefish have also led to the occurrence of whitefish from lakes Zug and Sarnen in two southern Swiss lakes, which naturally did not harbour whitefish. Hudson et al. (2011) could show that the populations of whitefish that are present in Lake Maggiore (local name of the population is ‘Lavarello’) and Lake Lugano group in a neighbour-joining tree with the extant population of whitefish from Lake Zug, *C.supersum*, and a second population of Lake Maggiore (local name of the population is ‘Bondella’) group with the whitefish species from Lake Sarnen, *C.sarnensis*. Lake Zug and Lake Sempach have experienced strong anthropogenic-induced eutrophication, which is often accompanied by population collapse, speciation reversal, and extinction of endemic whitefish species (Vonlanthen et al. 2012; Frei et al. 2022a, b). This has most likely also been one of the main drivers of extinction of *C.zugensis* and *C.obliterus* in Lake Zug and possibly of *C.suidteri* in Lake Sempach. In the case of Lake Zug nothing is known about the possibility of speciation reversal, whereby extinct species leave traces in genomes of extant species through introgressive hybridisation. Future research will be needed to understand if the extant whitefish population of Lake Zug and the extant populations of the lakes Maggiore and Lugano that we group to the species *C.supersum* shows signs of introgression of the extinct species of Lake Zug, *C.zugensis* and *C.obliterus*, respectively.

Many of the unresolved Swiss whitefish diversity has been revised with this study coupled with the recent revision by Selz et al. (2020) on the whitefish diversity of lakes Thun and Brienz. However, future work using a taxonomic approach including recent advances in ancient DNA methods is still warranted to resolve the species status of extant and extinct populations in several Swiss lakes.

## Supplementary Material

XML Treatment for Coregonus litoralis

XML Treatment for Coregonus intermundia

XML Treatment for Coregonus suspensus

XML Treatment for Coregonus nobilis

XML Treatment for Coregonus muelleri

XML Treatment for Coregonus sarnensis

XML Treatment for Coregonus supersum

XML Treatment for Coregonus zugensis

XML Treatment for Coregonus obliterus

XML Treatment for Coregonus suidteri

XML Treatment for Coregonus gutturosus

XML Treatment for Coregonus arenicolus

XML Treatment for Coregonus macrophthalmus

XML Treatment for Coregonus wartmanni

## Supplementary materials

Supplementary material 1 Historical and contemporary notes on *C.sarnensis*, *C.litoralis*, *C.nobilis*, Lake Zug whitefish and on different topics in the discussion of the main manuscript. Data type morphological, phylogenetic, paleolimnology Explanation note Genetic diversity estimates from all species of ten lakes. Genetic differentiation (Fst) between pre-alpine whitefish populations based on 10 neutral microsatellite loci. Size-at-age of 3-year-old specimens of *C.sarnensis* from Lake Sarnen and *C.litoralis*, *C.suspensus*, *C.intermundia* and *C.muelleri* from Lake Lucerne. STRUCTURE plot showing individual assignment likelihoods based on 10 microsatellite markers for the whitefish species from Lakes Lucerne, Sarnen, Sempach and Zug. Population-based neighbour-joining tree using Cavalli-Sforza distances based on 10 microsatellite markers. https://binary.pensoft.net/file/804010This dataset is made available under the Open Database License (http://opendatacommons.org/licenses/odbl/1.0/). The Open Database License (ODbL) is a license agreement intended to allow users to freely share, modify, and use this Dataset while maintaining this same freedom for others, provided that the original source and author(s) are credited.Oliver M. Selz, Ole Seehausen
