# Supplementary material for: ﻿A taxonomic revision of ten whitefish species from the lakes Lucerne, Sarnen, Sempach and Zug, Switzerland, with descriptions of seven new species (Teleostei, Coregonidae)
Source: Zookeys. 2023 Feb 2;1144:95–169. doi: 10.3897/zookeys.1144.67747 (PMC10209427; doi:10.3897/zookeys.1144.67747)
Supplement: Supplementary material 1 — Historical and contemporary notes on C.sarnensis, C.litoralis, C.nobilis, Lake Zug whitefish and on different topics in the discussion of the main manuscript. [file zookeys-1144-095_article-67747__-s001.docx]

**Supplementary material**

**Paragraph 1: Historical and contemporary notes on *C. sarnensis and C. litoralis***

There are several documented historical introductions of allochthonous whitefish from lakes Lucerne, Sempach, and Zug into Lake Sarnen (1888-1900: Heuscher 1900; 1913-1920: Steinmann 1950). This and the fact that Fatio (1890) did not consider Lake Sarnen as a whitefish lake had led Heuscher (1900) to suggest that the whitefish species from Lake Sarnen was not endemic to the lake but is of allochthonous origin. Heuscher (1900) suggested that Lake Sarnen harbours a large-type whitefish species (he uses the name *C. schinzii helveticus* and the German name ‘Balchen’). However, Steinmann (1950) noted that Lake Sarnen already harboured whitefish before the allochthonous introductions of whitefish reported in Heuscher (1900). Steinmann (1950) described that a local fisherman called Näpflin was already fishing for whitefish in Lake Sarnen in the first half of the 19^th^ century. Shortly thereafter commercial fishing in Lake Sarnen was abandoned and it was only in the 1890’s that Dr. Ettlin from Sarnen sent whitefish specimens to Fatio (Steinman 1950); therefore, these whitefish specimens did not make it into the compendium of Fatio (1890). Fatio did mention these specimens in short communications and suggested that two whitefish species were present in Lake Sarnen, which he described as a large-type whitefish ‘*Coregonus schinzii helveticus’* and a middle-sized whitefish species ‘*Coregonus wartmanni nobilis*’ (Steinmann 1950). Similarly, Steinmann (1950) also suggested that Lake Sarnen harbours two distinct whitefish species, a middle-sized whitefish species that he named ‘der kleine Sarnerseefelchen (Sarnerbalchli)’ and a large-type whitefish species that he named ‘der grössere Sarnerfelchen’.

Lake Sarnen was once part of Lake Lucerne (connected through Lake Alpnach) and was disconnected by the amassing of sediments from the rivers Grosse Schlieren, Kleine Schlieren, and Grosse Melchaa several thousand years ago (Steinmann 1950). It is thus plausible that Lake Sarnen may harbour an endemic population of whitefish. Indeed, the occurrence of whitefish scales in sediment cores from Lake Sarnen that predate (scales found in the sediment layer of 1861-1857; Suppl. material 1: Figure S1) the earliest allochthonous whitefish introductions (1888) and the independent genetic grouping of the Lake Sarnen population (AFLP data: Hudson et al. 2011; Microsatellite data: Suppl. material 1: Figures S2, S3) from the lakes that seeded the allochthonous whitefish introductions (Lakes Sempach, Zug, Lucerne) suggest that Lake Sarnen harbours an endemic whitefish species. However, we have also been able to genetically assign a second species to Lake Sarnen, namely *C. litoralis* (Suppl. material 1: Figures S2, S3). It is unclear if *C. litoralis* occurs naturally in Lake Sarnen, as the lake was once connected to Lake Lucerne via Lake Alpnach or if the population of *C. litoralis* in Lake Sarnen derives from deliberate introductions of whitefish from Lake Lucerne that were reported by Heuscher (1900) and Steinmann (1950). The three individuals that we could assign genetically to *C. litoralis* were caught outside of the spawning season and clearly phenotypically resembled (e.g., gill raker numbers below 30, deep bodied) *C. litoralis* from Lake Lucerne (Vonlanthen and Périat 2018). So far, *C. litoralis* has not been caught in Lake Sarnen during the typical spawning season of this species in Lake Lucerne (December) and it is thus unknown at which water depth the species spawns (A. von Deschwanden, pers. comm.). *Coregonus litoralis* seems to be rare in Lake Sarnen (Vonlanthen and Périat 2018; A. von Deschwanden, pers. comm.).

**Paragraph 2: Historical and contemporary notes on *C. litoralis***

Historical notes from Nufer (1905) suggest that *C. litoralis* used to be present in large numbers in Lake Alpnach during the spawning season, but diminished over time until it completely vanished. Lake Alpnach is connected with Lake Lucerne by only a 140-m wide, 300-m long, and 2–4-m deep narrow channel. However, Svarvar and Müller (1982) could show that *C. litoralis* spawned in large quantities in Lake Alpnach during the months of November and December of 1977 and 1978, and they are still caught in large quantities today (A. von Deschwanden, J. Muggli, and A. Blättler, pers. comm.).

**Paragraph 3: Historical and contemporary notes on *C. nobilis***

*Coregonus nobilis* was historically the second most abundant whitefish species in Lake Lucerne before the population collapsed in the 1980s’ due to the eutrophication of the lake in the middle of the 20^th^ century (Birrer and Schweizer 1936a; Müller 2007; Vonlanthen et al. 2012). The species was caught in large quantities during the 1970’s and then the catches abruptly declined (J. Muggli, pers. comm.) to the extent that the species was thought to be extinct in the 1980’s only to be re-discovered in the 2000’s (Müller 2007). Since the species is endangered, a routine monitoring campaign is only conducted every second year and targets a maximum of ten individuals by not setting too many nets at the spawning grounds. These monitoring campaigns have been carried out since the 2000’s and since the re-discovery of *C. nobilis*, ripe specimens have been caught at the spawning grounds (J. Muggli, pers. comm.). *Coregonus nobilis* was, together with *C. muelleri*, the commercially most important whitefish species of Lake Lucerne, which experienced two strong population declines in the last century (Birrer and Schweizer 1936b; Müller 2007). The first decline in population size was attributed to overfishing due to intensive spawning fisheries prior to the year 1900, after which this practice was halted by closing fisheries for *C. nobilis* during the spawning period from 25 July to 1 October (Birrer and Schweizer 1936a). Historical records from Nufer (1905) and Surbeck (1913) state that these mitigation measures resulted in a quick recovery of the whitefish stock of *C. nobilis* which led to a shortening of the closed season for spawning fisheries (25 July – 8 September)*.* The second decline in population size, ultimately resulting in a population collapse and in an assumed extinction of *C. nobilis,* was attributed to anthropogenic-induced eutrophication of Lake Lucerne in the middle of the 20^th^ century (Müller 2007; Vonlanthen et al. 2012). Only one century ago, Swiss lakes harboured approximately 35 endemic species of whitefish, but one third of this original diversity has been lost by the middle of the 20^th^ century due to a combination of speciation reversal through hybridisation and demographic declines, both driven by loss of habitat for foraging and spawning, and possibly of selective regimes, associated with anthropogenic eutrophication of lakes (Vonlanthen et al. 2012; Hudson et al. 2013; Alexander et al. 2017a; Frei et al. 2022). Indeed, stocks of *C. nobilis* declined rapidly during approximately the time period (1970-1980’s) when eutrophication of Lake Lucerne was at its peak (Müller 2007; Vonlanthen et al. 2012). Catch statistics of Lake Lucerne whitefish, which distinguished between ‘small-type’ (i.e., *C. muelleri*), ‘middle-type’ (i.e., *C. nobilis*) and ‘large-type’ (i.e., most likely comprising *C. litoralis* and possibly *C. intermundia* and *C. suspensus*) whitefish, showed a strong population decline of *C. nobilis* up to 1978 (Müller 2007). *Coregonus nobilis* was declared extinct in 1980 and the likely reason for the assumed extinction was attributed to eutrophication-induced oxygen depletion of the deep waters below 100 m resulting in a loss of spawning habitats (Müller 2007; Vonlanthen et al. 2012). From 1995 onwards, rare catches of *C. nobilis* were reported in the local fisheries catches and in 2004 targeted spawning fisheries at the historical spawning grounds yielded a low number of ripe individuals of *C. nobilis* (Müller 2007). Mitigation measures to halt eutrophication such as improved sewage treatment and phosphorus management have allowed some lakes (including Lake Lucerne) to return near to their natural trophic state (Vonlanthen et al. 2012) and were certainly instrumental in the re-emergence of the assumed-to-be-extinct whitefish species *C. nobilis.*

Interestingly, some morphological characters differ between the historical and contemporary specimens of *C. nobilis*. These morphological differences mainly concern fin lengths and eye sizes (longer fins and larger eyes in the historical specimens). Future work will be needed to understand which mechanisms are at play that have resulted in the morphological differences observed in the extant population of *C. nobilis.*

**Paragraph 4: Historical and contemporary notes on Lake Zug whitefish**

According to a table in a short communication by Fatio (1885: table 2) Lake Zug used to harbour three whitefish species, ‘Balchen’, ‘Albock’, and ‘Albeli’. However, in his compendium on Swiss fauna, Fatio (1890) only mentioned two species for Lake Zug, namely ‘Balchen’ (*Coregonus schinzii helveticus* var. *zugensis*) and ‘Albeli-Albock’ (*Coregonus wartmanni compactus*). Fatio (1890) does mention that fishermen had suggested that among the ‘Albeli-Albock’ there were individuals that were smaller and spawned later in the year, to which they referred as ‘Albeli’, compared to the individuals they referred to as ‘Albock’ that are slightly larger and spawned earlier in the year. Fatio (1885) suggested that ‘Albeli’, which the fishermen thought were distinct from the ‘Albock’, are most likely young individuals of the ‘Albeli-Albock’ or a ‘mixture’ between the ‘Albeli-Albock’ and young ‘Balchen’. Thus, based on the specimens that Fatio (1890) examined he suggested to group the two entities under the name ‘Albeli-Albock’. Interestingly, Wagler (1937) noted two species present in Lake Zug with a remark that a third species may exist, but that its status is uncertain. Steinmann (1950) and other authors (e.g., Kottelat and Freyhof 2007; Vonlanthen 2012) suggested that Lake Zug used to harbour two species of whitefish and that *C. zugensis* went extinct. Steinmann (1950) concluded that, by the time he sampled whitefish in Lake Zug in the years 1935-1939, *C. zugensis* was already extinct and that only *C. supersum* (*Coregonus* sp. ‘Zugerbalchen’) was present in the Lake. However, after revisiting the Steinmann-Eawag collection of Steinmann (1950), we noted that a phenotypically peculiar third group of specimens exist in the collection, which could not be grouped to *C. supersum* nor to *C. zugensis* based on morphological characters. We described these specimens as the new species *C. obliterus*. Thus, the ‘Albeli-Albock’ of Fatio (1890) were indeed two species, suggesting that the table in Fatio (1885: table 2) and the anecdotes of local fishermen that distinguished between ‘Albeli’ and ‘Albock’ were correct.

The extinction of *C. obliterus* and *C. zugensis* can be attributed to strong anthropogenic-induced eutrophication that was present in many Swiss lakes, which was accompanied by population collapse, speciation reversal, and the extinction of Swiss whitefish (Vonlanthen et al. 2012; Frei et al. 2022). Steinmann (1950) noted that historical records suggested that catches of the ‘Albeli-Albock’, *C. zugensis*, and *C. obliterus* had already declined in the 1870’s and were attributed to human-induced pollution from tributaries into Lake Zug. In the case of Lake Zug nothing is known about the possibility of speciation reversal, whereby extinct species leave traces in genomes of extant species through introgressive hybridization (Frei et al. 2022). Future research will be needed to understand if the extant whitefish population of Lake Zug that we group to the species *C. supersum* shows signs of introgression of the extinct species of Lake Zug, *C. zugensis* and *C. obliterus*, respectively.

**Paragraph 5: Historical and contemporary notes on different topics in the discussion of the main manuscript**

The supplementary stocking of native whitefish species within a lake may have maintained some of the native whitefish species that had experienced anthropogenic eutrophication in the mid-20^th^ century but overall stocking of both native and non-native whitefish species has negatively impacted the endemic diversity of Swiss whitefish (Vonlanthen et al. 2012; Hudson et al. 2016; Doenz et al. 2018; Selz et al. 2020). Both human-induced eutrophication and translocations of non-native whitefish species have left traces in the extant populations of whitefish species that are present in the lakes studied here. Historical records highlight that there were multiple allochthonous introductions of whitefish into many Swiss lakes (Heuscher 1900; Surbeck 1920; Birrer and Schweizer 1938; Steinmann 1950; Svarvar and Müller 1982; E. Odermatt and J. Muggli, pers. comm.), but these most likely do not encompass the full extent of the human-induced translocations of non-native whitefish species (J. Muggli, pers. comm.) The practice of translocations of non-native whitefish species has been banned in all of Switzerland through federal law only in 1991 (BGF 6 I b). Despite the negative impact that human-induced eutrophication and translocations of non-native whitefish species have had on the endemic Swiss whitefish diversity, Switzerland today still harbours speciose adaptive radiations with up to six species co-occurring in a single lake (Vonlanthen et al. 2012; Doenz et al. 2018; Selz et al. 2020). Only one century ago Swiss lakes harboured approximately 35 endemic species of whitefish, but one third of this original diversity had been lost by the middle of the 20^th^ century.

**Table S1.** Genetic diversity estimates from all species of Lake Lucerne (LU), Lake Sarnen (SA), Lake Sempach (SE) Lake Zug (ZU), Lake Constance (CO), Lake Zurich (ZUE), Lake Walen (WA), Lake Thun (TH), Lake Brienz (BR) and Lake Neuchatel (NE). Abbreviations for the genetic diversity estimates: sample size (N), number of alleles (N_A_), effective number of alleles (N_AE_), heterozygosity (H_O_), expected heterozygosity (H_E_) and inbreeding coefficient (G_IS_). Only one loci (Cocl 49) from Lake Zug deviated from Hardy-Weinberg after sequential Bonferroni correction.

**Table S2.** Genetic differentiation (F_st_) between pre-alpine whitefish populations based on 10 neutral microsatellite loci. We report lake of occurrence (Lake Lucerne, Sarnen, Sempach, Zug, Constance, Walen, Zürich, Neuenburg, Thun, Brienz), species name and sample sizes (N), pairwise *F*_ST_ values (below the diagonal) and the corresponding p-value (above the diagonal).

**Table S3.** Size-at-age of 3-year-old specimens of *C. sarnensis* from Lake Sarnen and *C. litoralis*, *C. suspensus*, *C. intermundia* and *C. muelleri* from Lake Lucerne. No size-at-age is given for *C. nobilis* because for *C. nobilis* only specimens of 5 years of age or older were caught. Size data for Lake Lucerne derive from Hudson et al. (2016) and for Lake Sarnen from this study. Size is measured as standard length and reported in millimetres.

**Figure S1.** Table with accumulated fish scale findings (categories: whitefish (*Coregonus sp*.), perch (*Perca fluviatilis*) and fish scales of unidentified species) in 7 sediment cores from Lake Sarnen (105-135 cm length). Total number of scales or fragments over all 7 cores are given with the symbols for scales or fragments. The longest core (135cm) ranges back to 1795 (based on a net sedimentation rate from dated sediment cores of 0.61cm/y; unpublished). The table is shortened for practical reasons, as the deepest finding was in a sediment depth of 105 cm. The pictured core segment is from SAR18-91, one of the 7 cores. The oldest whitefish scales that were found date back to the years 1849-1845, which suggests that whitefish occurred in Lake Sarnen prior to the deliberate introductions of alevins, fry and adult whitefish from Lake Lucerne, Sempach and Zug. The first introduction from Lake Lucerne and Lake Zug occurred between 1888 to 1900 and the second introductions occurred between 1913 to 1920.

**Figure S2.** STRUCTURE plot showing individual assignment likelihoods based on 10 microsatellite markers for the whitefish species from Lakes Lucerne, Sarnen, Sempach and Zug for the most likely K=4. The genetic legacy of deliberate introductions of alevins, fry and adult whitefish from Lake Lucerne, Sempach and Zug between 1888-1920 into each of the three lakes can still be found today. Despite these introductions species or species-clusters show strong genetic groupings. A few specimens caught in Lake Sarnen had morphological features (e.g. low gill raker numbers) not known for the Lake Sarnen whitefish species *C. sarnensis* and individual assignment likelihoods suggest that these specimens most likely derive from introductions of *C. litoralis* from Lake Lucerne. Also, specimens caught in one of the side-arms of Lake Lucerne, Lake Alpnach, show high individual assignment likelihoods to *C. sarnensis,* suggesting that specimens of *C. sarnensis* succesfully migrate downstream into lake Lucerne.
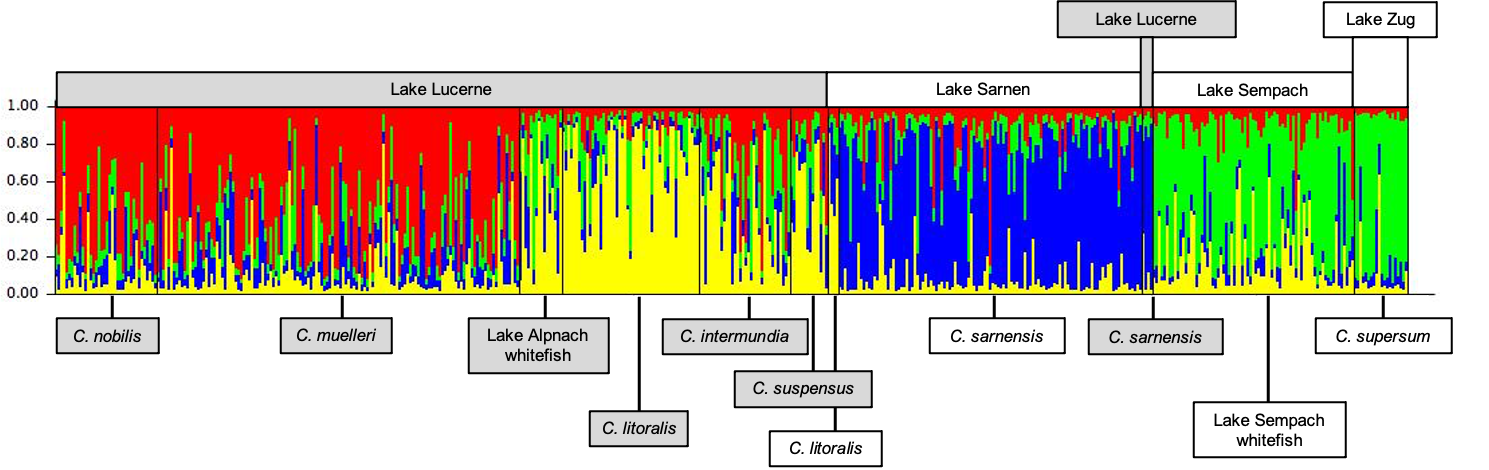


**Figure S3.** Population-based neighbour-joining tree using Cavalli-Sforza distances based on 10 microsatellite markers, showing genetic relatedness among the pre-alpine whitefish species from Lake Lucerne (LU; green), Lake Sarnen (SA; pink), Lake Sempach (SE; grey), Lake Zug (ZU; grey), Lake Constance (CO; yellow), Lake Zurich (ZUE; green), Lake Walen (WA; green), Lake Thun (TH; red), Lake Brienz (BR; red) and Lake Neuchatel (NE; light blue). Most species group together by lake or lake-system. Specimens caught in one of the side-arms of Lake Lucerne, Lake Alpnach, that were genetically assigned in STRUCTURE analysis to *C. sarnensis* (see Figure S4) clearly group in the tree with *C. sarnensis* from Lake Sarnen. Also, specimens from Lake Sarnen, that were genetically assigned in STRUCTURE analysis to one species-group from Lake Lucerne (Figure S4; Structure cluster: *C. litoralis, C. suspensus, C. intermundia* and whitefish from Lake Alpnach) and that had similar low number of gill rakers as *C. litoralis* clearly group in the tree with *C. litoralis* from Lake Lucerne. Furthermore, the two species from lakes Lucerne and Thun, that are of partial allochtonous origin with ancestry contritbution from Lake Constance (Lucerne: *C.* suspensus; Thun: *C.* acrinasus) group with the species from Lake Constance. Colours used for species names, tree branches and representative phenotypes indicate the sampled lakes or lake systems. Bootstrap support values are displayed with different symbols.

**
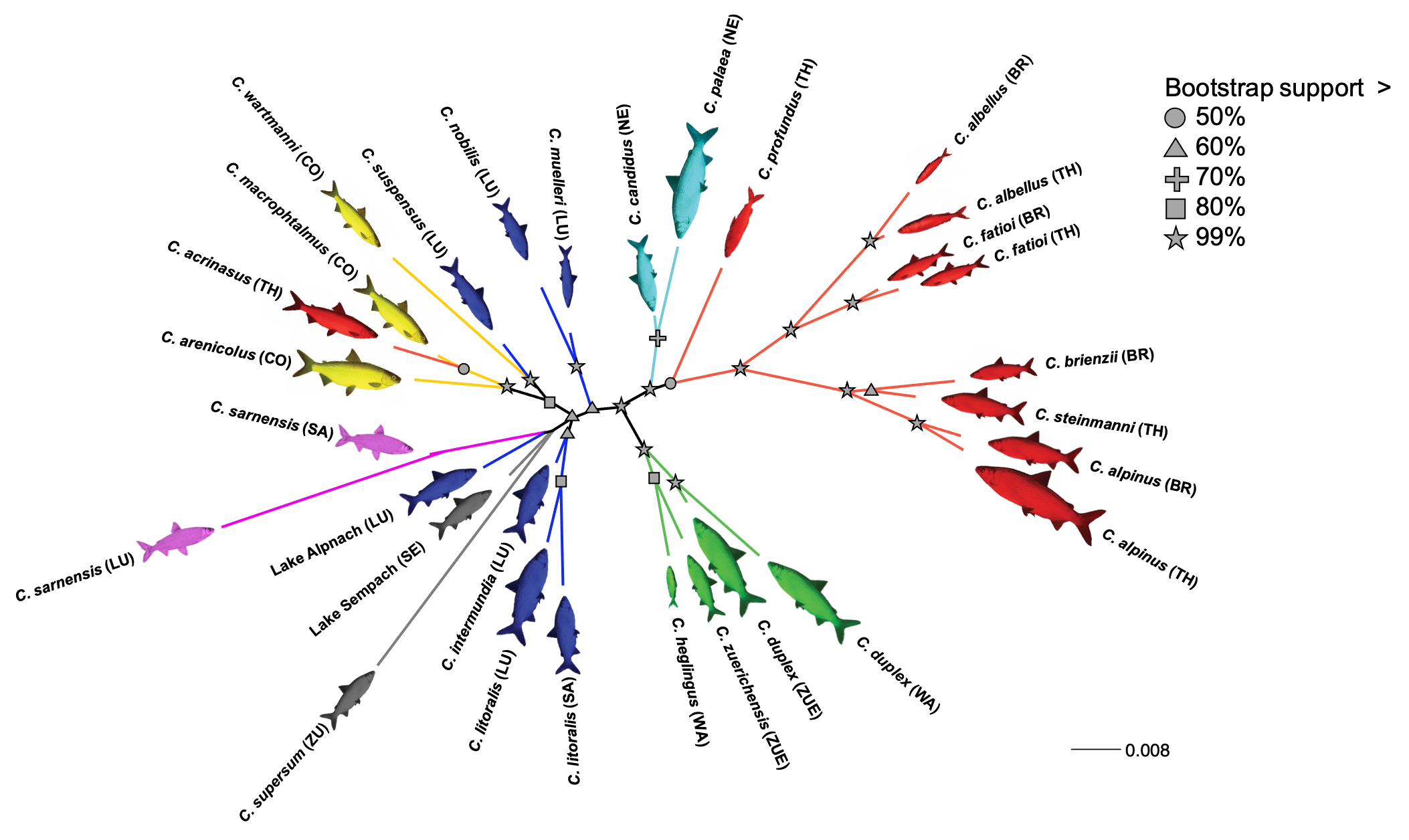
**

**References:** see main manuscript
